# A randomised cross over trial examining the linguistic markers of depression and anxiety in symptomatic adults

**DOI:** 10.1038/s44184-025-00140-y

**Published:** 2025-07-19

**Authors:** Bridianne O’Dea, Philip J. Batterham, Taylor A. Braund, Cassandra Chakouch, Mark E. Larsen, Michael Berk, Michelle Torok, Helen Christensen, Nick Glozier

**Affiliations:** 1 Flinders University Institute for Mental Health and Wellbeing, Flinders University, Adelaide, South Australia Australia; 2 Black Dog Institute, University of New South Wales, Sydney, New South Wales Australia; 3 Centre for Mental Health Research, The Australian National University, Canberra, Australian Capital Territory Australia; 4 Centre for Big Data Research in Health, Faculty of Medicine and Health, University of New South Wales, Sydney, New South Wales Australia; 5 Institute for Mental and Physical Health and Clinical Translation, School of Medicine, Deakin University, Barwon Health, Victoria, Australia; 6 Central Clinical School, Faculty of Medicine and Health, The University of Sydney, Sydney, New South Wales Australia

**Keywords:** Psychology, Anxiety, Depression

## Abstract

Linguistic features within individuals’ text data may indicate their mental health. This trial examined the linguistic markers of depressive and anxiety symptoms in adults. Using a randomised cross over trial design, 218 adults provided eight different types of text data of varying frequencies and emotional valance. Linguistic features were extracted using LIWC-22 and correlated with self-reported symptoms. Machine learning was used to determine associations. No linguistic features were consistently associated with depressive or anxiety symptoms within or across all tasks. Features associated with depressive symptoms were different for each task and there was only some degree of reliability of these features within tasks. In all machine learning models, predicted values were weakly associated with actual values. Some text tasks had lower levels of engagement and negative impacts on mood. Overall, the linguistic markers of depression and anxiety shifted in response to contextual factors and the nature of the text analysed. This trial was prospectively registered with the Australian New Zealand Clinical Trials Registry (date registered: 15 September 2021, ACTRN12621001248853).

## Introduction

Depression and anxiety are common mental illnesses that negatively affect the health and wellbeing of millions worldwide, eroding individuals’ quality of life and productivity. Identifying depressive and anxiety symptoms among individuals and populations in an automated and wide scale manner may allow for timely intervention at a population level, triaging risk, and preventing potential escalation into severe disorders and suicidality. Digital phenotyping is the emerging field of science that mines the digital data generated by individuals in the daily course of their lives to uncover “objective, quantifiable characteristics of illness that can be measured accurately and reproducibly”^1^. There has been a rapid increase in the number of research studies attempting to use this approach to understand various mental health states and the need for intervention among populations and individuals^2,3^. Automated linguistic analysis of individuals’ text data may be an advantageous method of digital phenotyping because individuals generate large volumes of text data daily, and these can be readily collected and analysed at scale using low-cost real time software^4,5^. By segmenting individuals’ text data into linguistic features that can be observed and quantified, linguistic analysis has enabled researchers to rapidly explore the relationships between linguistic expression and mental illness.

To date, several studies across psychology and computer science have suggested that linguistic features expressed in different forms of individuals’ text data may provide markers of the cognitive, emotional, and social deficits of depression and anxiety^6,7^. It has been hypothesised by many researchers that mental illness significantly alters one’s linguistic style whereby the words used by individuals may indicate the reduced positive emotion, ruminative self-preoccupation^8^, low levels of motivation and pleasure, and difficulty foreseeing positive future events that characterise depressive and anxious states^9–12^. Indeed, a meta-analysis of 21 studies of individuals’ written and spoken communications including personal essays, social media posts and clinician interviews, found that higher depressive symptoms were marked by the increased use of first-person singular pronouns, irrespective of gender or other demographics^13^. A second meta-analysis of 26 studies of individuals’ linguistic expression in varied writing tasks found that individuals with depressive symptoms were distinguished from non-depressed individuals by small to moderate differences in first-person singular pronouns use, negative emotion word use and positive emotion word use^14^. Another systematic review of 25 studies on the linguistic features of depression also found that first-person singular pronouns and negative emotion words were significantly associated with depressive symptoms, although the strength and magnitude of the results varied^15^. However, several studies attempting to identify linguistic markers of depression and anxiety have had conflicting findings^16–18^. This is likely influenced by differences in the text forms analysed and variations in methodology such as clinical versus community samples. Furthermore, the strength of the associations between linguistic features and symptoms are consistently weak suggesting limited utility for prediction. While collectively the studies suggest that there may be linguistic markers of mental illness, the validity, reliability and overall utility of this approach remains in its infancy.

There is also increasing evidence of a task effect whereby contextual factors (i.e., the purposes and conditions under which text data is created) may influence the strength and direction of the relationships between linguistic features and mental health symptoms. For example, Havigerova^19^ found that linguistic features expressed by individuals in a fictional letter to a friend inviting them on a holiday had greater predictive utility for depression than the linguistic features expressed in fictional formal cover letters, complaints, or apology letters. Similarly, Minori^20^ and Cuteri^21^ found that written personal reflections of a complex picture was the most useful text task for generating valid linguistic markers of depression when compared to written self-reflection tasks about one’s self or one’s friends. In contrast, Kazmierczak et al.^22^ found that depressed individuals consistently used the same types of language and word choices regardless of what they were describing, suggesting that some aspects of linguistic expression may be stable. However, these past studies have not accounted for the potential intervention effects of expressive writing tasks on participants’ mood^23^ or their levels of engagement. This is pertinent to the study of depression, given that this illness is characterised by low levels of motivation, biased processing of emotional stimulus, enhanced recall of negative emotional experiences, and difficulty foreseeing positive future events^9–12^. It remains unclear whether mental health symptoms have caused variations in linguistic expression, the task itself, or interaction effects, as most prior studies have not controlled for these factors.

With the growth of smartphone and digital-mediated communications, researchers have examined the utility of organic text data such as SMS and social media posts for generating markers of mental illness. When examining SMS text data, Stamatis et al.^24^, found that individuals with higher depressive symptoms used significantly fewer words related to anticipation, trust, social processes, and affiliation whereas the inverse use of words was found for high anxiety symptoms. Also in SMS data, Meyerhoff et al.^25^ found that individuals with higher depressive symptoms used significantly more first-person singular pronouns, filler words, sexual words, anger and negative emotion words, but only in their SMS with close contacts. Several studies have revealed potential linguistic markers of depression in individuals’ social media posts^26–31^ with several supervised and unsupervised machine learning models showing promise for detecting depressive symptoms^7^. Notably however, Waterloo et al.^32^ found that different digital communication platforms (e.g., WhatsApp, Facebook, Twitter and Instagram) elicited significant variations in an individual’s linguistic expression. This is consistent with Tlachac and Rundensteiner^33^, who found that that individuals’ SMS messages were more useful for detecting depression than their Twitter posts. Similarly, Liu^34^ also found significant cross-platform differences in linguistic expression, such that a Facebook-derived model of linguistic predictors of depression performed poorly when applied to SMS data from the same individuals. Smirnov^35^ also found that the number and significance of linguistics markers in social media messages and personal essays about oneself and their relations varied according to depression status (i.e., clinical depression diagnosis versus high depressive symptomatology) and text type. Based on their results, Smirnov and colleagues also posited that the linguistic markers of depression were most likely influenced by the text topic and the conditions under which the text was written, rather than mental health status alone.

Overall, variations in the types of text data, the number of linguistic features examined, and the analytical approaches used have made it difficult to assess the validity and reliability of linguistic markers of mental illness. Past studies have been largely observational, collecting text data from a single timepoint and source. In studies collecting and combining several types of text data, there has been little attempt to control for order effects, whereby the completion of certain study activities such as mental health surveys or negatively-anchored writing tasks may prime individuals to respond in particular ways. Many studies have not accounted for the varied contextual factors such as the individual^19^, the communication modality^36^, the audience domain, the task stimuli, or the study conditions, that may influence the emergence of linguistic markers of mental illness. Furthermore, the rapid changes in digital communication patterns among individuals as evidenced by generational shifts to short-form abbreviated messages and image-based content^37,38^ combined with the expansion of internet security measures to protect individuals’ data privacy, has made the automated extraction and mining of digital data by third parties increasingly difficult^39^. As digital phenotyping gains popularity and data collection becomes more invasive, it is essential that researchers have a strong scientific and ethical rationale for extracting and analysing of large volumes of personal text data. A clearer understanding of the potential utility of text data for providing meaningful insight into the health and wellbeing of individuals will help to ensure this.

Using a randomised cross over experimental design to control for potential order and task effects, this trial aimed to examine the validity and reliability of linguistic features of symptoms of depression and anxiety in symptomatic adults by comparing several text types previously found to generate significant markers. This study controlled for potential reverse-causation and interaction effects by separating out the text tasks, randomising delivery and collecting multiple samples over time. Furthermore, this study explored whether markers found at the task-level remained significant when all text data was combined into one corpus. Using machine learning models, this study aimed to determine which linguistic features were the most strongly associated with depressive and anxiety symptoms. This study also examined the acceptability of the text tasks and the effects of these on participants’ mood and engagement in this type of digital phenotyping. Based on prior findings, it was hypothesised that several linguistic features would be significantly associated with symptoms of depression and anxiety but there would be variability in the significance of these markers depending on the task type. Consequently, it was hypothesised that markers found to be significant at the task level may not retain their significance when all text data was combined. It was also hypothesised that text tasks with negative sentiment would negatively impact participants’ mood and be less engaging to complete. The findings of this investigation will help to determine the validity and reliability of linguistic markers of depression and anxiety and the acceptability of collecting text data for this approach.

## Methods

### Design

This study utilised a 9-week randomised crossover design to examine the utility of eight different types of text data of varying frequencies and emotional valance, for generating linguistic markers of depression and anxiety. The study took place between October 2021 and December 2021 and was conducted online. Ethics approval was obtained from the University of New South Wales Human Research Ethics Committee (HC210397), and the trial was prospectively registered with the Australian New Zealand Clinical Trials Registry (date registered: 15 September 2021, ACTRN12621001248853). This report concords with the CONSORT for randomised crossover design^40^.

Outlined in Table 1, this trial employed four unique sequences of text tasks to control for potential order effects. Participants were randomly allocated to one of the four sequences upon completion of baseline. Four of the text tasks (SMS, social media posts, emotion diaries, expressive essays) were repeated by participants up to 5 times throughout an intervention period (referred to from here on in as ‘repetitive tasks’, i.e. Tasks ABCD) to ensure the required volume of text was collected and to enable repeated analyses. The repetitive tasks were administered according to a set frequency within the allocated sequence, whereas the once-off tasks (i.e., Tasks VWXY) were completed in the same order for all participants. In total, participants were asked to complete 19 tasks, which are outlined in the procedure.

**Table 1:** The text task allocations in each of the four sequences in the randomised cross-over trial design

Sequence (Task order)	Day 1	Period 1	Day 14	Period 2	Day 28	Period 3	Day 42	Period 4	Day 56
1 (AVBWCXDY)	Nil	A	V	B	W	C	X	D	Y
2 (BVCWDXAY)	Nil	B	V	C	W	D	X	A	Y
3 (CVDWAXBY)	Nil	C	V	D	W	A	X	B	Y
4 (DVAWBXCY)	Nil	D	V	A	W	B	X	C	Y

Task descriptions: A = SMS retrieval, B=Social media post, C=Emotion diary, D=Expressive writing, V=Personal description, W=Friends description, X=Reflection of a complex image, Y=Holidaying letter. Each intervention period was 14 days. The total study duration was 63 days including 7 days completion time for final survey.

**Fig. 1:**
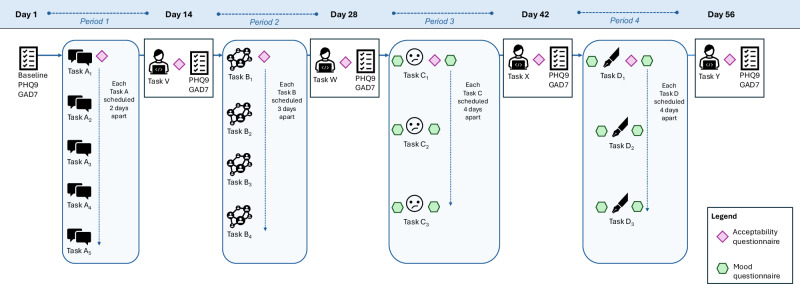
Example of study timeline and schedule of activities for participants allocated to sequence one. The blue shading lane at the top of the figure indicates the study timeline for the entire trial period, beginning on day 1 and ending on day 56. The blue shaded rectangles with curved edges indicate when the grouped (i.e. repetitive) text tasks were administered to participants allocated to sequence one. The square-edge rectangles indicate when the once-off text tasks were administered fortnightly, prior to the completion of the acceptability questionnaires (as indicated by the purple diamonds) and mental health questionnaires (containing the PHQ-9 and GAD-7), with the latter indicated by the checklist icons. The green hexagons indicate the administration of the mood monitoring questionnaires.

After each text task was completed for the first time, participants then immediately completed an acceptability questionnaire. Participants were also asked to complete five fortnightly mental health symptom questionnaires on days 1 (baseline), 14, 28, 42, and 56 (study endpoint). The mental health symptom questionnaires were completed immediately after the once-off text tasks to control for any carry-over effects. A ‘wash out’ period (minimum of 3 days) was implemented between the fortnightly mental health symptom questionnaires and the repetitive text tasks to further reduce any carryover effects of the mental health questionnaires. Figure 1 provides the study timeline and schedule of activities for participants allocated to sequence 1.

### Participants and sample size

As the study aimed to examine the relationship between linguistic features and depressive/anxiety symptoms, participants were required to report at least moderate symptoms of depression or anxiety (i.e., total score ≥ 10 on the Patient Health Questionnaire-9 or the Generalized Anxiety Disorder scale at screening), be at least 18 years of age, located in Australia, and have an active mobile phone number and email address. Participants who reported a suicide attempt in the past six months or who reported that they were currently experiencing extreme and unmanageable emotional distress at screening were excluded. The target sample size was set at a minimum of 140 participants, based on detecting moderate correlations (*r* = 0.3–0.5) between linguistic features and mental health symptoms, and a 40% attrition rate.

### Recruitment and consent

This study utilised a paid social media campaign on Facebook and Instagram. Study adverts also featured in the Black Dog Institute’s e-newsletters, website, and social media channels (Instagram and Facebook). Individuals who clicked on the study adverts were redirected to the study website where they were able to complete the eligibility screener. Eligible participants provided their consent to participate via an online Participant Information Sheet and Consent Form.

### Procedure

Tasks are presented in Table 2.

**Table 2:** Descriptions of the text tasks, including task labels, task type, task frequency and task instructions, used in the current study

Task label	Task type	Task frequency	Task instruction
A	SMS retrieval^33^	Every two days, participants were asked to retrieve their most recent SMS. Task was repeated 5 times in the intervention period.	Please copy and paste a private message that you have sent to any close friend of yours in the past two weeks. Pick a message that has the most amount of written text and reflects a personal relationship. Don’t worry about what the message says (for example, swear words)! For this activity, we are interested in how you write, not what you write.
B	Social media post^66^	Every three days, participants were asked to make a fictional post on social media. Task was repeated 4 times in the intervention period.	Tell us, what’s on your mind right now? We’d like you to imagine that you are writing a post about how you are feeling right now to share on a social media platform (e.g., Facebook, Instagram, Twitter). Imagine that people you know will read it. We understand that many of you may not use social media or wouldn’t normally share a post like this. We are interested to see what you would write if you did. For this task, the maximum character limit is set at 5000 to match the limit of common social media platforms.
C	Emotion diary^17^	Every four days, participants were asked to write about an event that impacted their mood. Task was repeated 3 times in the intervention period.	Please write about an event today that affected your mood. Please write about your feelings related to this event. Are there any words (e.g. compliment, encouragement, enlightenment) that you’d like to tell yourself?
D	Expressive essay^23,67–71^	Every four days, participants were asked to write about their deepest thoughts and feelings. This task was repeated 3 times in the intervention period.	We want you to write about your deepest thoughts and feelings about an extremely important emotional issue that has affected you and your life. We’d like you to really let go and explore your deepest emotions and thoughts. Think about what the issue or experience means for you, your relationships with others, the past, present, your future, who you would like to be, and who you are now.
V	Personal writing task (description of self)^20,21^	Scheduled once, on day 14, completed prior to mental health questionnaire.	Imagine you are meeting someone for the first time. How would you describe yourself? Tell us about you! Include things like your personality traits, your values, hobbies and interests, what you look like, your family, your work, favourite activities etc.
W	Neutral writing task (Friends)^20,21^	Scheduled once on day 28, completed prior to mental health questionnaire.	Tell us about your friends. How do you usually spend time together? Tell us about the things you and your friends enjoy. You can also share any challenges you may be having with your friends right now.
X	Narrative imagery task – Description of a complex picture^20,21^	Scheduled once on day 42, completed prior to mental health questionnaire.	Tell us in your own words, what you think is happening in this image? (Cookie Theft from the Boston Diagnostic Aphasia Examination Battery).
Y	Holidaying letter to a friend^19^	Scheduled once on day 56, completed prior to mental health questionnaire.	Imagine you are enjoying your time on an amazing holiday. Everything is going well, and you are having fun doing all your favourite things. We want you to write a letter to your friend to convince him/her to come and enjoy this fun time with you.

Participants received an initial email and SMS notification to complete each task, followed by two reminders. Text tasks and surveys were only open for completion within the allocated two-week intervention period. There was no word limit for any of the tasks (except for Task B so that it replicated a social media platform). Participants were reminded not to worry about spelling, sentence structure or grammar, to just write freely as instructed. Participants could opt out of any task at any time and had the option to report “I feel too distressed to complete this task today”. To reduce attrition, the reimbursement for the mental health surveys was 10AUD at baseline and increased by 5AUD every second fortnight. Participants also received an additional 5AUD for each of the attempted one-off tasks. The maximum reimbursement received was 95AUD.

### Measures

#### Depressive symptoms

The Patient Health Questionnaire-9 (PHQ-9) is a widely used 9-item, validated self-report questionnaire that assesses the severity of depressive symptoms over the past two weeks. Each item is scored on a scale ranging from 0 (not at all) to 3 (nearly every day). Item scores are summed to obtain a total score, which can range from 0 to 27. Higher scores indicate higher levels of depressive symptoms. Total scores can be used to indicate ‘nil to mild’ symptoms (0-9), ‘moderate’ symptoms (10-14), and ‘moderately severe’ to ‘severe’ symptoms (15 + ). In the current study, the Cronbach’s alpha for the PHQ-9 was 0.72.

#### Anxiety symptoms

The Generalized Anxiety Disorder-7 (GAD-7) is a widely used, 7-item validated self-report questionnaire that assesses the presence of generalized anxiety symptoms in the past two weeks. Each item is scored on a scale ranging from 0 (not at all) to 3 (nearly every day). Item scores are summed to obtain a total score, which can range from 0 to 21. Higher scores indicate a higher levels of anxiety symptoms. Total scores can be used to indicate ‘nil to mild’ symptoms (0-9), ‘moderate’ symptoms (10-14), and ‘moderately severe’ to ‘severe’ symptoms (15 + ). In the current study, the Cronbach’s alpha for the GAD-7 was 0.83.

#### Demographics

At baseline, participants reported their age, gender identity, educational attainment, relationship status, paid employment, whether they were born in Australia, whether they identified as being First Nations peoples (Aboriginal and/or Torres Strait Islander), gender- and/or sexuality-diverse (Lesbian, Gay, Bisexual, Transgender, Queer, Intersex, and/or other sexuality and gender diversity; LGBTQI + ), and the Internet device that they completed the baseline survey on. Participants were asked to report what language they usually spoke at home and how often they needed help from someone to complete forms or read written material (e.g., brochures, newspapers), answered on a 5-point Likert scale ranging from never (0) to always (4).

#### Clinical characteristics

At baseline, participants were asked whether they had ever received a formal diagnosis of depression, anxiety, or other mental illness from a medical practitioner or mental health professional, the duration of mental illness (age when formally diagnosed, age of onset), and whether they were currently taking any antidepressant or other pharmacological medication for their mental illness, as prescribed by a medical practitioner.

#### Mood monitoring

The 6-item short form of the Multidimensional Mood Questionnaire (MDMQ)^41^ was administered immediately before and after the negatively-anchored expressive tasks (i.e., Task C and D, total of 6 times) to explore the impact of these on participants’ mood. Using a 7-point Likert scale, participants were asked to indicate how they felt in this very moment, in relation to six dimensions of mood: fatigue, content, agitation, energy, unwell, and relaxation. The scale has demonstrated the ability to differentiate synchronized mood states and changes within individuals over time^41^. No reverse scoring was implemented in this exploratory analysis.

#### Acceptability

Participants were asked to complete a short 3-item acceptability questionnaire upon first completion of the tasks. The first item “How easy or difficult was this task?” was answered using a 7-point Likert scale from very difficult (1) to very easy (7). The second item “How interesting was this task?” was answered using a Likert scale not at all interesting (1) to very interesting (7). The final item “How willing are you to complete this task again?” was answered using a 7-point Likert scale from not at all willing (1) to very willing (7).

### Data collection and analysis

Data was collected using the Black Dog Institute Research Engine. Participants’ text data was pre-processed in Microsoft Excel and prepared according to the manual of the validated Linguistic Inquiry and Word Count (LIWC-22) tool^42^. Any entries containing the default distress response (i.e., “I feel too distressed to complete this task today”) were classified as “*Task attempted*” but “*Invalid for analysis – participant distressed*”. For task A (SMS) only, content was reviewed by a research assistant to ensure that participants had entered only their own SMS data, rather than SMS conversations with others. If conversations with others were identified, the text content that was not authored by the participant was removed for analysis. These responses were then coded as “*Valid for analysis – SMS edited*”. Participants who reported that they did not have suitable SMS data available (i.e., did not send SMS during that period) were also classified as task “attempted” but “*Invalid for analysis– no SMS sent”*. Participants who attempted to deidentify their SMS content by replacing personal names with [“XXX”] within the SMS had their responses classified as “*Valid – name edited*” and the [“XXX”] was replaced with a formal placeholder [name] and this was excluded from the LIWC-22 analysis. For Task A (SMS), emoticons and emojis remained unedited as LIWC-22 ignores these in the analysis. All other entry types (e.g. email addresses, URL addresses) were replaced according to the LIWC-22 manual, and a spell check was run on all responses with spelling errors amended.

The LIWC-22 tool was used to calculate the percentage of words within the text that reflected a predefined dictionary of 117 linguistic features. The LIWC-22 summary variables (analytical thinking, clout, authenticity, and emotional tone) were excluded from our analyses due to the lack of transparency on the dimensions of these variables, resulting in 113 linguistic features. Linguistic features were first extracted within each task, then averaged across tasks, while mental health scores were averaged across task timepoints in the combined analyses.

All analyses were performed using R 4.2.1^43^. Between-subject ANOVAs were used to compare differences in mental health outcomes across the four trial sequences at baseline. ANCOVAs were also used to control for the demographics age, gender, and education. Post-hoc tests were corrected for using Tukey’s HSD method. The four sequences were then combined into one sample for analysis given no significant differences in symptoms across sequences. The sequences were collapsed into one sample because at baseline, there were no significant differences in depressive (*F*(3,214) = 0.676, *P* = 0.568) or anxiety symptoms (*F*(3,214) = 0.714, *P* = 0.545) between the sequences and the results remain unchanged when age, sex and education were accounted for (*P* = 0.568–0.545). There were some significant changes over time for participants’ depressive symptoms (*F*(4,771) = 5.289, *P* < .001) but no significant differences across sequences (*F*(3,215) = 0.158, *P* = 0.924) and no significant interaction between time and sequence (*F*(12,771) = 1.393, *P* = 0.163). There was also no change over time in participants’ anxiety symptoms (*F*(4,769) = 1.224, *P* = 0.299), no differences across the sequences (*F*(3,213) = 0.802, *P* = 0.494) and no significant interaction between time and sequence (*F*(12,769) = 0.576, *P* = 0.862).

Correlation plots were created using the ‘ggplot2’ package in R^44^. Nested cross-validation machine learning models were developed using the “nestedcv” package in R^45^ and the ‘caret’ package in R^46^. Pearson correlations were used to test associations between linguistic features and mental health symptoms. The false discovery rate of *p*-values were adjusted to *q*-values using the Benjamani and Hochberg approach^47^. Models were run with and without outliers for sensitivity testing.

When testing the associations between participants’ linguistic features and mental health symptoms, common machine learning models including elastic net models, random forest, and support vector machines were used to test whether linguistic features predicted mental health symptoms. Models were evaluated using nested cross-validation. Nested cross-validation was selected as it maximises the use of the whole dataset for testing overall performance while maintaining the split between training and testing samples, and provides the next best estimate of real-world predictive performance when external datasets are not available^45,48,49^. For the nested cross-validation, data were first split into 10 outer and 10 inner cross-validation folds stratified by mental health scores to ensure representative distributions of participants in each fold. Inner folds were used to evaluate the performance of the hyperparameters. For each outer fold, the hyperparameter configuration with the lowest root mean squared error (RMSE) across the inner folds was selected, retrained on all inner-fold data of that outer fold, and used to predict on the held-out outer fold data. These predictions on held-out data had not been seen during training and were used to compute the final performance reported in the results. Models were also developed with recursive feature elimination embedded within the outer CV loops to test whether this improved performance.

We optimized the hyperparameters of the Elastic Net model, which combines the properties of both ridge and lasso regression. The parameters tuned were the mixing parameter α and the regularization parameter λ. The mixing parameter α determines the balance between lasso (L1) and ridge (L2) regularization, ranging from 0 (ridge-only) to 1 (lasso-only). We varied α linearly, testing 11 evenly spaced values from 0 to 1. The regularization parameter λ, which controls the overall strength of the regularization, was explored over a logarithmic scale, with values ranging from 2^−15^ to 2^5^. This approach allows for a detailed investigation into the effects of both mild and strong regularization on model performance.

For the Random Forest modelling, we optimized the hyperparameter *mtry*, which specifies the number of features randomly sampled as candidates at each split in the construction of the trees within the Random Forest model. The parameter *mtry* controls the diversity of the trees and thus the bias-variance trade off of the overall model. The values of *mtry* investigated were chosen to encompass a broad range, starting from 1 up to 20, and including specific larger values at 30, 40, 50, and 70. This selection was designed to evaluate the model’s performance across a diverse set of scenarios, from using very few features at each split, which tends to produce more biased trees, to using many features, which can reduce bias but increase variance and computational demand.

We optimized the hyperparameters of the Support Vector Machine (SVM) using both linear and non-linear configurations to ensure robust model performance. For the linear SVM, we tuned the regularization parameter *C*, while for the non-linear SVM with a radial basis function (RBF) kernel, we tuned both *C* and the kernel coefficient γ. For *C*, the values tested ranged from 2^−15^ to 2^5^, allowing exploration from very high regularization (to prevent overfitting) to very low regularization (to capture more complex patterns in the data). Similarly, γ was varied across the same range, from to 2^−15^ 2^5^, to thoroughly investigate the influence of kernel scale on the decision boundary smoothness and model flexibility.

Performance of the models was assessed by inspecting the RMSE, *r*^2^, the mean absolute error (MAE), and by correlating the predicted and observed values. Models were also developed using demographics (age, gender, education) and clinical characteristics (diagnosis age, diagnosis onset, medication status) to determine whether the inclusion of these could improve model performance. One-hot encoding was used for categorical variables and those with more than two levels were dichotomized, including education (secondary schooling versus university education), and gender (male versus female; for a full list of variables, see Supplementary Material Table S1). Variable stability plots were generated to visualize how often predictors were selected in each outer fold and final model selection. Feature importance for final models fit to the whole dataset were assessed using Shapley Additive Explanation (SHAP) values, which quantify the contribution of each feature to the predictions of a machine learning model for each participant. A higher absolute SHAP value implies a stronger impact of that feature on the model’s output. Bee swarm plots were also used to visualize SHAP values and inspect the distribution of the effect across participants.

Mixed linear models were used to test whether the expressive text tasks impacted mood and the acceptability scores. Models included mood scores (MDMQ) and acceptability scores as the dependent variables, subject as the random effect, and time, sequence group, and the interaction between time and sequence group as the fixed effects. For the impacts of the text tasks on mood, planned contrasts examined the changes in mood between completions 1 and 2, completions 3 and 4, completions 5 and 6 only. The method of Kenward and Roger was used to estimate the degrees of freedom for all these tests.

## Results

### Participants

**Fig. 2:**
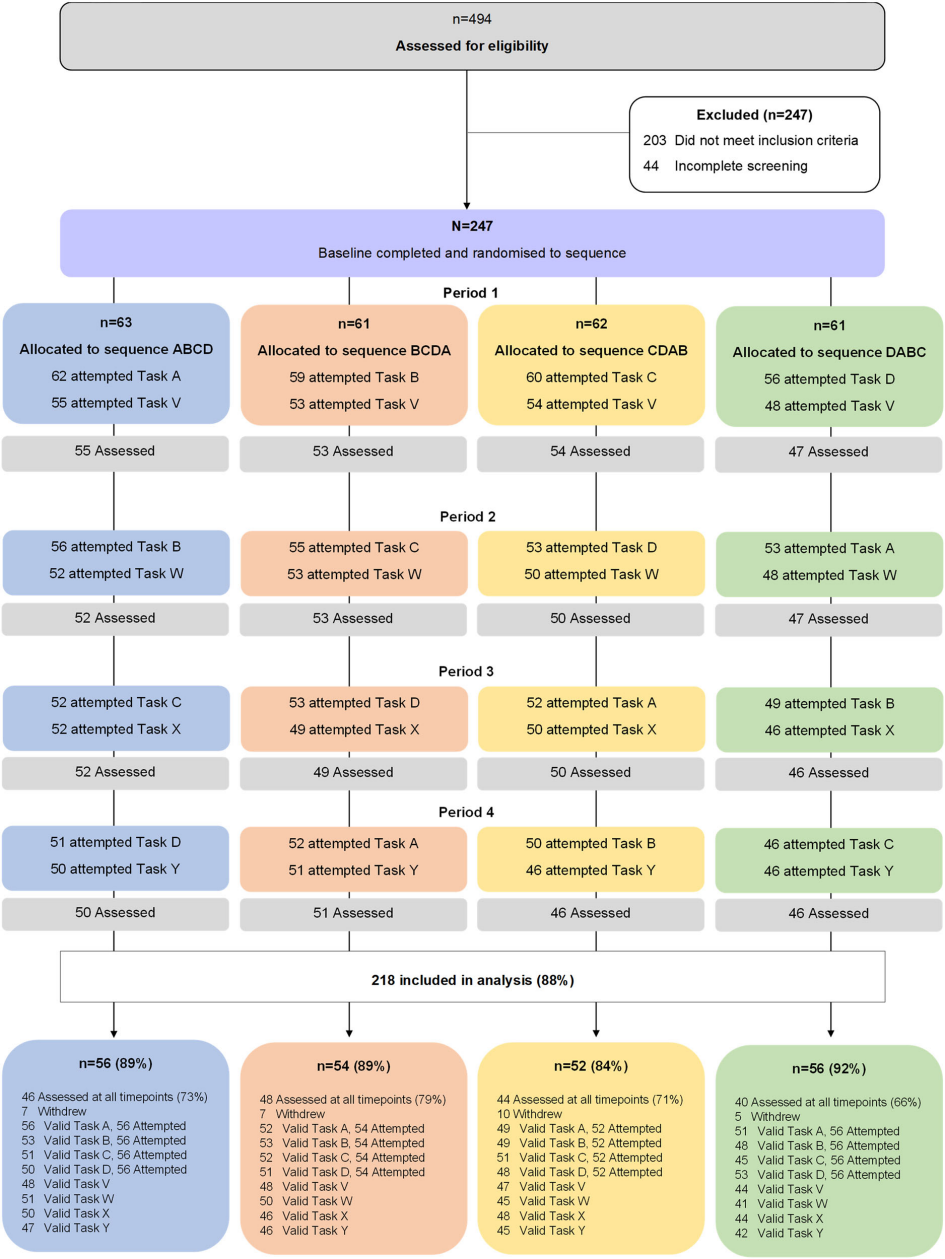
CONSORT study flow diagram. ‘Period’=14 days (i.e., fortnight). ‘Attempted’ defined as participant attempted the task. ‘Assessed’ defined as participant completing the PHQ-9 for that period. ‘Valid’ defined as text response was confirmed eligible for analysis.

A total of 494 adults were assessed for study eligibility. Of these, 247 consented, completed baseline and were randomised. A total of 29 participants actively withdrew after randomisation (see Fig. 2 for study flow).

Table 3 outlines the baseline characteristics of the final sample (*N* = 218). The majority were female (74.8%), Australian born (80.3%), married or partnered (52.8%), in paid employment (74.8%) with a mean age of 39.33 years (SD: 12.37, range: 18–69 years) and English as the main language spoken at home (90.8%). The majority (85.3%) reported that they never required any literacy support. Nearly all participants reported that they had received a formal diagnosis of a mental illness at baseline (92.7%, *n* = 202/218) and over half were taking prescribed medication for a mental illness (59.6%, *n* = 130/218).

**Table 3:** Baseline characteristics of sample (*N* = 218)

	Total sample	
	M	SD
Age (years)	39.33	12.37
Depressive symptoms (PHQ9)	13.50	4.13
Anxiety symptoms (GAD7)	10.83	4.51
	**N**	**%**
Female	163	74.8
University education	105	48.2
Married/Partnered	115	52.8
In paid employment	163	74.8
Born in Australia	175	80.3
First Nations	5	2.3
LGBTQI+	45	20.6
English only	198	90.8
Literacy support (Never)	186	85.3
Formally diagnosed mental illness	202	92.7
Age of onset	19.91	10.83
Age of diagnosis	26.94	10.93
Taking prescribed medication	130	59.6
Likely case of Major Depressive Disorder	193	88.5
Likely case of Generalized Anxiety Disorder	134	61.5

Likely case of Major Depressive Disorder (MDD) determined by a PHQ9 score ≥ 10; Likely case of Generalised Anxiety Disorder (GAD) determined by a GAD7 score ≥ 10.

### Completion of text tasks

On average, participants attempted 16.41 out of the 19 tasks (SD: 4.87, range: 0–19). The majority completed their first text task on a mobile device (88.5%). Participants in sequence 4 (M: 14.84, SD: 5.99) attempted significantly fewer tasks compared to those in sequence 2 (M: 17.43, SD: 3.86; t = 2.843, *P* < 0.05). No other differences in completion rates were found. Table 4 presents the word counts, character counts, and proportion of dictionary words within tasks.

**Table 4:** Word counts, character counts, and proportion of dictionary words within tasks per task and per participant

Task	Task - word count	Task - character count	Task - proportion of dictionary words	Participant corpus - word count	Participant corpus - character count	Participant corpus -proportion of dictionary words
	M (SD)	M (SD)	M (SD)	M (SD)	M (SD)	M (SD)
A. SMS retrieval	64.88 (76.88)	336.17 (400.13)	93.01 (5.10)	280.88 (258.14)	1455.37 (1351.33)	93.07 (2.96)
B. Social media posts	62.18 (63.30)	328.10 (331.63)	94.09 (6.04)	219.81 (180.47)	1159.87 (946.47)	94.13 (3.66)
C. Emotion diaries	94.31 (75.62)	488.09 (394.78)	95.43 (3.59)	259.70 (189.24)	1344.03 (988.37)	95.45 (2.37)
D. Expressive writing	223.01 (198.22)	1164.18 (1034.49)	95.53 (2.65)	527.50 (479.72)	2753.89 (2506.60)	95.54 (2.04)
V. Personal description	-	-	-	197.34 (144.06)	1041.64 (761.26)	92.90 (3.42)
W. Friend description	-	-	-	167.02 (142.39)	870.65 (742.69)	95.59 (2.50)
X. Reflection of a complex image	-	-	-	79.44 (53.9)	430.74 (291.26)	92.26 (4.79)
Y. Holidaying letter	-	-	-	117.21 (72.00)	603.29 (373.16)	92.64 (3.96)

### Within-task associations between mental health symptoms and linguistic features

Significant correlations between mental health symptoms and the 113 linguistic features were found in three of the eight tasks. In the Task B corpus (i.e., combined social media posts), higher depressive symptoms were associated with greater expressions of “power” words (*r* = 0.27, *q* = 0.010), greater expressions of “sadness” words (*r* = 0.26, *q* = 0.010) and reduced first-person plural pronouns (*r* = −0.26, *q* = 0.010). See Supplementary Material for plots. As shown in Table 5, these features were not found to be consistently associated with depressive symptoms when the Task B completions were examined separately.

**Table 5:** Associations between the significant task-level linguistic features (power words, sadness words, first-person plural pronouns) and depressive symptoms within each Task B completion

Task - Completion	Power words (*r*)	Sadness words (*r*)	First-person plural pronouns (*r*)	*q*-range
Task B-1	0.09	0.18**	−0.17*	0.546–0.929
Task B-2	0.22**	0.08	−0.17*	0.272–1.0
Task B-3	0.05	0.21**	−0.14	0.229–0.986
Task B-4	0.17*	0.05	−0.09	0.551–1.0

*Unadjusted *P* < 0.05, **Unadjusted *P* < 0.02.

In Task V (personal description), higher levels of depressive symptoms were associated with greater use of “cognition” words (*r* = 0.25, *q* = 0.032), greater use of “tentative” words (*r* = 0.25, *q* = 0.032), and higher levels of “negative tone” (*r* = 0.23, *q* = 0.045). When outliers were removed, “tentative” words (*r* = 0.19, *q* = 0.175) and “negative tone” (*r* = 0.17, *q* = 0.195) were non-significant. In Task W (description of friends), higher levels of depressive symptoms were associated with greater expressions of “want” words (*r* = 0.27, *q* = 0.028). See Supplementary Material for plots. No other significant correlations were found.

Shown in Table 6, the within-task machine learning models predicted up to 8% (Task B corpus) of the variance in depressive symptoms and up to 4% (Task B corpus) of the variance in anxiety symptoms. The best-performing models varied depending on the task. The models that included the linguistic features from the Task B corpus together with clinical and demographic characteristics accounted for the greatest level of variance in symptoms. The observed and predicted values from the outer cross-validation folds were significantly correlated for all depression models whereas this pattern was only found for Tasks A, B, V, and X in the anxiety models. Across the models, depression scores were predicted with a mean absolute error of 4.13–4.77 points on the PHQ-9 scale, and anxiety scores were predicted with a MAE of 4.14–4.51 points on the GAD-7 scale. The importance and directionality of linguistic features in the models varied greatly between tasks, with clinical and demographic features showing high importance in some models (see Supplementary Material).

**Table 6:** The best performing within-task machine learning models using linguistic features to predict depressive and anxiety symptoms

	Depressive symptoms (PHQ-9)								
	Model	RFE	Demographic and clinical features	*RMSE*	*r*^*2*^	*MAE*	*r*	*p*	Hyperparameters for final fitted model
Task A	SVM-NL	No	Yes	5.77	0.05	4.69	0.22	0.005	C = 1 Σ = 0.001
Task B	Elastic Net	No	Yes	5.27	0.08	4.41	0.28	<0.001	α = 0.6 λ = 1
Task C	Random Forest	No	No	5.74	0.04	4.68	0.20	0.006	mtry = 50
Task D	SVM-NL	No	Yes	5.17	0.04	4.24	0.19	0.018	C = 2 Σ =0.008
Task V	Random Forest	No	No	5.15	0.03	4.13	0.18	0.012	mtry = 16
Task W	Elastic Net^	No	Yes	5.82	0.05	4.77	0.22	0.005	α = 0 λ = 16
Task X	Random Forest	Yes	Yes	5.66	0.05	4.50	0.22	0.005	mtry = 50
Task Y	Random Forest	No	Yes	5.62	0.07	4.61	0.26	<0.001	mtry = 8
	Anxiety symptoms (GAD-7)								
	Model	RFE	Demographic and clinical features	*RMSE*	*r*^*2*^	*MAE*	*r*	*p*	Hyperparameters for final fitted model
Task A	Random Forest	No	Yes	5.26	0.03	4.51	0.16	0.042	mtry = 1
Task B	SVM-L	No	Yes	5.03	0.04	4.22	0.21	0.007	C = 0.001
Task C	Random Forest	Yes	Yes	5.02	0.02	4.14	0.15	0.058	mtry = 1
Task D	Elastic Net	Yes	No	5.03	0.01	4.24	0.04	0.585	α = 0 λ = 2
Task V	Elastic Net	No	No	4.90	0.03	4.14	0.16	0.027	α = 0.5 λ = 1
Task W	SVM-L	No	Yes	5.29	0.01	4.46	0.09	0.257	C =0.001
Task X	SVM-L	Yes	Yes	5.02	0.03	4.25	0.18	0.022	C =0.016
Task Y	SVM-L	No	Yes	5.13	0.01	4.28	0.12	0.147	C =0.0005

Task descriptions: A = SMS retrieval, B=Social media post, C=Emotion diary, D=Expressive writing, V=Personal description, W=Friends description, X=Reflection of a complex image, Y=Holidaying letter. ^α = 0 represents ridge regression in the elastic net framework.

### Associations between mental health symptoms and linguistic features when all tasks were combined

As shown in Table 7, only two of the linguistic features found to be significantly associated with depressive symptoms within the tasks were found to be significant when texts from all the tasks were combined into one corpus. In this analysis, higher levels of depressive symptoms were associated with greater use of “cognition” words (*r* = 0.24, *q* = 0.024) and “cognitive process” words (*r* = 0.22, *q* = 0.027), lower levels of “positive tone” (*r* = −0.32, *q* < 0.001) and “positive emotion” (*r* = −0.21, *q* = 0.037), reduced first-person plural pronouns (*r* = −0.25, *q* = 0.016), and reduced “affiliation” words (*r* = −0.23, *q* = 0.024). Higher levels of anxiety symptoms were associated with greater use of “cognition words” only (*r* = 0.26, *q* = 0.022). See Supplementary Material for plots.

**Table 7:** Linguistic features significantly associated with depressive and anxiety symptoms within-tasks and when tasks were combined

Linguistic features associated with depressive symptoms (PHQ9) within-tasks	Task							
	A	B	C	D	V	W	X	Y
Cognition	**-**	**-**	**-**	**-**	*	**-**	**-**	**-**
First-person plural pronouns	**-**	*	**-**	**-**	**-**	**-**	**-**	**-**
Power	**-**	*	**-**	**-**	**-**	**-**	**-**	**-**
Sadness	**-**	*	**-**	**-**	**-**	**-**	**-**	**-**
Want	**-**	**-**	**-**	**-**	**-**	*	**-**	**-**
**Linguistic features associated with depressive symptoms (PHQ9) when combined**	**A**	**B**	**C**	**D**	**V**	**W**	**X**	**Y**
Affiliation	**-**	**-**	**-**	**-**	**-**	**-**	**-**	**-**
Cognition	**-**	**-**	**-**	**-**	*	**-**	**-**	**-**
Cognitive processes	**-**	**-**	**-**	**-**	**-**	**-**	**-**	**-**
First-person plural pronouns	**-**	*	**-**	**-**	**-**	**-**	**-**	**-**
Positive emotion	**-**	**-**	**-**	**-**	**-**	**-**	**-**	**-**
Positive tone	**-**	**-**	**-**	**-**	**-**	**-**	**-**	**-**
**Linguistic features associated with anxiety symptoms (GAD7) within-tasks**	**Task**							
	**A**	**B**	**C**	**D**	**V**	**W**	**X**	**Y**
None	**-**	**-**	**-**	**-**	**-**	**-**	**-**	**-**
**Linguistic features associated with anxiety symptoms (GAD7) when combined**	**A**	**B**	**C**	**D**	**V**	**W**	**X**	**Y**
Cognition	**-**	**-**	**-**	**-**	**-**	**-**	**-**	**-**

*Table note:* Linguistic features listed in left column are all those found to be associated with symptoms of depression (PHQ-9) and anxiety (GAD-7) at either the task level or when combined. * indicates the features with a significant *q* value for that task when examined with outliers removed. – indicates no significant *q* value for that task with outliers removed.

**Fig. 3:**
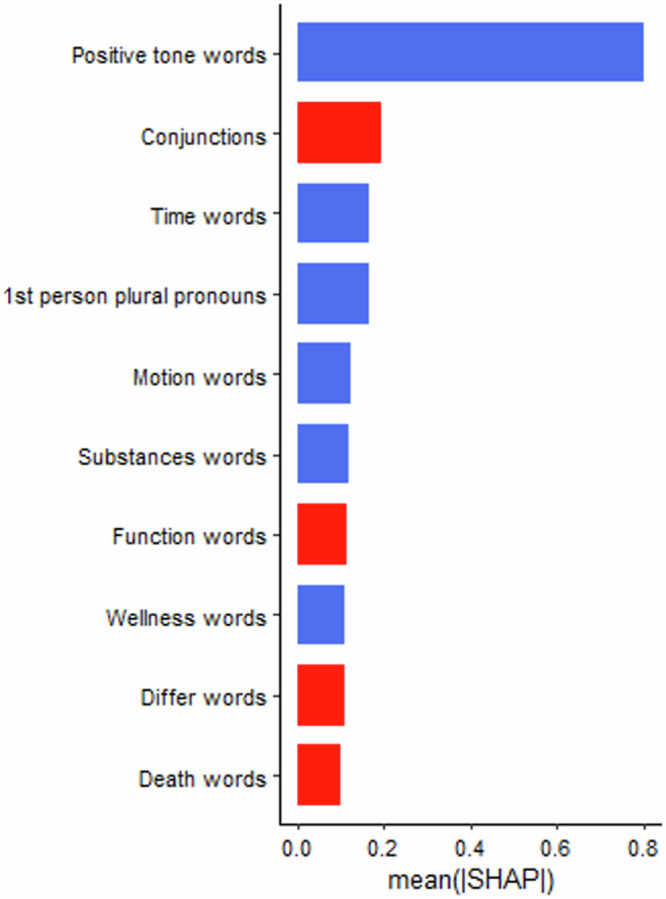
The importance of the linguistic features using the mean SHAP values from the best performing across-task machine learning models predicting depressive symptoms. SHAP values illustrate how the linguistic features contributed to the prediction of depressive symptoms in the machine learning models. In this figure, the linguistic features are ordered by the sum of SHAP values over all participants (i.e., mean), with features at the top of the plots being the most important in the model. The blue shading indicates the linguistic features with negative associations with depressive symptoms whereas the red shading indicates linguistic with positive associations with depressive symptoms.

**Fig. 4:**
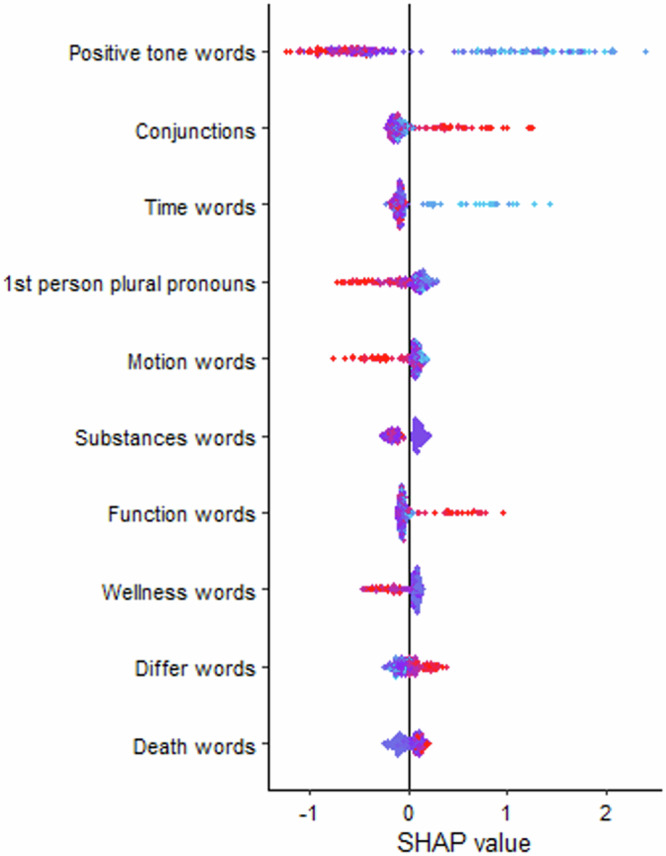
SHAP values for the linguistic features from the best performing across-task machine learning models predicting depressive symptoms. This figure displays the SHAP values. The position of each dot on the x-axis represents the impact of that linguistic feature on the machine learning model’s output for predicting depressive symptoms. Linguistic features that increase prediction value are shown in red (i.e., of higher value); those decrease the prediction value are in blue (i.e., of lower value).

**Fig. 5:**
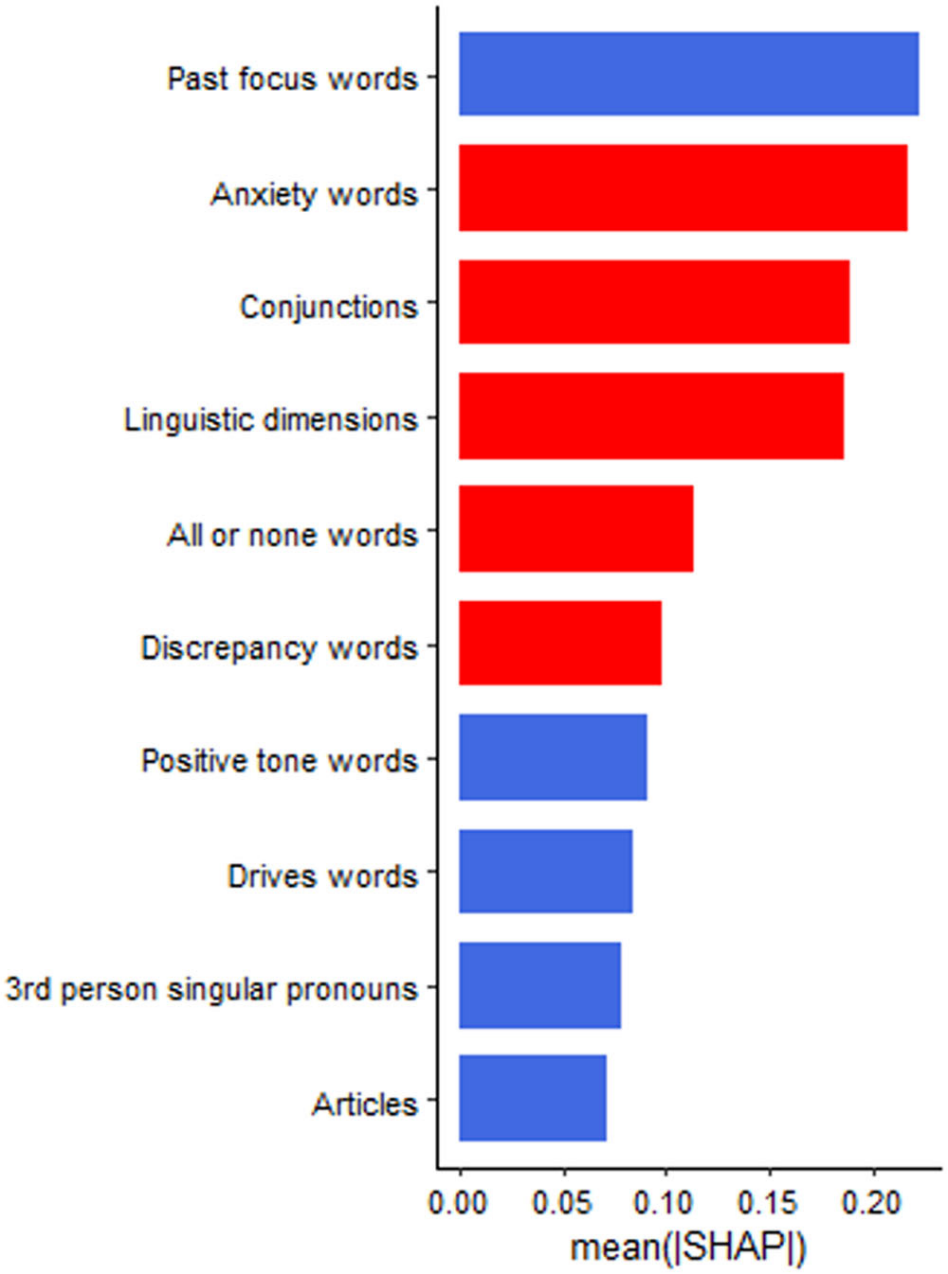
The importance of the linguistic features using the mean SHAP values from the best performing across-task machine learning models predicting anxiety symptoms. SHAP values illustrate how the linguistic features contributed to the prediction of anxiety symptoms in the machine learning models. In this figure, the linguistic features are ordered by the sum of SHAP values over all participants (i.e., mean), with features at the top of the plots being the most important in the model. The blue shading indicates the linguistic features with negative associations with anxiety symptoms whereas the red shading indicates linguistic with positive associations with anxiety symptoms.

**Fig. 6:**
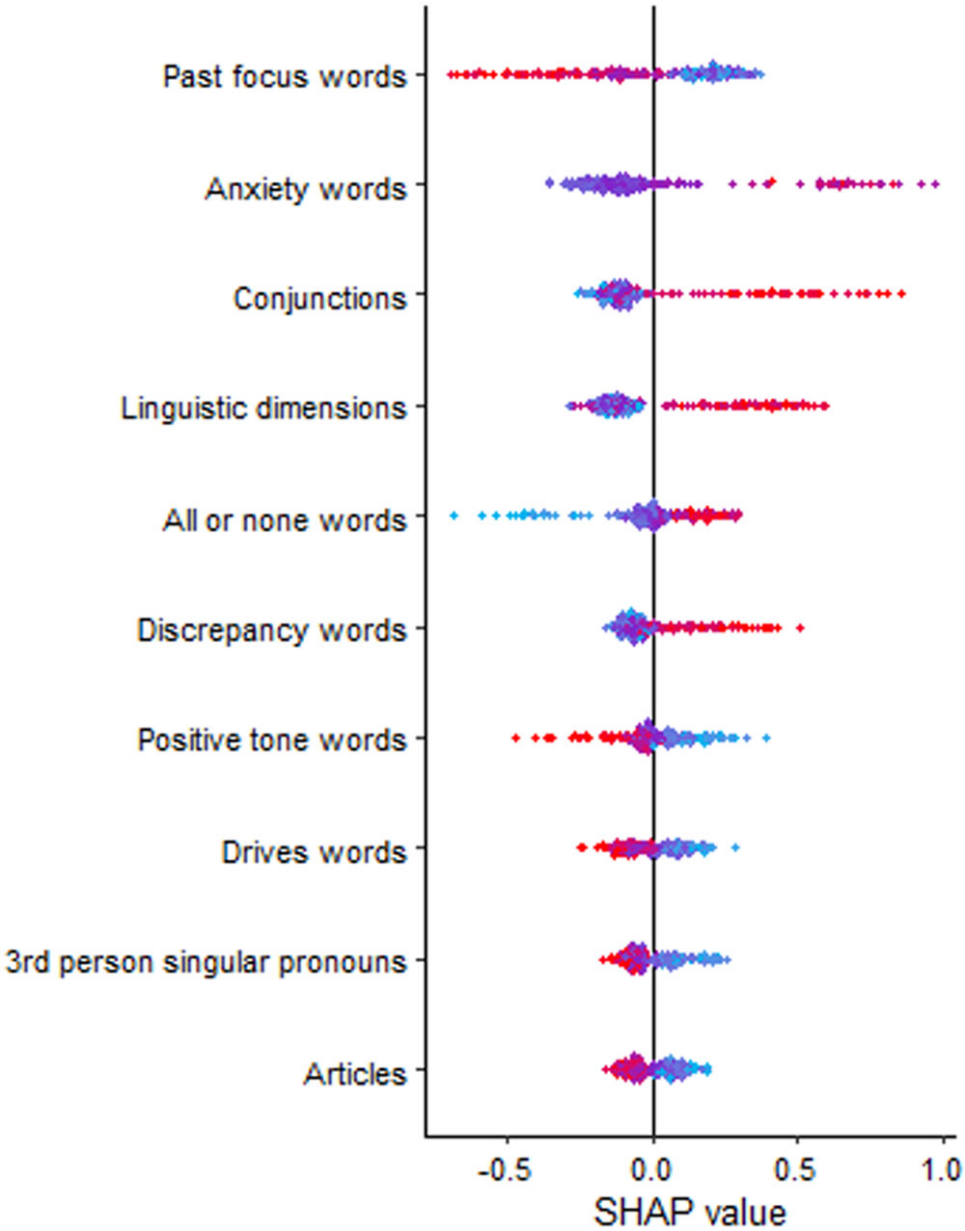
SHAP values for the linguistic features from the best performing across-task machine learning models predicting anxiety symptoms. This figure displays the SHAP values. The position of each dot on the x-axis represents the impact of that linguistic feature on the machine learning model’s output for predicting anxiety symptoms. Linguistic features that increase the prediction value are shown in red (i.e., of higher value); those decrease the prediction value are in blue (i.e., of lower value).

Shown in Table 8, random forest models using only linguistic features were the best performing models, predicting 8% of the variance in depressive symptoms and 3% of the variance in anxiety symptoms. The observed and predicted values from the outer cross-validation folds were significantly correlated for both the depression model (*r* = 0.28, *p* < 0.001) and the anxiety model (*r* = 0.17, *p* < 0.05). Shown in Figs. 3 and 4, “positive tone” was the most important linguistic feature for predicting depressive symptoms with negative directionality (i.e., higher values lead to lower symptoms). First-person plural pronouns, “time”, “motion”, “substance” and “wellness” words also emerged as somewhat important to the model. The most important linguistic features with positive directionality (i.e., lower values lead to higher symptoms) were conjunctions, “function” “differentiation” and “death” words. The most important linguistic features for predicting anxiety symptoms with negative directionality were past-focussed words, positive tone, “drive” words, third-person singular pronouns, and “article” words (see Figs. 5 and 6). The most important features with positive directionality were “anxiety” words, conjunctions, linguistic words, “all-or-nothing” words, and “discrepancy” words. See Supplementary Material for the variable stability plots.

**Table 8:** Best performing across-task machine learning models using linguistic features to predict mental health symptoms

				Performance metrics			Correlation between observed and predicted test values		
Mental health symptoms	Best performing model	Recursive feature elimination	Demographic and clinical features	*RMSE*	*r*^*2*^	*MAE*	*r*	*p*	Hyperparameters for final fitted model
Depression	Random Forest	No	No	4.87	0.08	3.89	0.28	<0.001	mtry = 70
Anxiety	Random Forest	No	No	4.50	0.03	3.70	0.17	0.015	mtry = 30

### The impact of emotional text tasks (Task C and Task D) on participants’ mood and acceptability ratings

There were almost no immediate changes in participants’ mood, as measured by the MDMQ, after completing the emotion diary tasks (Task C, *P* = 0.057 to 0.987) except for higher levels of relaxation after the second completion (MD: 0.26, *df* = 895.81, *P* = 0.030) and higher levels of agitation after the third completion (MD: 0.34, *df* = 896.42, *P* = 0.008). For the expressive task (Task D), participants reported significantly greater fatigue (*P* = 0.001 to *P* = 0.045), lower levels of contentment (*P* = 0.000–0.007), higher levels of agitation (all *P* < 0.001), lower levels of energy (*P* = 0.002 to 0.40), feeling more unwell (*P* = 0.000 to 0.023) and lower levels of relaxation (all *P* < 0.001) after all completions (see Supplementary Material for more detail). There were no main effects of sequence or interaction effects for sequence by time on any of these findings.

Shown in Table 9, the acceptability ratings of the tasks were mixed. Across the whole sample, the expressive tasks (Task D) were significantly more difficult to complete (M: 3.0, SD: 1.9) than all other tasks (all *P* < 0.001). Participants were also significantly less willing to complete Task D (M: 4.7, SD: 1.9) when compared to all other tasks (all *P* ranged from <0.001 to 0.012). However, participants’ levels of interest and willingness were influenced by their allocation sequence. Participants in sequence 1 reported significantly greater interest in completing tasks when compared to sequences 3 (*P* = 0.026) and 4 (*P* = 0.006). Participants in sequence 1 were also significantly more willing to repeat the tasks when compared to sequences 3 (*P* = 0.036) and 4 (*P* = 0.036).

**Table 9:** Omnibus test results from mixed models assessing acceptability across tasks and sequences

	Variable	*df*_*num*_	*df*_*den*_	*F*	*P*
Ease of completion	Task	7	1306.66	37.51	<0.001
	Sequence	3	202.64	0.53	0.661
	Task * Sequence	21	1306.58	0.80	0.723
Level of interest	Task	7	1308.55	10.59	<0.001
	Sequence	3	206.74	4.75	0.003
	Task * Sequence	21	1308.49	0.89	0.610
Willingness to repeat	Task	7	1303.95	12.16	<0.001
	Sequence	3	202.94	3.33	0.021
	Task * Sequence	21	1303.89	3.22	<0.001

## Discussion

This experimental study examined the utility of several types of text data for generating valid and reliable linguistic markers of depression and anxiety when collected among symptomatic individuals, using a trial design that accounted for sequence and potential task effects. The findings only partially supported the hypotheses. There were no linguistic features that were consistently associated with depressive or anxiety symptoms within tasks or across all tasks. At the task level, five linguistic features were associated with depressive symptoms but these features were elicited in only three of the eight tasks and associations were weak. Notably, the features found to be associated with depressive symptoms were different for each task and there was only some degree of reliability of these features within the repeated tasks. When all tasks were combined, six linguistic features were associated with depressive symptoms. However, only two of these features were correlated at the task-level. Only one linguistic feature was associated with anxiety, but this feature was not significant at the task level. In all the machine learning models, predicted values were weakly associated with actual values for both depressive and anxiety symptoms. Positive tone emerged as the most important linguistic feature for predicting variance in depressive symptoms, yet this feature was not significant within any of the tasks. Linguistic features were much less important in the anxiety model than the depression model. Overall, these findings indicate that the linguistic markers of depression and anxiety shift in response to contextual factors and the qualitative nature of the text data analysed.

Notably, the results do not replicate the findings of many past studies that used the same types of text tasks (e.g., see refs. ^17,19–21^). In this trial, only three of the eight tasks revealed significant linguistic markers of depressive and anxiety symptoms when controlling for the false discovery rate: social media posts, personal description of one’s self, and description of one’s friends. Higher depressive symptoms were associated with fewer references to others during the social media tasks, which may reflect a cognitive and emotional profile marked by heightened sadness and reduced social orientation. The increased use of “want” words in the friends description task may indicate a greater yearning for social connections, which aligns with the well-documented tendency for individuals with more severe depression to experience greater social isolation and loneliness. The increased use of cognition words in the self-description task indicates that describing oneself may have demanded greater cognitive effort in individuals with higher symptoms, potentially stemming from a diminished ability of more severely depressed adults to articulate a unified self-concept. This type of writing may therefore be useful for identifying a distinct cognitive processing pattern among depressed adults, which does not emerge in personal communications or emotion-processing tasks. Together, the findings suggest that some types of word use may indeed reflect the low mood (e.g., increased negative tone), social withdrawal (e.g. reduced plural pronouns), and impaired desire (e.g. greater use of wanting words) that characterise depression. However, the markers that were significantly correlated with depressive symptoms were unique to each of the tasks and not all were significant in the combined analysis. There was also limited reliability for the linguistic correlates in the social media data, indicating that the robustness of these features may be influenced by the amount of data available for repeated analyses. As such, the results of this trial do not provide definitive evidence of the strength of linguistic markers of depressive symptoms from within the included tasks.

Overall, there was poor generaliseability for the linguistic markers examined in this trial as none of the markers were consitent within and across all tasks. This provides further evidence of a task effect and suggests that this approach to digital phenotyping may not yet yield great benefit for detecting depressive symptoms unless the contextual factors or priming effects within the tasks are accounted for. Thus, researchers wishing to use either organic or simulated text data for the digital phenotyping of mental illness need to carefully consider the conditions under which the text data was generated and account for variations in the quantity and frequency of data collected. While the results do not point to a single or set of valid and reliable linguistic markers of depressive or anxiety symptoms, the overall degree of positive tone expressed in individuals’ text data may have some utility for predicting depressive scores at the group-level. However, this finding also requires further validation in larger samples with varying types of text data, collected again at varying frequencies and over longer periods of time, given it didn’t emerge as a within-task marker. Furthermore, anxiety-focussed writing tasks (e.g., descriptions of anxious moments)^16^ or psychotherapy content (e.g. therapy transcripts)^50–52^ may be more useful for identifying the linguistic correlates of anxiety severity. There may also be greater potential for writing tasks to help differentiate subtypes of anxiety, such as social anxiety^53^, health anxiety^54^, or trait-like anxiety^55^, as the current study focussed on generalised anxiety symptoms only.

As a goal of digital phenotyping is personalised medicine, future research may benefit from focusing greater efforts on examining person-level variations in linguistic expression over time. This may be particualarly relevant to linguistic analysis, given prior work has showed group-level models do not have strong predictive value for individuals^56^. However, deciding which features to include in analyses of this kind remains a serious methodological challenge^57^. Interestingly, demographics and clinical characteristics were absent from some of the best-fitting models, indicating that this information did not always improve the accuracy of the machine learning. This may have broader implications for machine learning approaches in digital phenotyping, whereby more exploratory work is needed to understand any subtle interactions between personal or illness characteristics, digital signals, and different use cases. For example, when using digital phenotyping for monitoring an individual when observers may already have some knowledge of their mental state or circumstance (e.g. monitoring in clinical practice) in contrast to using digital phenotyping for tracking the mental health of a population with unknown parameters. Some of the linguistic features that were important in the modelling were somewhat uninterpretable (e.g., motion words), limiting the informativeness of our approach for contributing to the theoretical knowledge of depressive states in adults. However, the emergence of other features, such as conjunctions and function words, suggest that future work should include syntax analysis to enhance our understanding of the relationships between mental illness and the structural dimensions of linguistic expression^58^.

Lastly, while rates of task completion were high in this study, acceptability of the tasks were mixed. In particular, the expressive tasks that focused on memory recall were found to negatively impact various dimensions of participants’ mood, after all occasions, and were poorly accepted by participants. Although this task did not explicitly ask participants to focus on a negative emotional event, given the negative cognitive biases caused by depression, it is highly likely that many participants chose to reflect on negative events. Qualitative analysis of participants’ responses to this task may help to provide greater insight of the topics discussed that may be contributing to the negative effects on mood. Future research should also carefully examine the ethical implications and design implications of using some structured expressive tasks for the collection of linguistic markers among symptomatic samples.

A key strength of the current study was the use of a cross-over trial design with repeated measures, as the results confirmed that the order in which text tasks were administered impacted participants’ completions as well as their levels of interest and engagement. Within-task and across task analyses also enabled greater exploration of the validity and reliability of the linguistic features by providing a larger testing set of varying text data. However, the generalisability of the findings may be limited, given most participants were English-literate females. Models applied to samples with varying degrees of English proficiency may have different results. As our design focused on individuals with elevated symptoms, different results may also be found when attempting to use linguistic markers to distinguish depressed individuals from non-depressed, or stratification of sub-types of depression, or change over time^59^. There is also evidence of potential floor effects for some of the linguistic features when examined by task that were not present when all tasks were combined. This suggests that it may have been the tasks eliciting lower base rates of some linguistic features rather than individuals’ depressive and anxiety symptoms. This may also be related to the word counts, given that on average, participants produced fewer than 300 words for most of the tasks. While the cleaning of data helped to ensure the accuracy of the LIWC, the text data were not passively collected. As such, the measurement effects within this study may not be found when other types of text data of varying lengths and collection methods are examined or updated versions of LIWC are applied to the data in the future. There may also be important differences between tasks completed on desktop versus mobile devices or with assistive technologies (e.g. dictation, predictive text), which warrants further investigation in future research. Lastly, the LIWC tool is a crude instrument. Emerging research has found that some large language models have outperformed LIWC features when identifying mental health outcomes^59,60^, although LIWC features have remained valuable and complementary to some models^51^. Future work should continue to explore the informativeness of LIWC when compared to advanced contemporary methods of linguistic analysis for mental health.

The findings provide evidence of a task-effect on linguistic expression whereby the markers generated in one context may not be generaliseable to another. This underscores the need for researchers to account for contextual variations when attempting to use linguistic analysis for digital phenotyping of mental illness. While positive tone shows some potential for predicting depressive symptoms at the group level, its utility requires validation in larger samples with diverse text data collected passively. With the rapid growth of large language models for mental health^61,62^ and linguistic analysis of psychotherapy interventions for outcomes tracking^50,63–65^, future research should focus on individual-level variations in linguistic expression over time and across modalities, addressing methodological challenges in feature selection. Additionally, careful examination of task acceptability, particularly expressive writing tasks, is warranted for collecting linguistic markers among symptomatic samples.

## Supplementary information

Supplementary material

## Data Availability

Data can be requested by contacting Professor Bridianne O'Dea bridianne.odea@flinders.edu.au who will enable access in accordance with the UNSW Human Research Ethics Approvals.
