# Supplementary material for: A randomised cross over trial examining the linguistic markers of depression and anxiety in symptomatic adults
Source: Npj Ment Health Res. 2025 Jul 19;4:30. doi: 10.1038/s44184-025-00140-y (PMC12276349; doi:10.1038/s44184-025-00140-y)
Supplement: Supplementary file 1 — Supplementary material [file 44184_2025_140_MOESM1_ESM.pdf]

SUPPLEMENTARY MATERIAL

Results

Table S1. Participant characteristics within sequences.

	Total sample		Sequence 1		Sequence 2		Sequence 3		Sequence 4	
	**M**	**SD**	**M**	**SD**	**M**	**SD**	**M**	**SD**	**M**	**SD**
Age	39.33	12.37	40.64	11.93	37.63	11.59	37.90	12.27	41.00	13.51
PHQ9	13.50	4.13	13.34	4.20	14.11	4.19	13.00	3.91	13.54	4.22
GAD7	10.83	4.51	11.16	4.54	11.33	4.12	10.19	4.97	10.60	4.42
	**N**	**%**	**N**	**%**	**N**	**%**	**N**	**%**	**N**	**%**
Female	163	74.8	43	76.8	40	74.1	36	69.2	44	78.6
University education	105	48.2	36	64.3	21	38.9	22	42.3	26	46.4
Married/Partnered	115	52.8	32	57.1	27	50.0	29	55.8	27	48.2
Paid employment	163	74.8	41	73.2	45	83.3	39	75.0	38	67.9
Born in Australia	175	80.3	42	75.0	41	75.9	46	88.5	46	82.1
First Nations	5	2.3	1	1.8	0	0.0	1	1.9	3	5.4
LGBTQI+	45	20.6	12	21.4	13	24.1	10	19.2	10	17.9
English only	198	90.8	47	83.9	50	92.6	51	98.1	50	89.3
Literacy support (Never)	186	85.3	48	85.7	45	83.3	43	82.7	50	89.3
Formally diagnosed mental illness	202	92.7	54	96.4	50	92.6	46	88.5	52	92.9
Age of onset	19.91	10.83	22.80	11.02	19.10	11.11	20.47	11.23	17.19	9.64
Age of diagnosis	26.94	10.93	25.97	10.36	24.24	10.85	27.61	11.76	27.02	10.64
Taking prescribed medication	130	59.6	38	67.9	33	61.1	27	51.9	31	55.4
Likely case of MDD	193	88.5	48	85.7	48	88.9	47	90.4	50	89.3
Likely case of GAD	134	61.5	37	66.1	39	72.2	26	50.0	32	57.1

Table S2. Mean values (proportions) for LIWC-22 features averaged across tasks

Mean (SD)	Category	Abbreviation	Descriptor
122 (69.9)	Word count	WC	Total word count
15.7 (5.3)	Words per sentence	WPS	Average words per sentence
14.8 (2.6)	Big words	BigWords	Percent words 7 letters or longer
94 (1.7)	Dictionary words	Dic	Percent words captured by LIWC
75.6 (3.1)	Linguistic Dimensions	Linguistic	
59.5 (3.8)	Total function words	function	the, to, and, I
16.9 (2.8)	Total pronouns	pronoun	I, you, that, it
12.4 (2.3)	Personal pronouns	ppron	I, you, my, me
8.1 (2)	1st person singular	i	I, me, my, myself
0.8 (0.6)	1st person plural	we	we, our, us, lets
1.3 (0.8)	2nd person	you	you, your, u, yourself
1.4 (1)	3rd person singular	shehe	he, she, her, his
0.6 (0.5)	3rd person plural	they	they, their, them, themsel*
4.6 (1.5)	Impersonal pronouns	ipron	that, it, this, what
12.8 (1.9)	Determiners	det	the, at, that, my
5.7 (1.3)	Articles	article	a, an, the, alot
1 (0.9)	Numbers	number	one, two, first, once
12.9 (1.7)	Prepositions	prep	to, of, in, for
10.9 (1.7)	Auxiliary verbs	auxverb	is, was, be, have
6.7 (1.5)	Adverbs	adverb	so, just, about, there
7.7 (1.4)	Conjunctions	conj	and, but, so, as
1.9 (0.8)	Negations	negate	not, no, never, nothing
20.1 (2.4)	Common verbs	verb	is, was, be, have
6.9 (2.7)	Common adjectives	adj	more, very, other, new
4.3 (1.4)	Quantities	quantity	all, one, more, some
4.9 (1.4)	Drives	Drives	we, our, work, us
2.9 (1.2)	Affiliation	affiliation	we, our, us, help
1.3 (0.6)	Achievement	achieve	work, better, best, working
0.7 (0.4)	Power	power	own, order, allow, power
13.8 (2.4)	Cognition	Cognition	is, was, but, are
1.3 (0.7)	All-or-none	allnone	all, no, never, always
12.4 (2.3)	Cognitive processes	cogproc	but, not, if, or, know
3 (1)	Insight	insight	know, how, think, feel
1.4 (0.7)	Causation	cause	how, because, make, why
2.1 (0.7)	Discrepancy	discrep	would, can, want, could
2.6 (1.1)	Tentative	tentat	if, or, any, something
0.8 (0.6)	Certitude	certitude	really, actually, of course, real
3.6 (1.1)	Differentiation	differ	but, not, if, or
0.1 (0.1)	Memory	memory	remember, forget, remind, forgot
7.7 (3.3)	Affect	Affect	good, well, new, love
4.5 (1.4)	Positive tone	tone_pos	good, well, new, love

2.9 (3.1)	Negative tone	tone_neg	bad, wrong, too much, hate
3.7 (3)	Emotion	emotion	good, love, happy, hope
1.7 (0.8)	Positive emotion	emo_pos	good, love, happy, hope
1.8 (2.9)	Negative emotion	emo_neg	bad, hate, hurt, tired
0.5 (1)	Anxiety	emo_anx	worry, fear, afraid, nervous
0.2 (0.2)	Anger	emo_anger	hate, mad, angry, frustr*
0.3 (0.5)	Sadness	emo_sad	:(, sad, disappoint*, cry
0.2 (0.3)	Swear words	swear	shit, fuckin*, fuck, damn
11 (2.6)	Social processes	Social	you, we, he, she
3.5 (1.4)	Social behavior	socbehav	said, love, say, care
0.9 (1)	Prosocial behavior	prosocial	care, help, thank, please
0.4 (0.9)	Politeness	polite	thank, please, thanks, good morning
0.2 (0.2)	Interpersonal conflict	conflict	fight, kill, killed, attack
0.2 (0.3)	Moralization	moral	wrong, honor*, deserv*, judge
1.2 (0.6)	Communication	comm	said, say, tell, thank*
7.2 (1.9)	Social referents	socrefs	you, we, he, she
0.9 (0.7)	Family	family	parent*, mother*, father*, baby
0.5 (0.4)	Friends	friend	friend*, boyfriend*, girlfriend*, dude
1.5 (1)	Female references	female	she, her, girl, woman
0.8 (0.8)	Male references	male	he, his, him, man
0.2 (0.2)	Culture	Culture	car, united states, govern*, phone
0 (0.1)	Politics	politic	united states, govern*, congress*, senat*
0 (0)	Ethnicity	ethnicity	american, french, chinese, Indian
0.1 (0.2)	Technology	tech	car, phone, comput*, email*
3 (1.1)	Lifestyle	lifestyle	work, home, school, working
0.9 (0.6)	Leisure	leisure	game*, fun, play, party*
0.5 (0.4)	Home	home	home, house, room, bed
1.3 (0.8)	Work	work	work, school, working, class
0.4 (0.3)	Money	money	business*, pay*, price*, market*
0.1 (0.3)	Religion	relig	god, hell, christmas*, church
3.1 (1.9)	Physical	physical	medic*, food*, patients, eye*
1.4 (1.6)	Health	health	medic*, patients, physician*, health
0.3 (0.3)	Illness	illness	hospital*, cancer*, sick, pain
0.1 (0.1)	Wellness	wellness	healthy, gym*, supported, diet
0.4 (0.7)	Mental health	mental	mental health, depressed, suicid*, trauma*
0.1 (0.2)	Substances	substances	beer*, wine, drunk, cigar*
0 (0.2)	Sexual	sexual	sex, gay, pregnan*, dick
0.8 (0.6)	Food	food	food*, drink*, eat, dinner*
0.1 (0.2)	Death	death	death*, dead, die, kill

0.5 (0.3)	Need	need	have to, need, had to, must
0.5 (0.3)	Want	want	want, hope, wanted, wish
0.9 (0.4)	Acquire	acquire	get, got, take, getting
0.3 (0.5)	Lack	lack	don’t have, didn’t have, *less, hungry
0.3 (0.3)	Fulfilled	fulfill	enough, full, complete, extra
0.4 (1.6)	Fatigue	fatigue	tired, bored, don’t care, boring
0.1 (0.1)	Reward	reward	opportun*, win, gain*, benefit*
0.3 (0.9)	Risk	risk	secur*, protect*, pain, risk*
0.3 (0.3)	Curiosity	curiosity	scien*, look* for, research*, wonder
9.7 (1.8)	Allure	allure	have, like, out, know
10.2 (1.7)	Perception	Perception	in, out, up, there
0.6 (0.4)	Attention	attention	look, look* for, watch, check
1.9 (0.7)	Motion	motion	go, come, went, came
5.9 (1.4)	Space	space	in, out, up, there
1 (0.5)	Visual	visual	see, look, eye*, saw
0.3 (0.3)	Auditory	auditory	sound*, heard, hear, music
1.2 (0.7)	Feeling	feeling	feel, hard, cool, felt
4.9 (1.3)	Time	time	when, now, then, day
3.8 (1.2)	Past focus	focuspast	was, had, were, been
7 (1.6)	Present focus	focuspresent	is, are, I’m, can
1.8 (0.8)	Future focus	focusfuture	will, going to, have to, may
0.6 (0.6)	Conversational	Conversation	yeah, oh, yes, okay
0.4 (0.5)	Netspeak	netspeak	:), u, lol, haha*
0.2 (0.3)	Assent	assent	yeah, yes, okay, ok
0 (0.1)	Nonfluencies	nonflu	oh, um, uh, i i
0 (0.1)	Fillers	filler	rr*, wow, sooo*, youknow
16.3 (13.4)	All Punctuation	AllPunc	
6.8 (4)	Periods	Period	.
2.6 (1.7)	Commas (,)	Comma	,
1.4 (5.6)	Question Marks (?)	QMark	?
1.3 (7.3)	Exclamation Marks (!)	Exclam	!
3.1 (1.5)	Apostrophes (')	Apostro	‘
1.1 (1.1)	Other Punctuation	OtherP	:);-;@

Figure S1. Scatterplot of the correlation between first-person plural pronouns and depressive symptoms within Task B (social media posts).

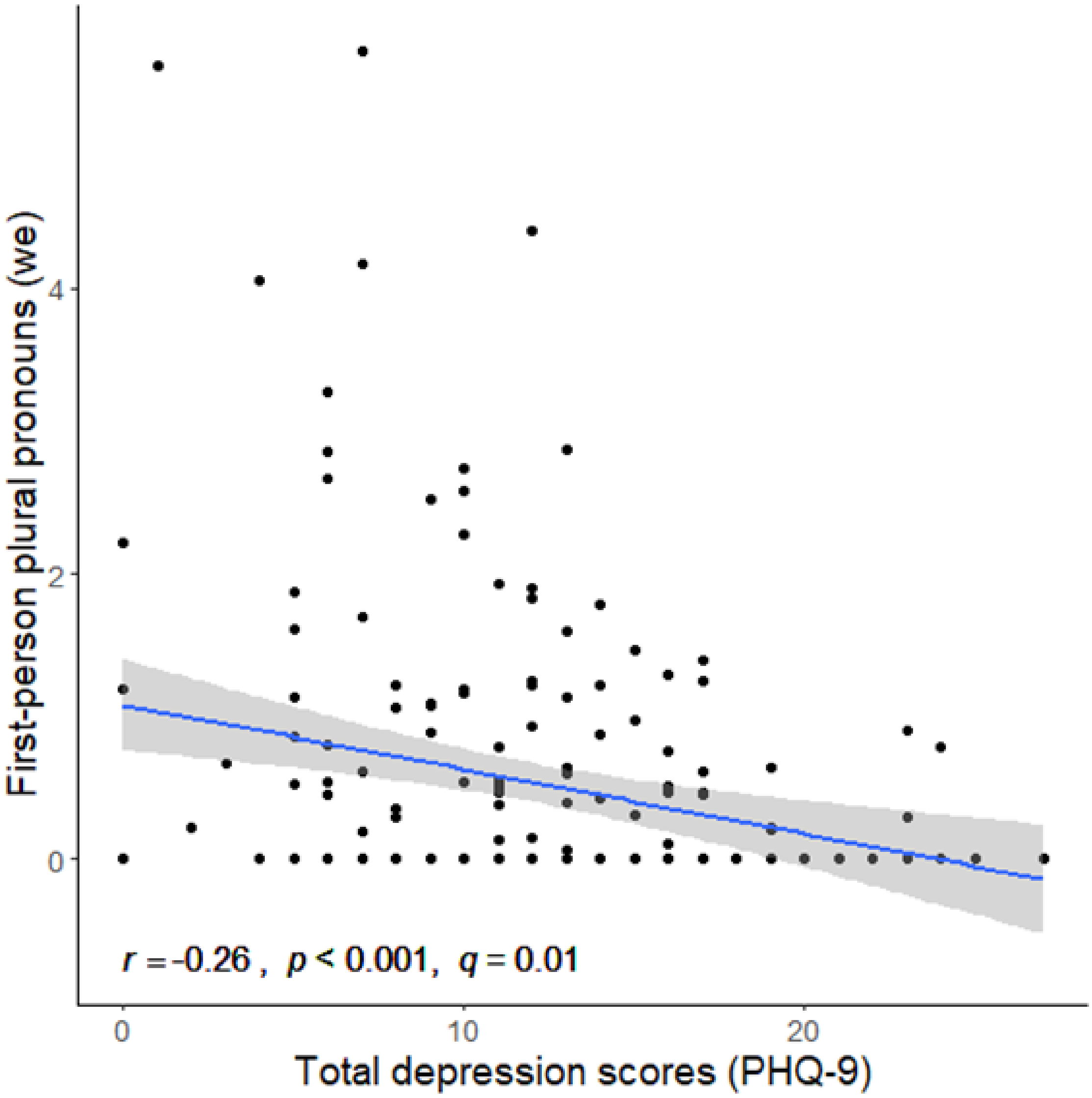

Figure S2. Scatterplot of the correlation between power words and depressive symptoms within Task B (social media posts) with outliers retained.

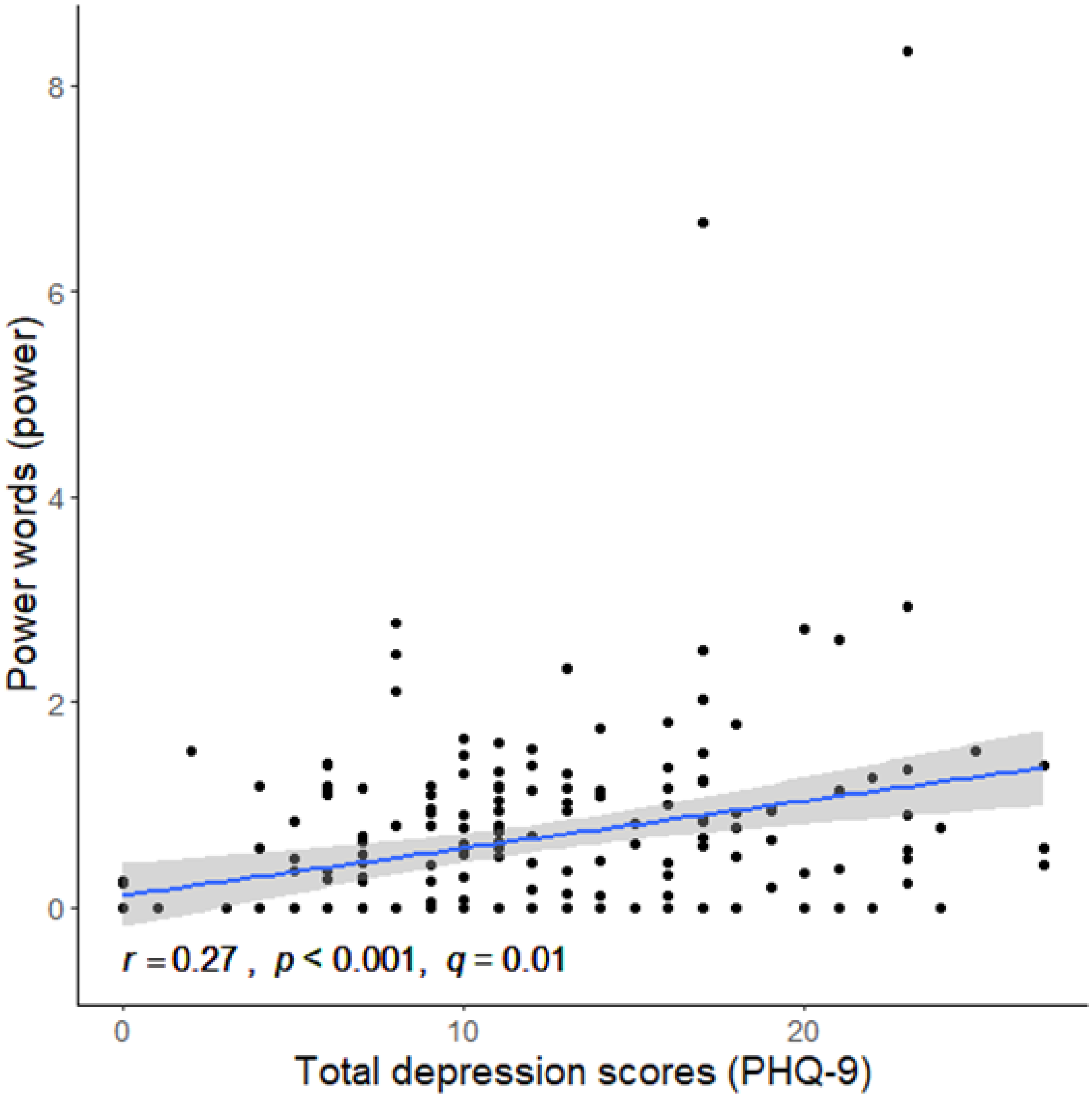

Figure S3. Scatterplot of the correlation between power words and depressive symptoms within Task B (social media posts) with outliers removed.

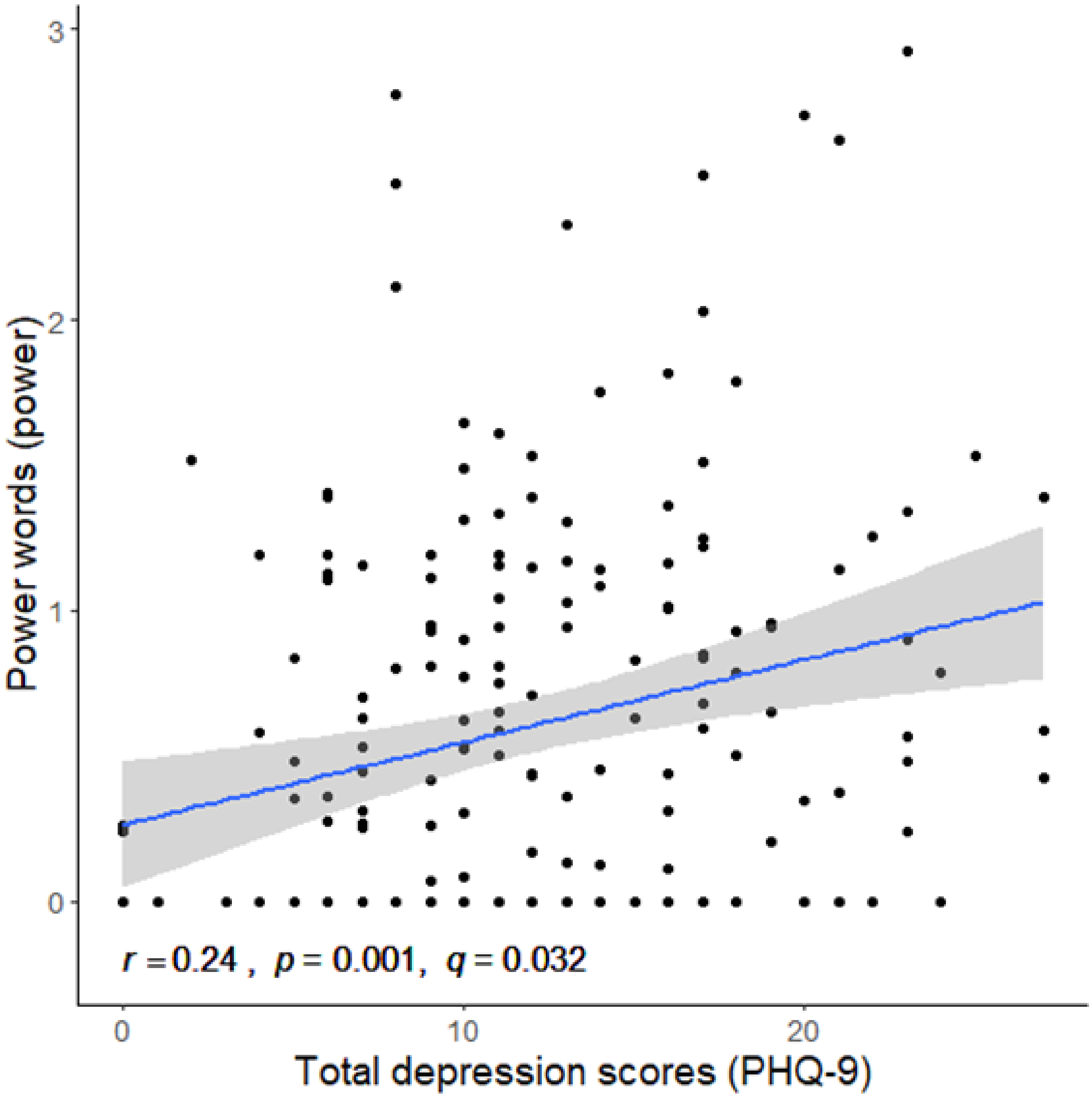

Figure S4. Scatterplot of the significant correlation between sadness words and depressive symptoms within Task B (social media posts) with outliers retained.

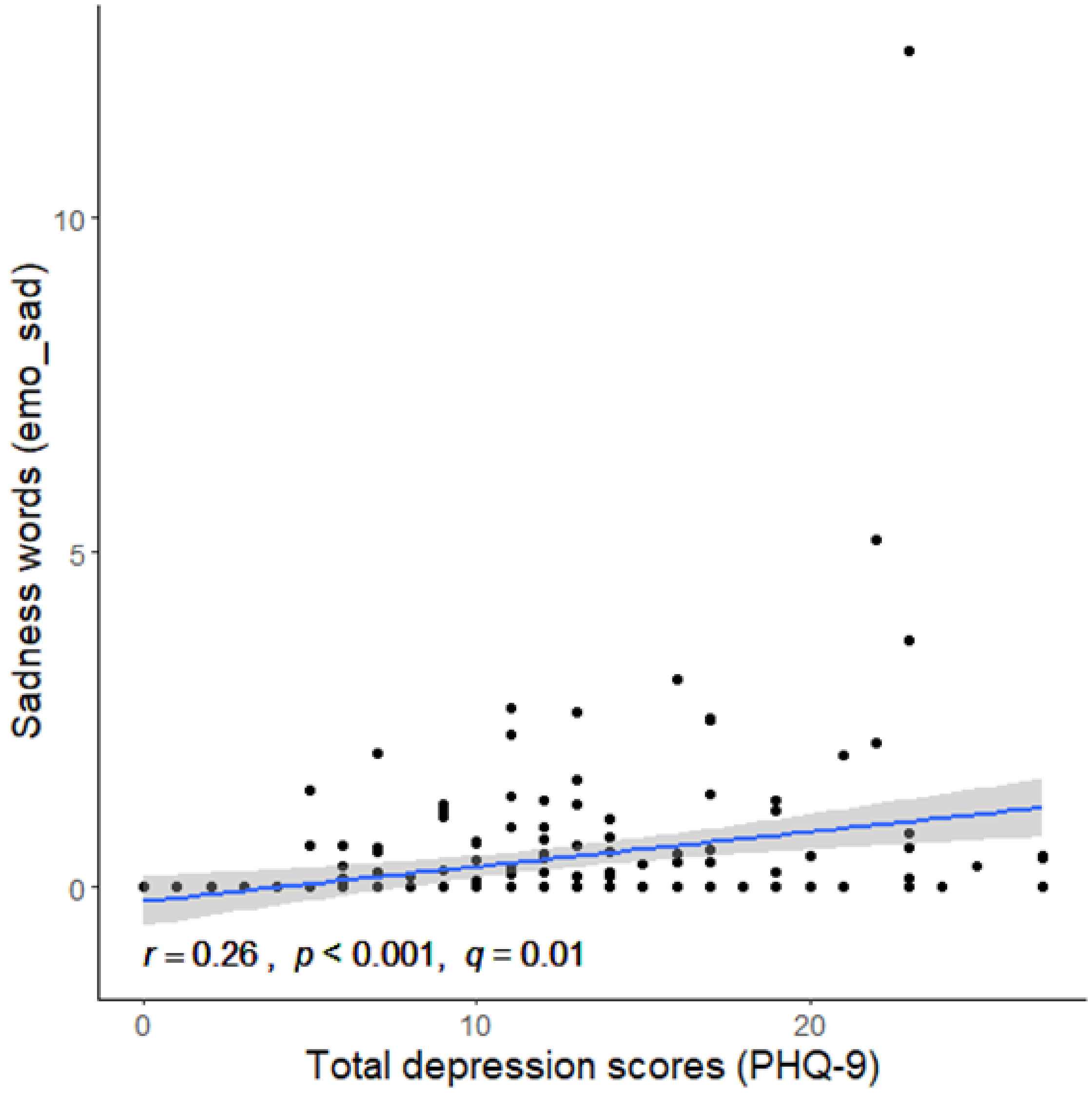

Figure S5. Scatterplot of the significant correlation between sadness words and depressive symptoms within Task B (social media posts) with outliers removed.

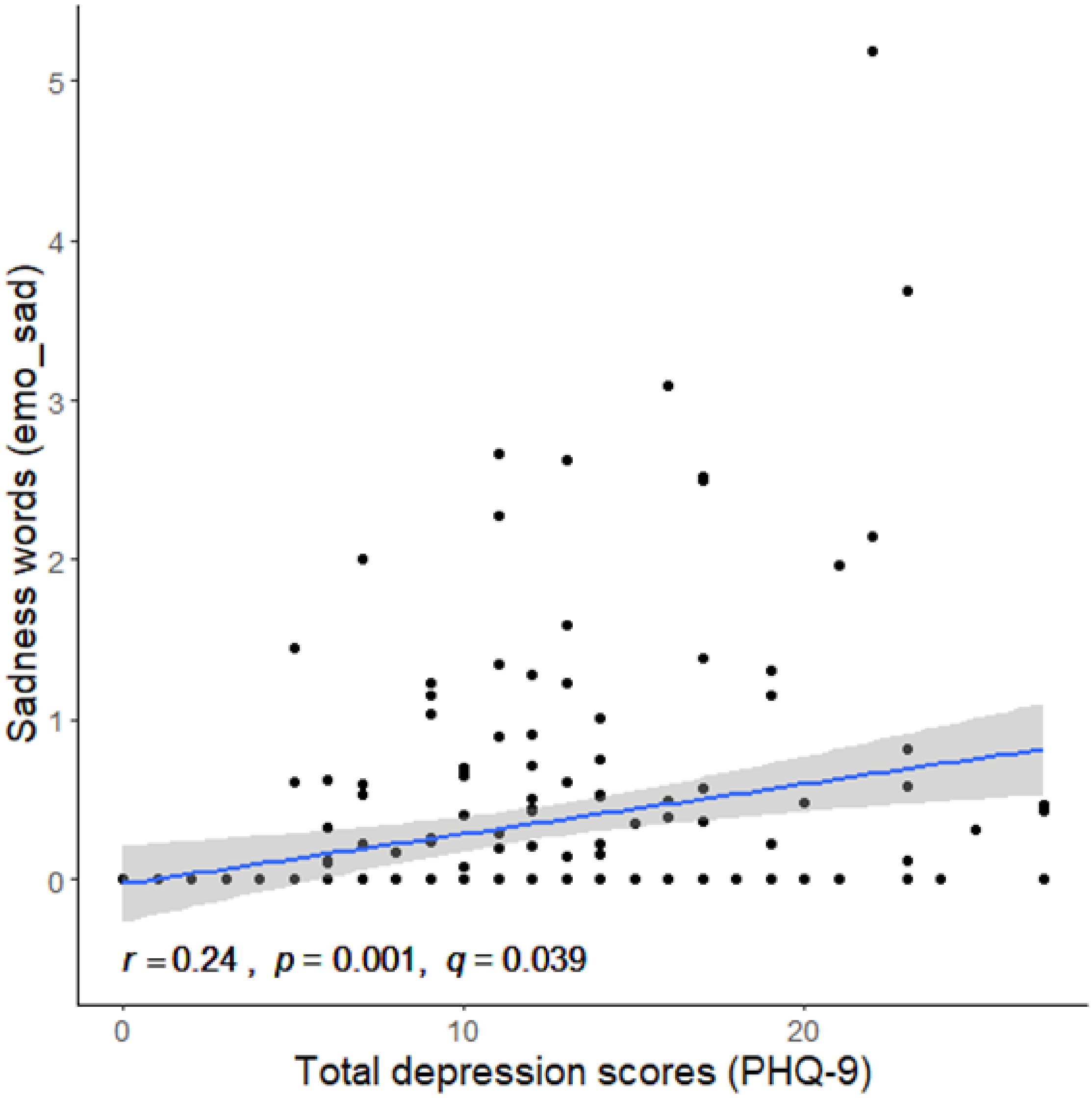

Figure S6. Scatterplot of the correlation between cognition words and depressive symptoms within Task V (description of one's self).

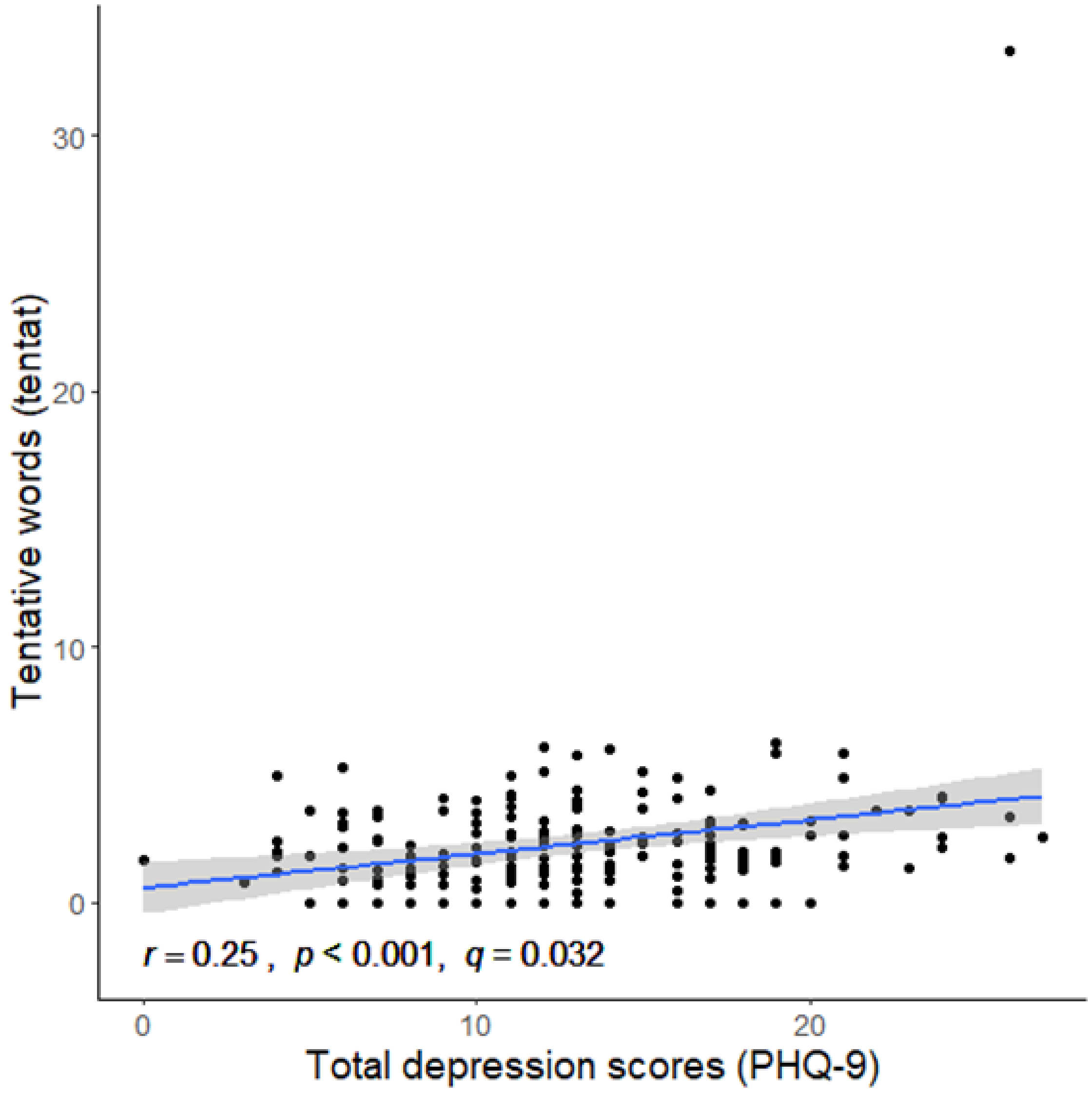

Figure S7. Scatterplot of the correlation between tentative words and depressive symptoms within Task V (description of one's self) with outliers retained.

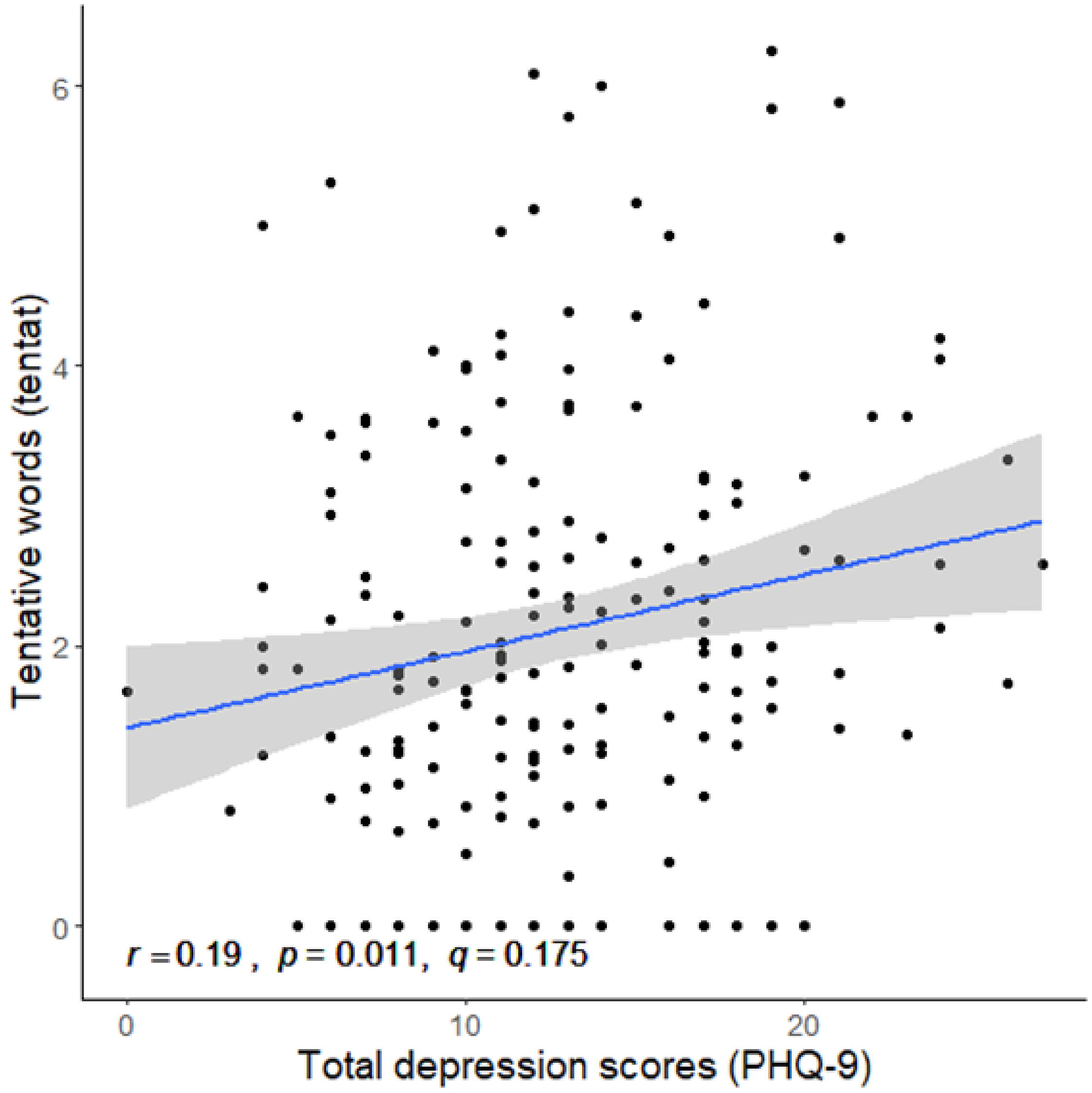

Figure S8. Scatterplot of the correlation between tentative words and depressive symptoms within Task V (description of one's self) with outliers removed.

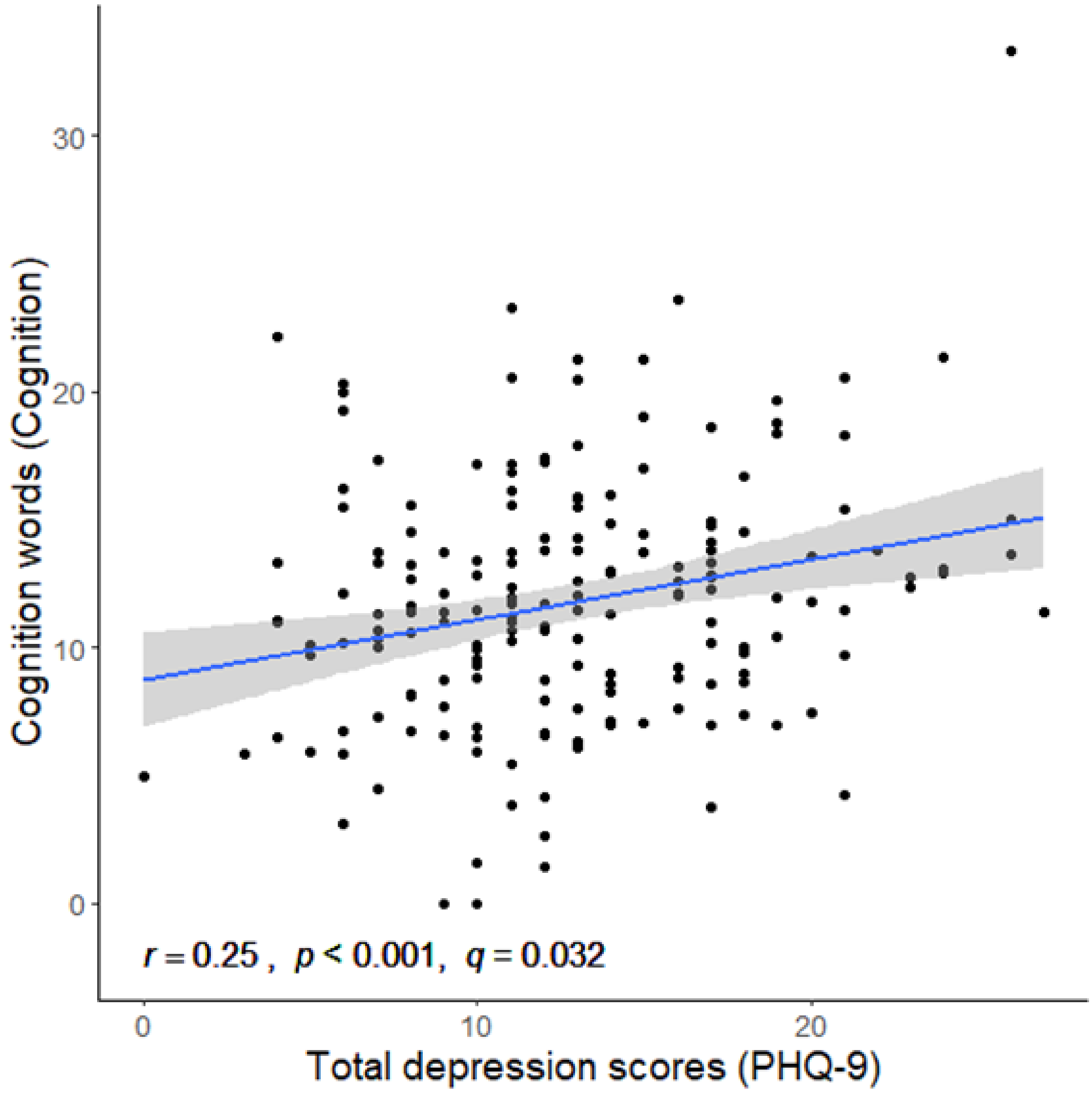

Figure S9. Scatterplot of the correlation between negative words and depressive symptoms within Task V (description of one's self) with outliers retained.

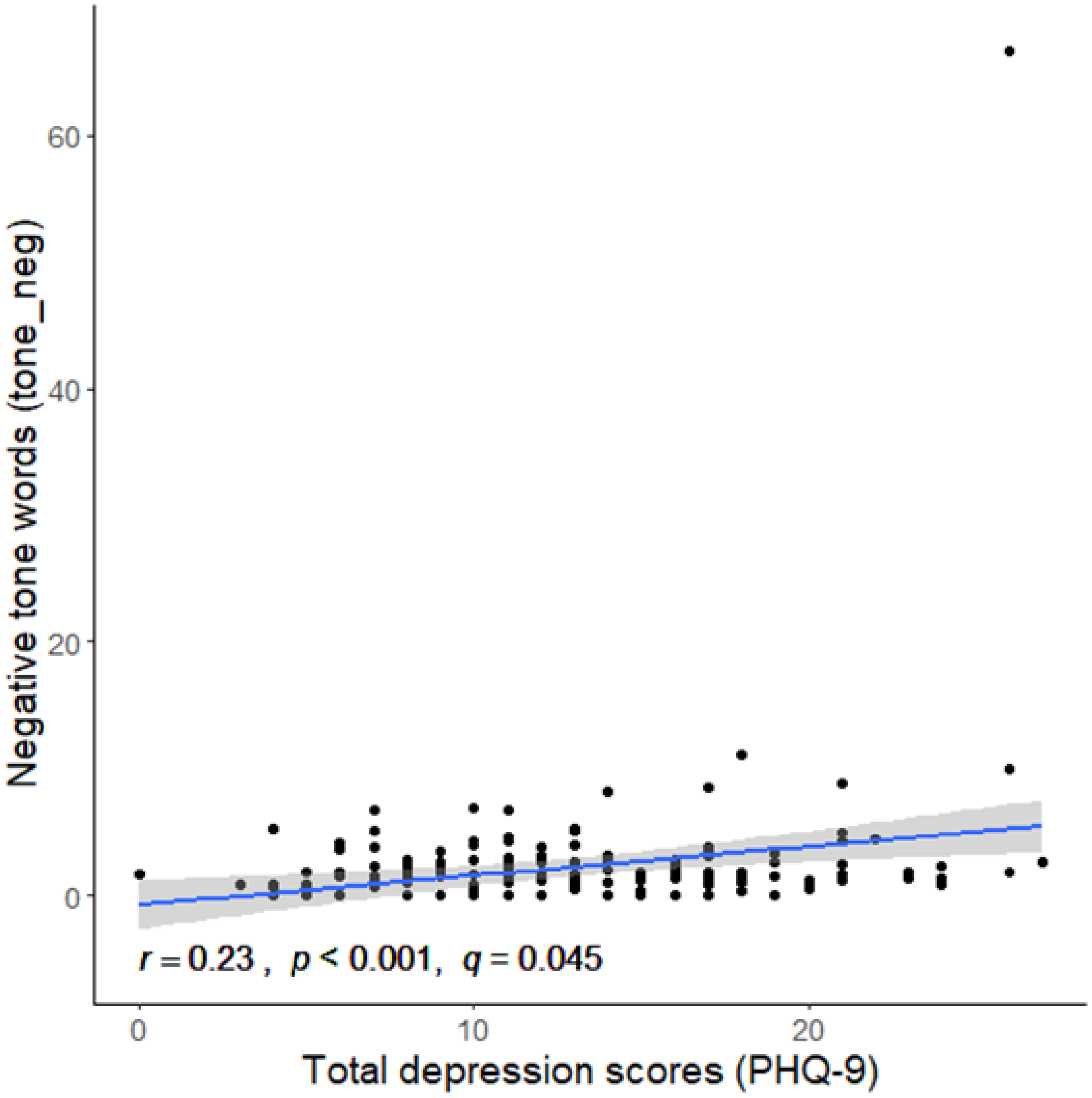

Figure S10. Scatterplot of the correlation between negative words and depressive symptoms within Task V (description of one's self) with outliers removed.

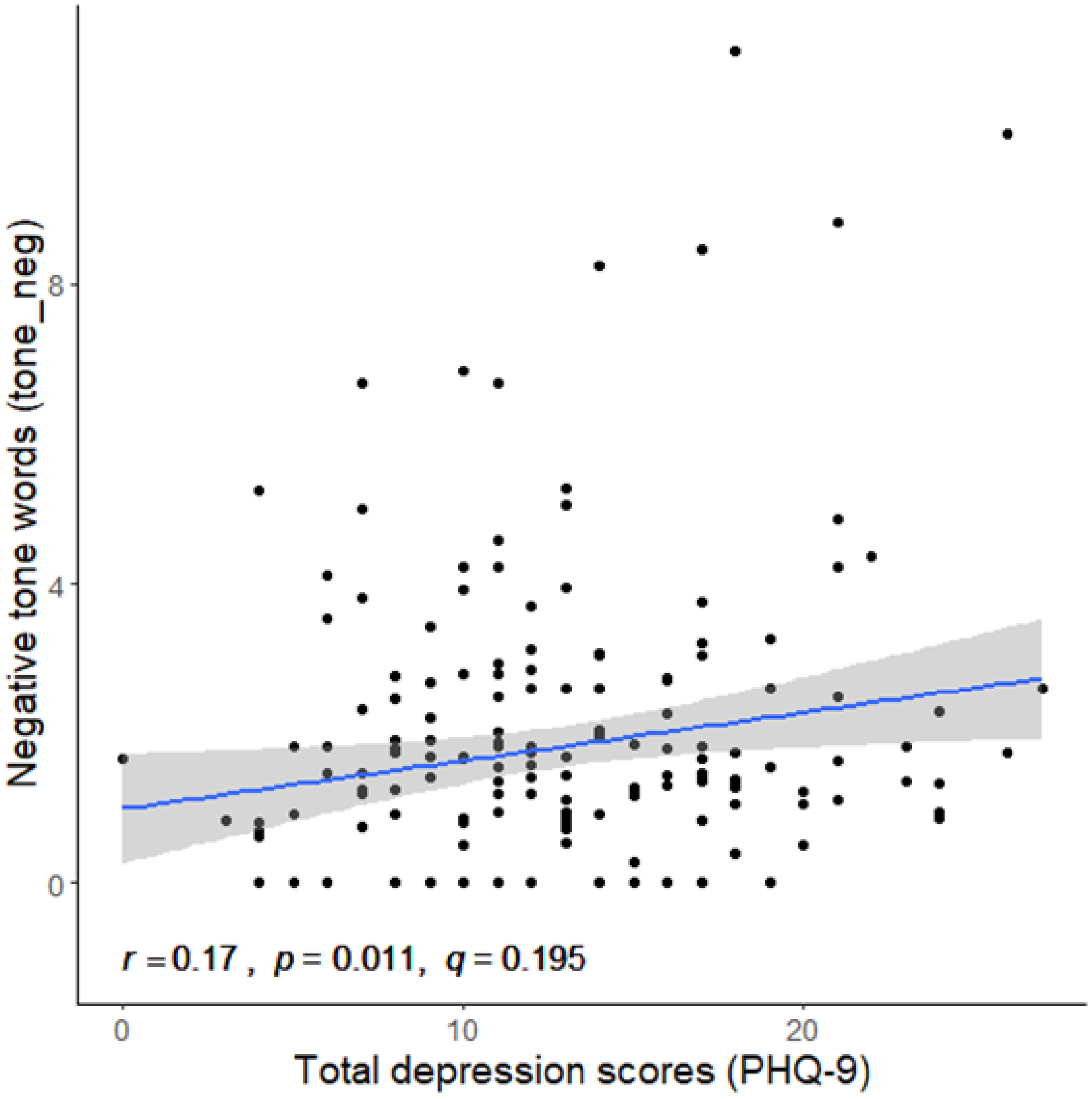

Figure S11. Scatterplot of the correlation between want words and depressive symptoms within Task W (description on one's friends).

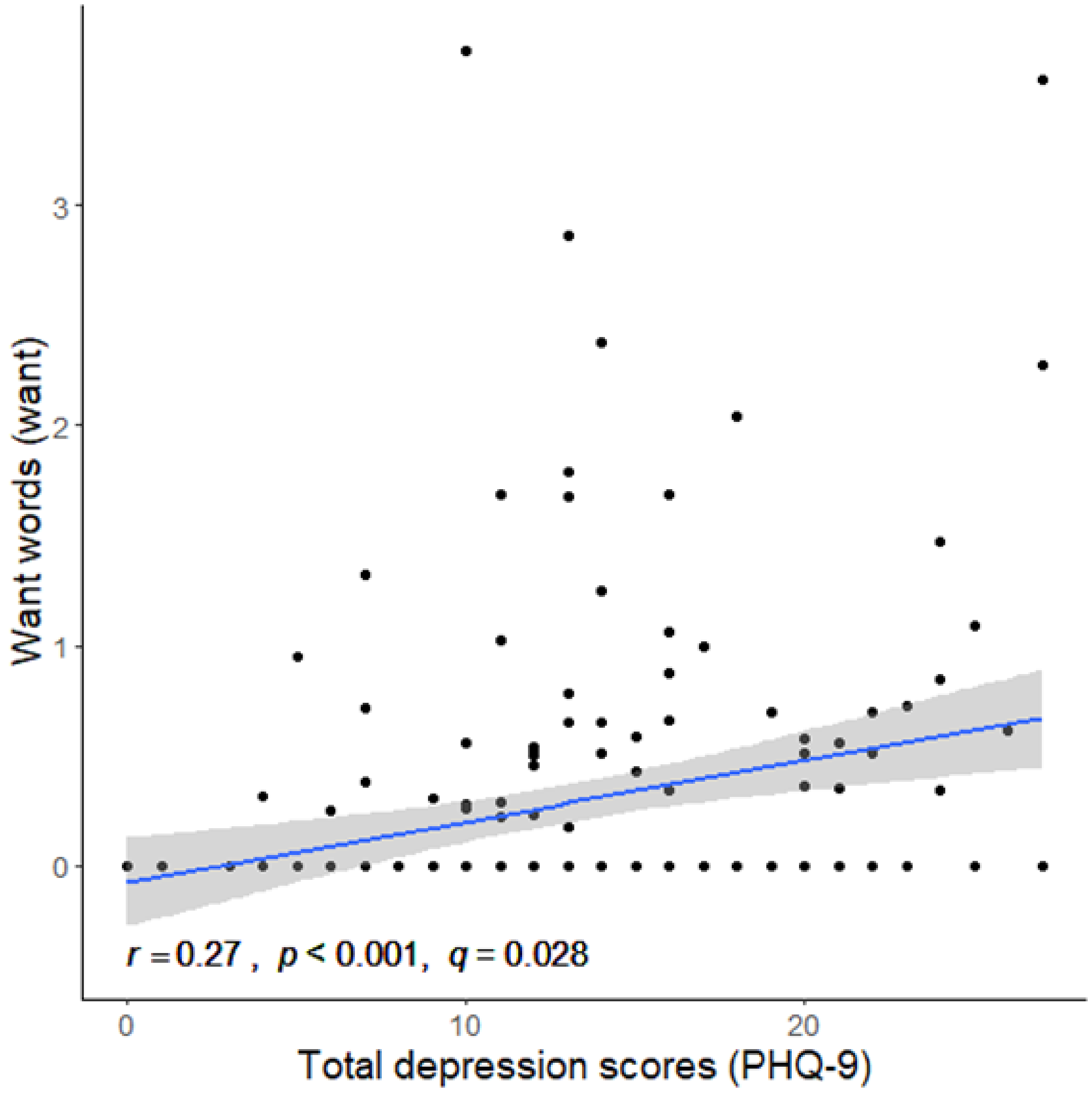

Figure S12. Feature importance using mean SHAP values from best performing machine learning models within tasks for predicting depressive symptoms.

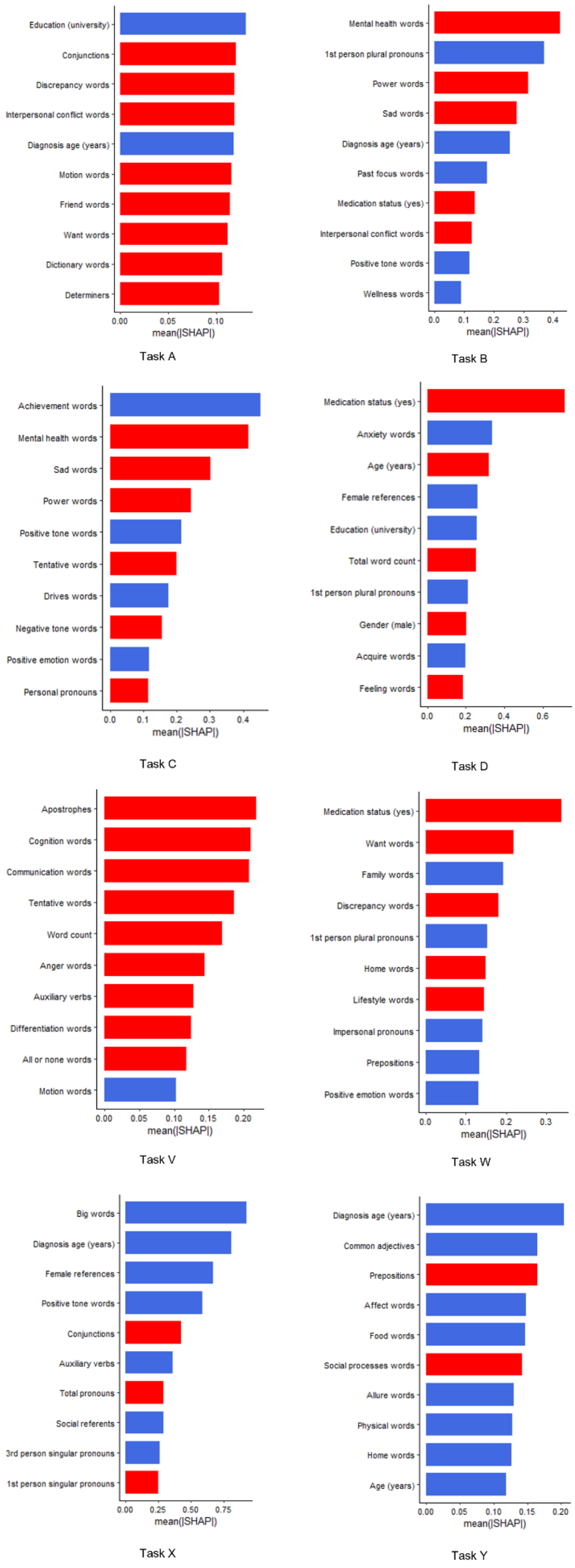

Figure Legend: Higher SHAP values indicate greater importance to modelling. Blue indicates features that have negative associations with symptoms, Red indicates features that have positive associations with symptoms.

Figures S13. Feature importance using mean SHAP values from best performing machine learning models within tasks for predicting anxiety symptoms.

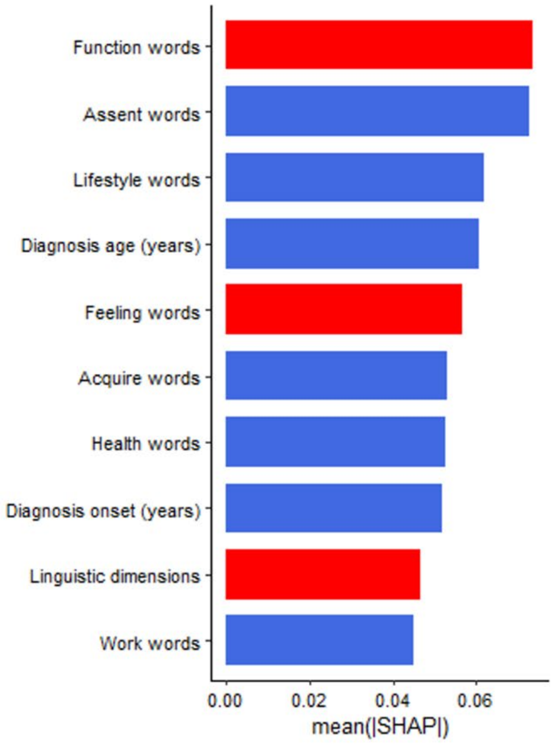

Task A

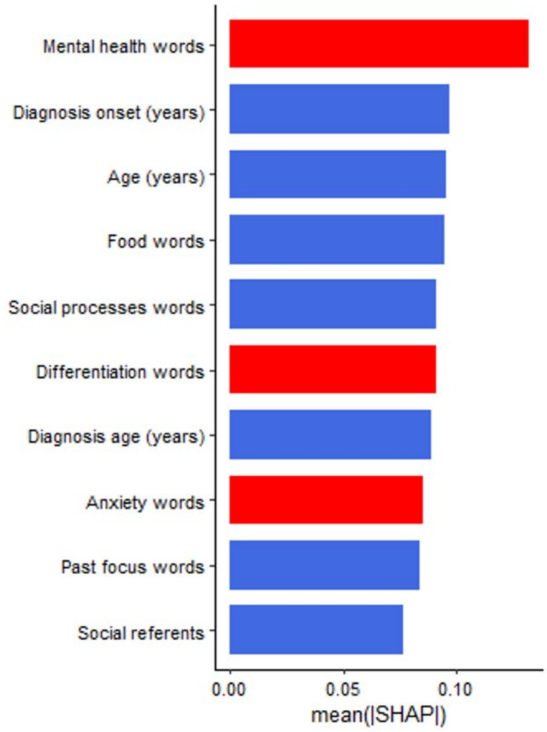

Task B

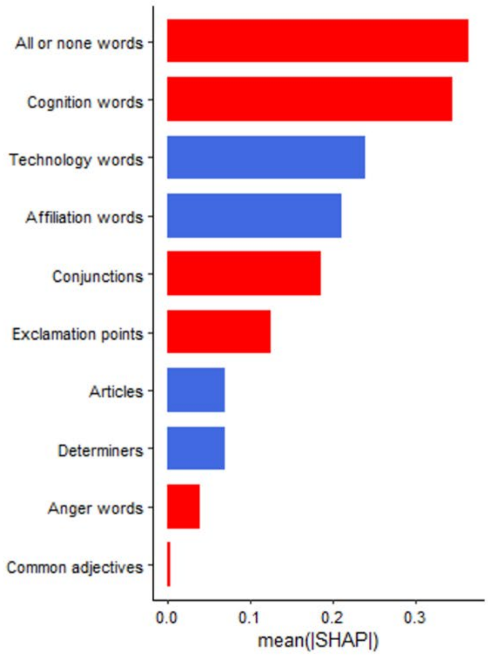

Task V

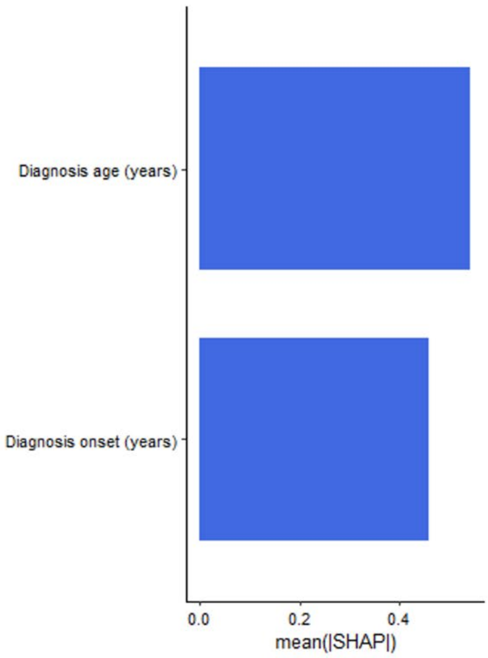

Task X

Figure Legend: Higher SHAP values indicate greater importance to modelling. Blue indicates features that have negative associations with symptoms, Red indicates features that have positive associations with symptoms.

Figure S14. Individual SHAP values for best performing machine learning models within tasks predicting depressive symptoms.

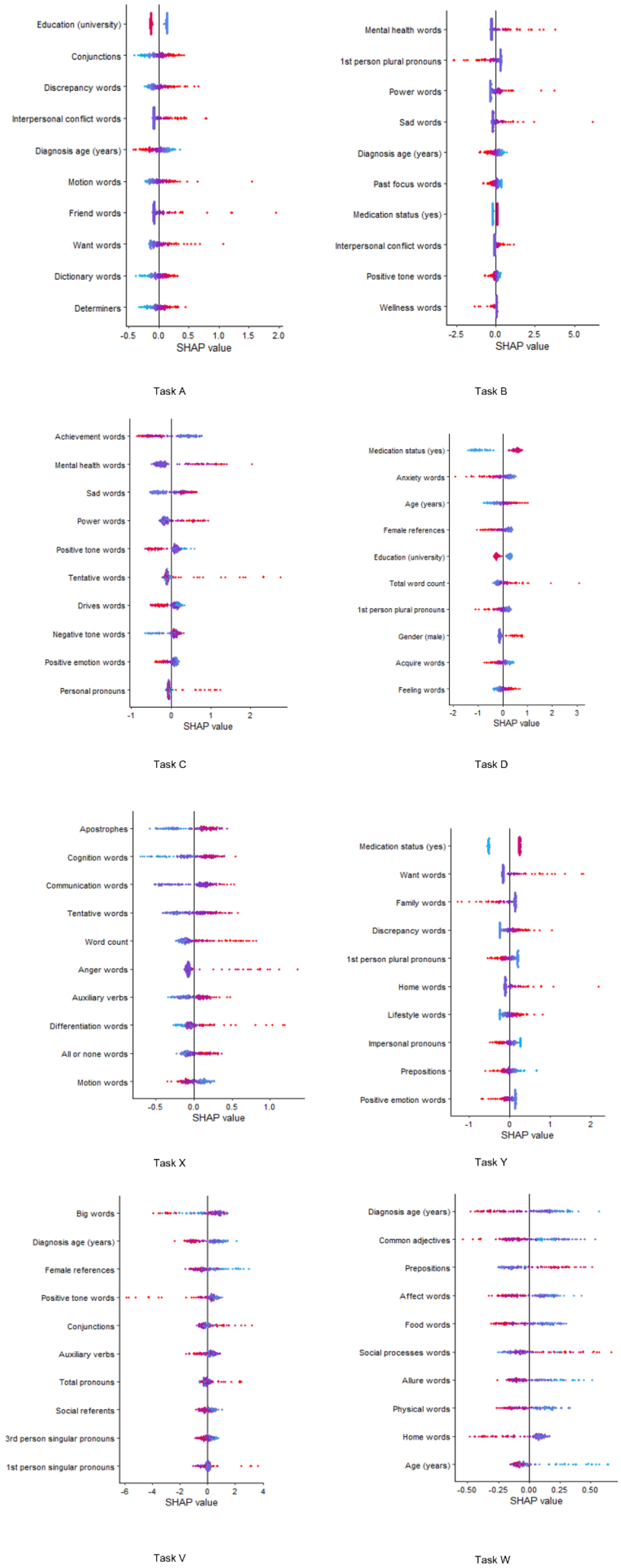

Figure Legend: Higher SHAP values indicate greater importance to modelling. Blue indicates low importance, Red indicates high importance.

Figure S15. Individual SHAP values for best performing machine learning models within tasks predicting anxiety symptoms.

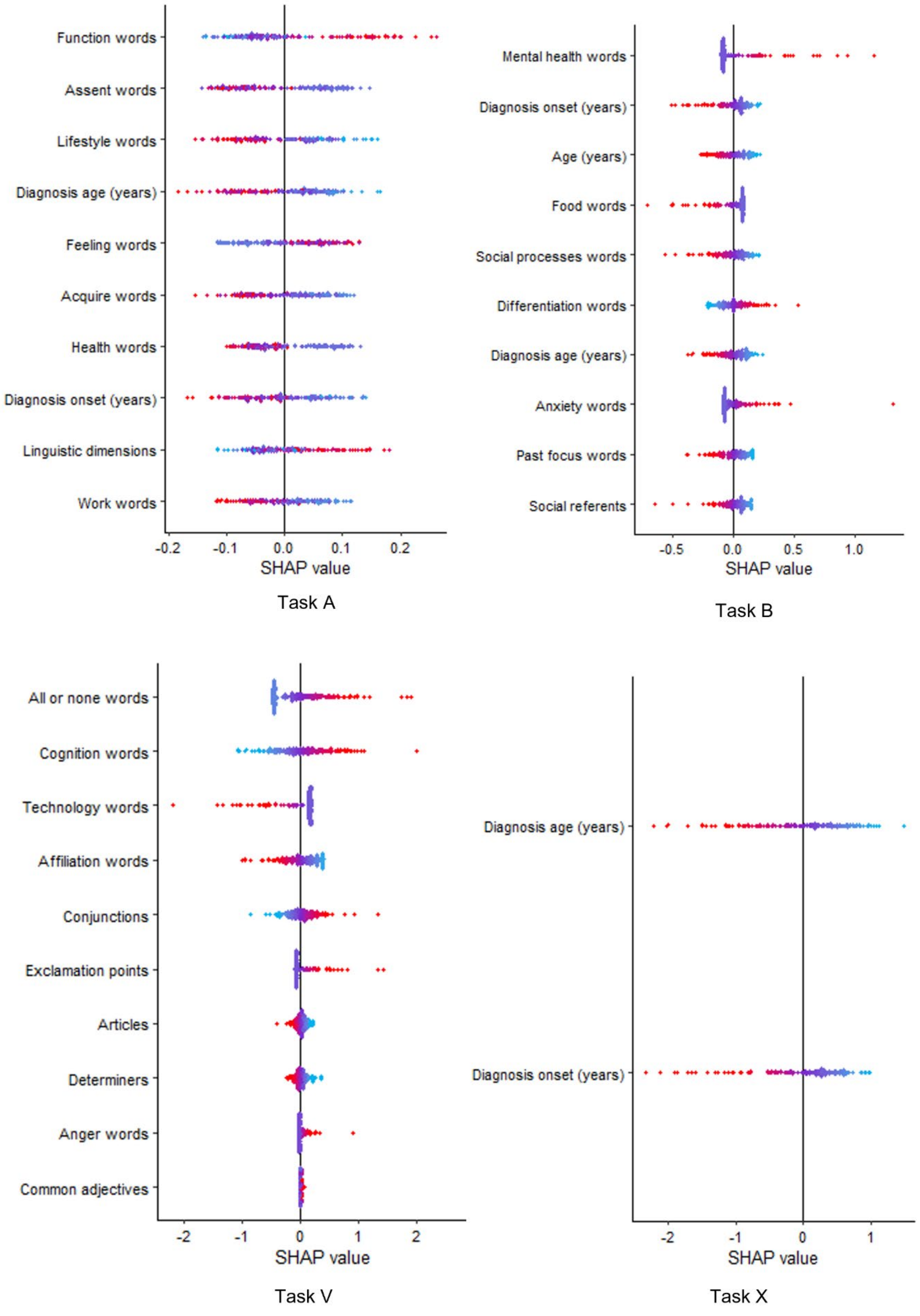

Figure Legend: Higher SHAP values indicate greater importance to the modelling. Blue indicates low importance, Red indicates high importance.

Figure S16. Variable stability plots for best performing machine learning models within tasks predicting depressive symptoms within Task A.

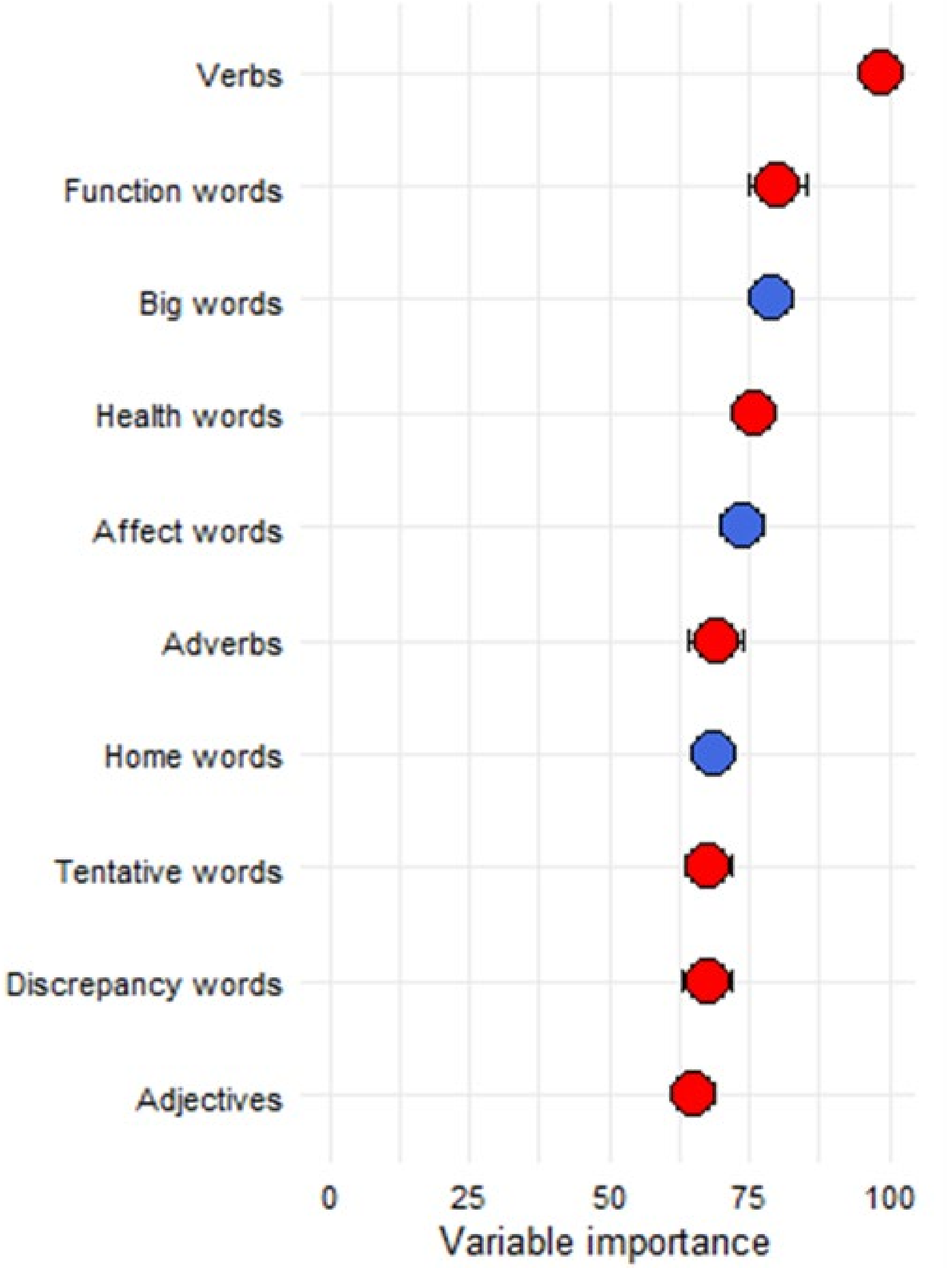

Figure Legend: Higher values indicate greater importance to the modelling. Blue indicates features that have negative associations with symptoms, Red indicates features that have positive associations with symptoms.

Figure S17. Variable stability plots for best performing machine learning models within tasks predicting depressive symptoms within Task B.

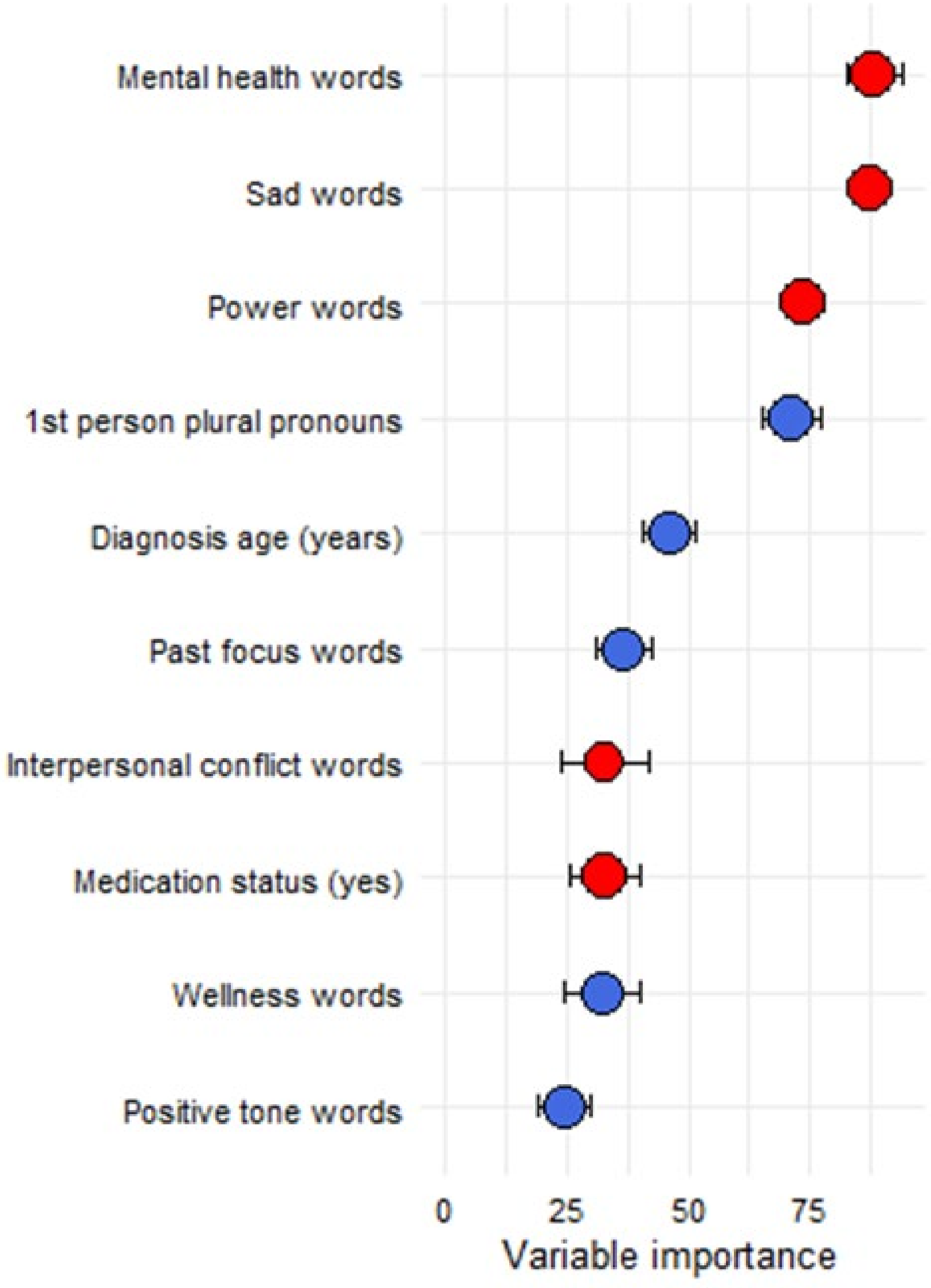

Figure Legend: Higher values indicate greater importance to the modelling. Blue indicates features that have negative associations with symptoms, Red indicates features that have positive associations with symptoms.

Figure S18. Variable stability plots for best performing machine learning models within tasks predicting depressive symptoms within Task C.

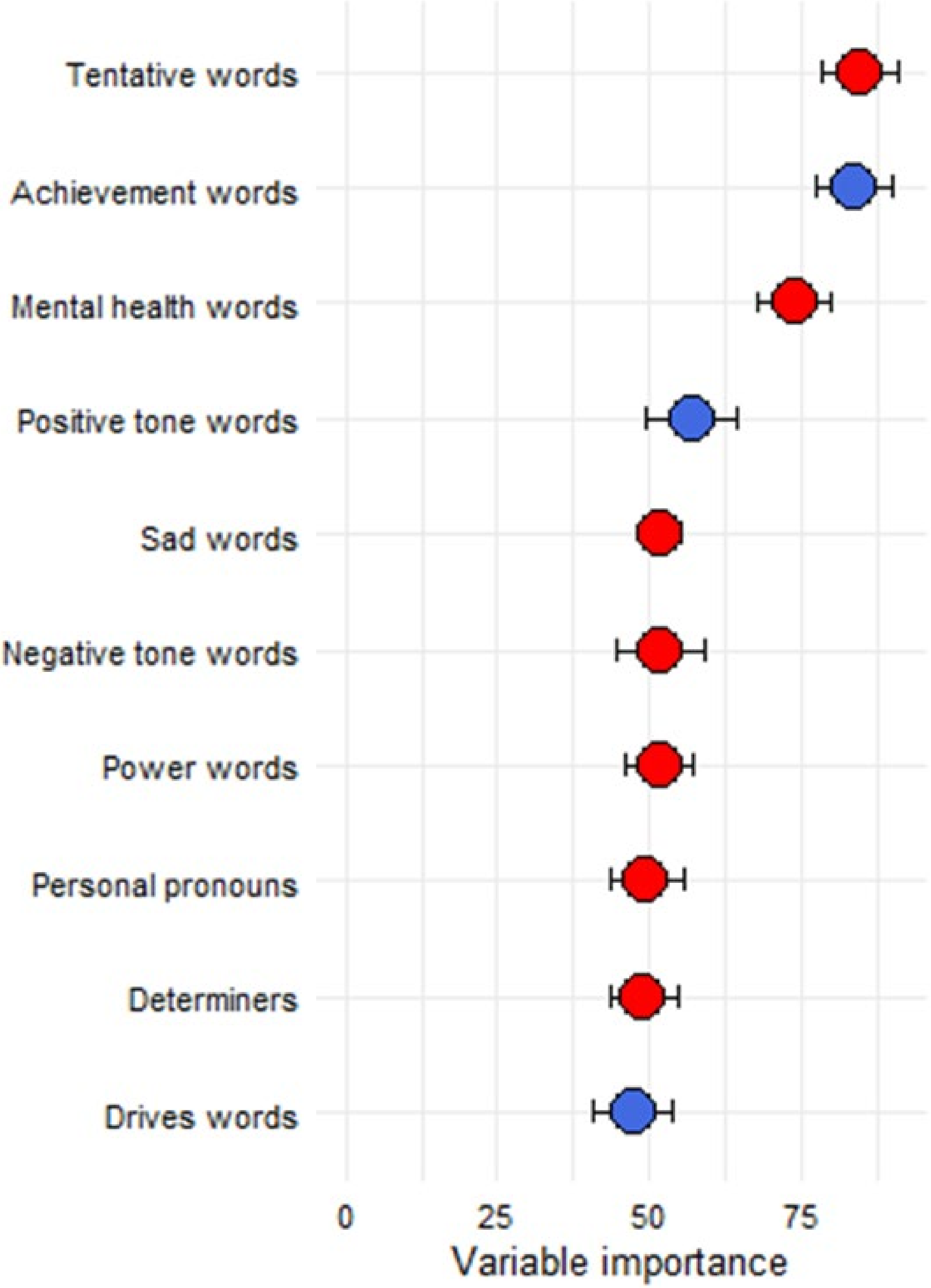

Figure Legend: Higher values indicate greater importance to the modelling. Blue indicates features that have negative associations with symptoms, Red indicates features that have positive associations with symptoms.

Figure S19. Variable stability plots for best performing machine learning models within tasks predicting depressive symptoms within Task D.

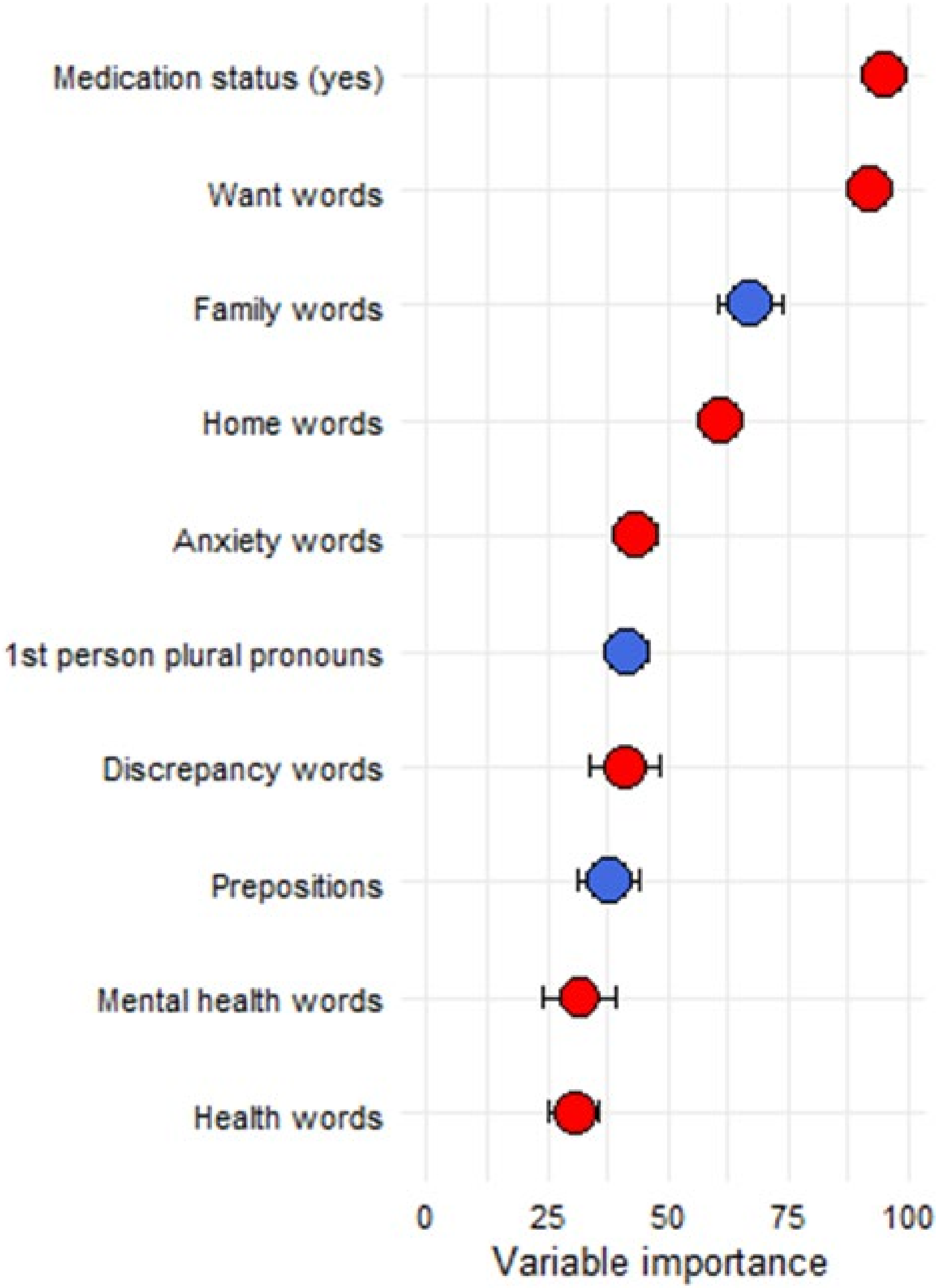

Figure Legend: Higher values indicate greater importance to the modelling. Blue indicates features that have negative associations with symptoms, Red indicates features that have positive associations with symptoms.

Figure S20. Variable stability plots for best performing machine learning models within tasks predicting depressive symptoms within Task V.

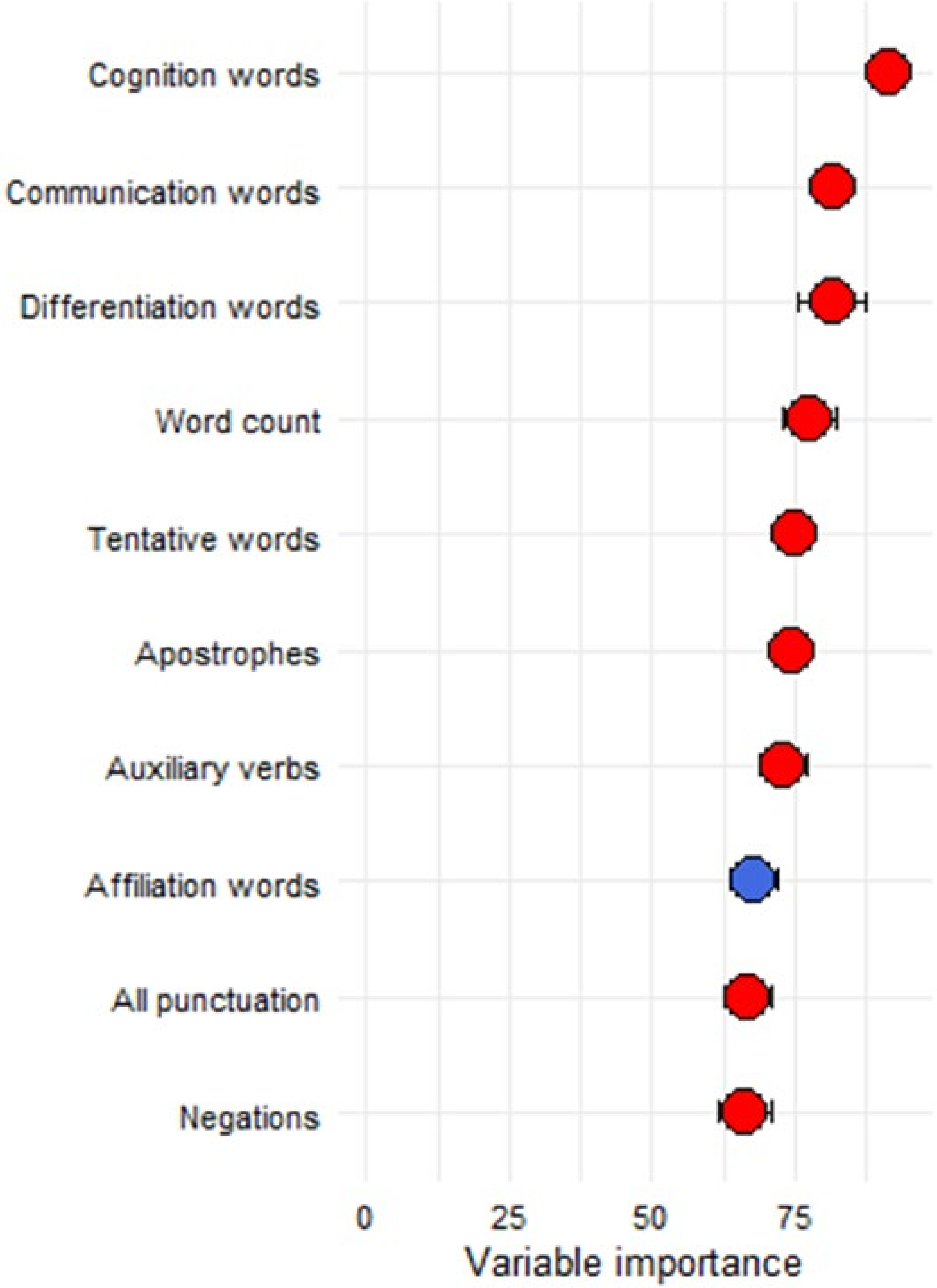

Figure Legend: Higher values indicate greater importance to the modelling. Blue indicates features that have negative associations with symptoms, Red indicates features that have positive associations with symptoms.

Figure S21. Variable stability plots for best performing machine learning models within tasks predicting depressive symptoms within Task W.

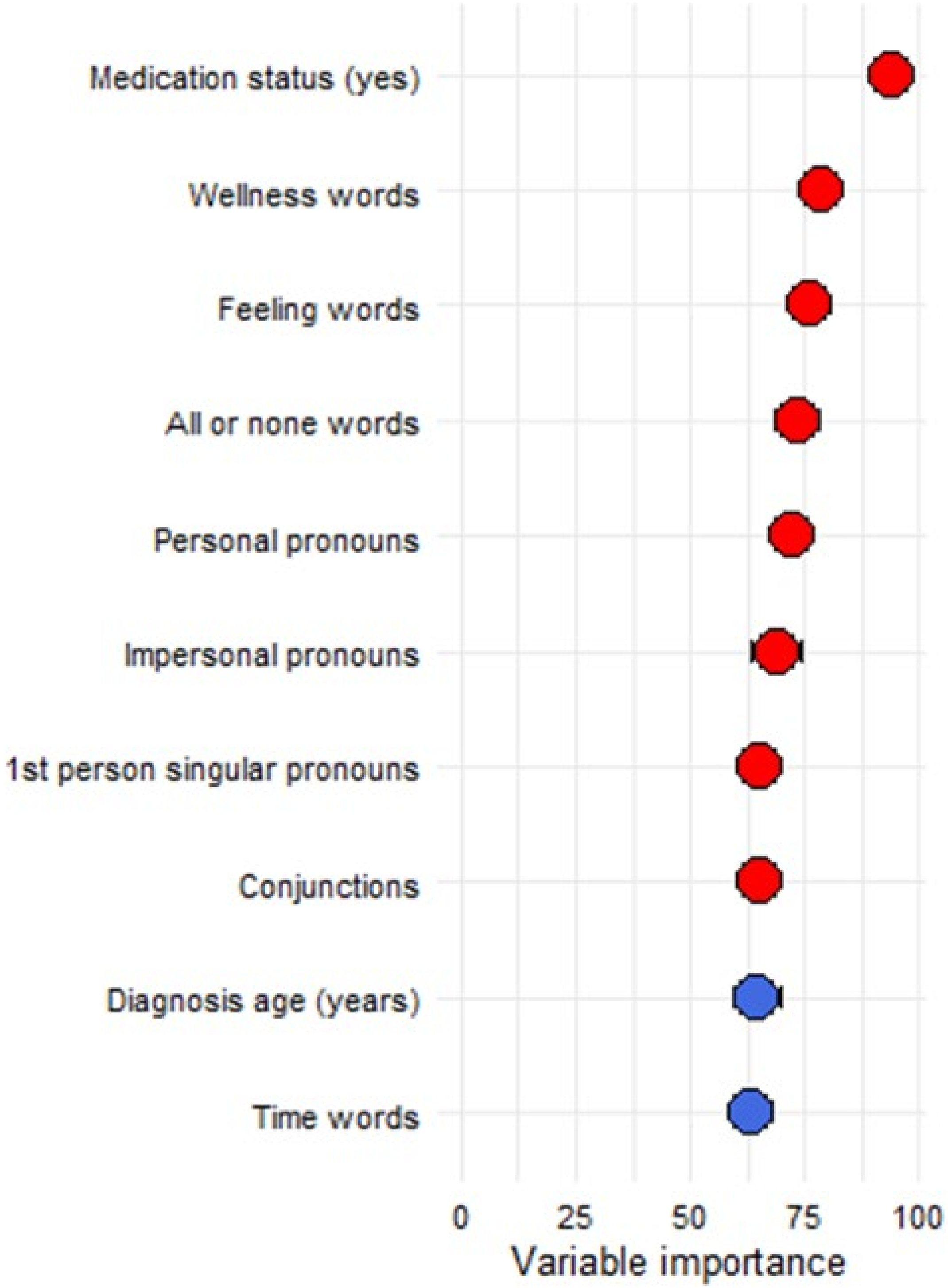

Figure Legend: Higher values indicate greater importance to the modelling. Blue indicates features that have negative associations with symptoms, Red indicates features that have positive associations with symptoms.

Figure S22. Variable stability plots for best performing machine learning models within tasks predicting depressive symptoms within Task X.

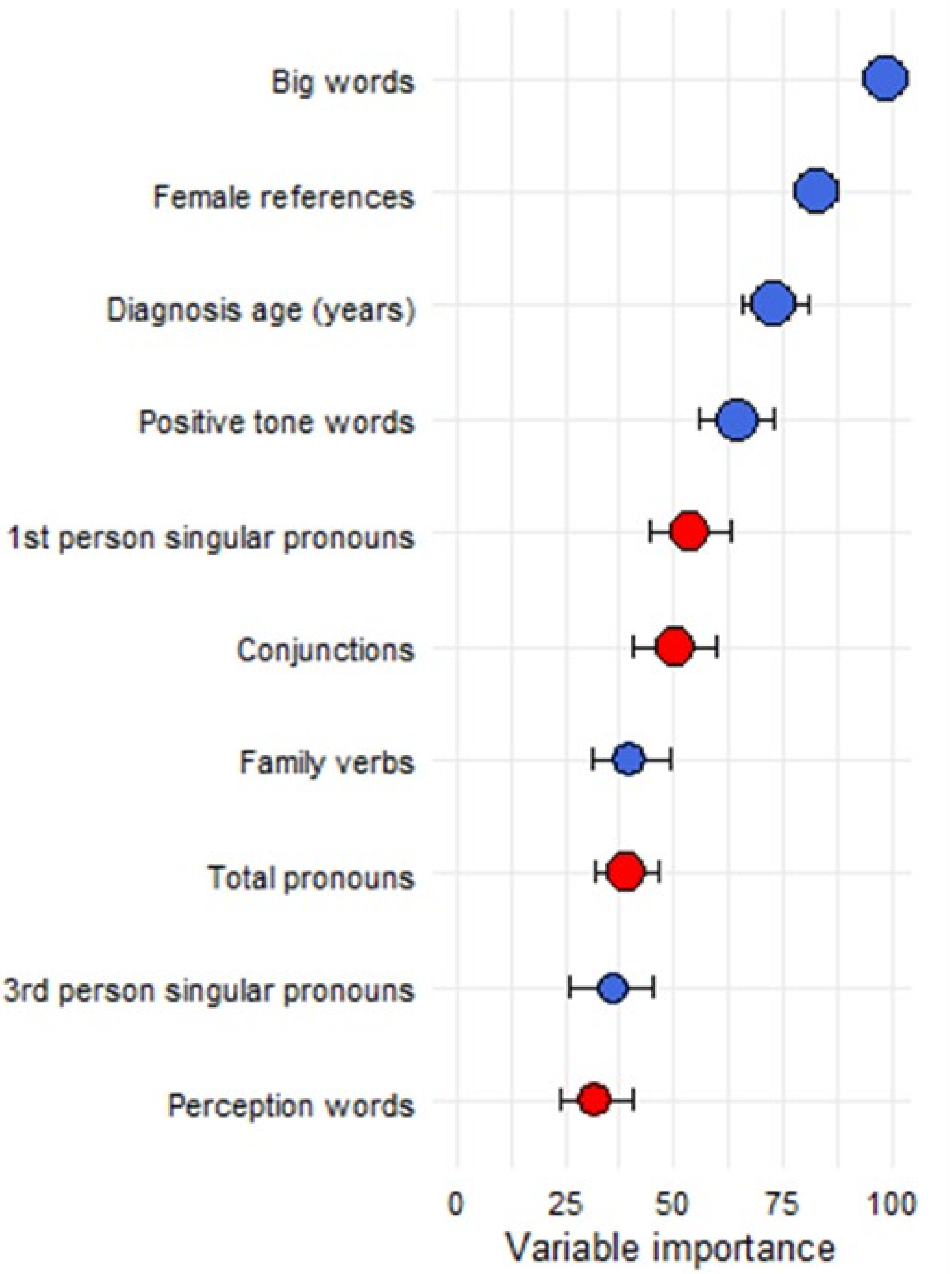

Figure Legend: Higher values indicate greater importance to the modelling. Blue indicates features that have negative associations with symptoms, Red indicates features that have positive associations with symptoms.

Figure S23. Variable stability plots for best performing machine learning models within tasks predicting depressive symptoms within Task Y.

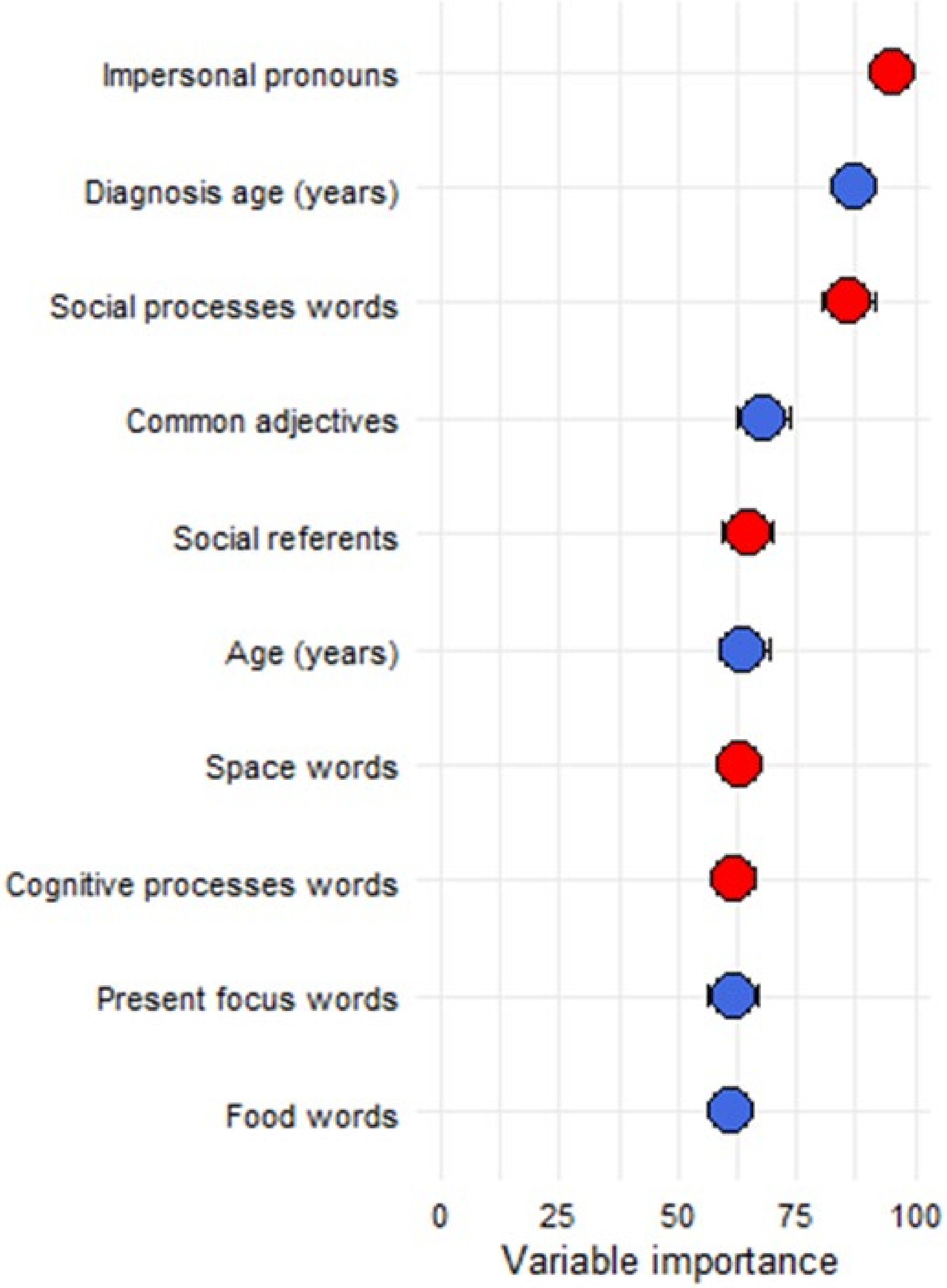

Figure Legend: Higher values indicate greater importance to the modelling. Blue indicates features that have negative associations with symptoms, Red indicates features that have positive associations with symptoms.

Figure S24. Variable stability plots for best performing machine learning models within tasks predicting anxiety symptoms in Task A.

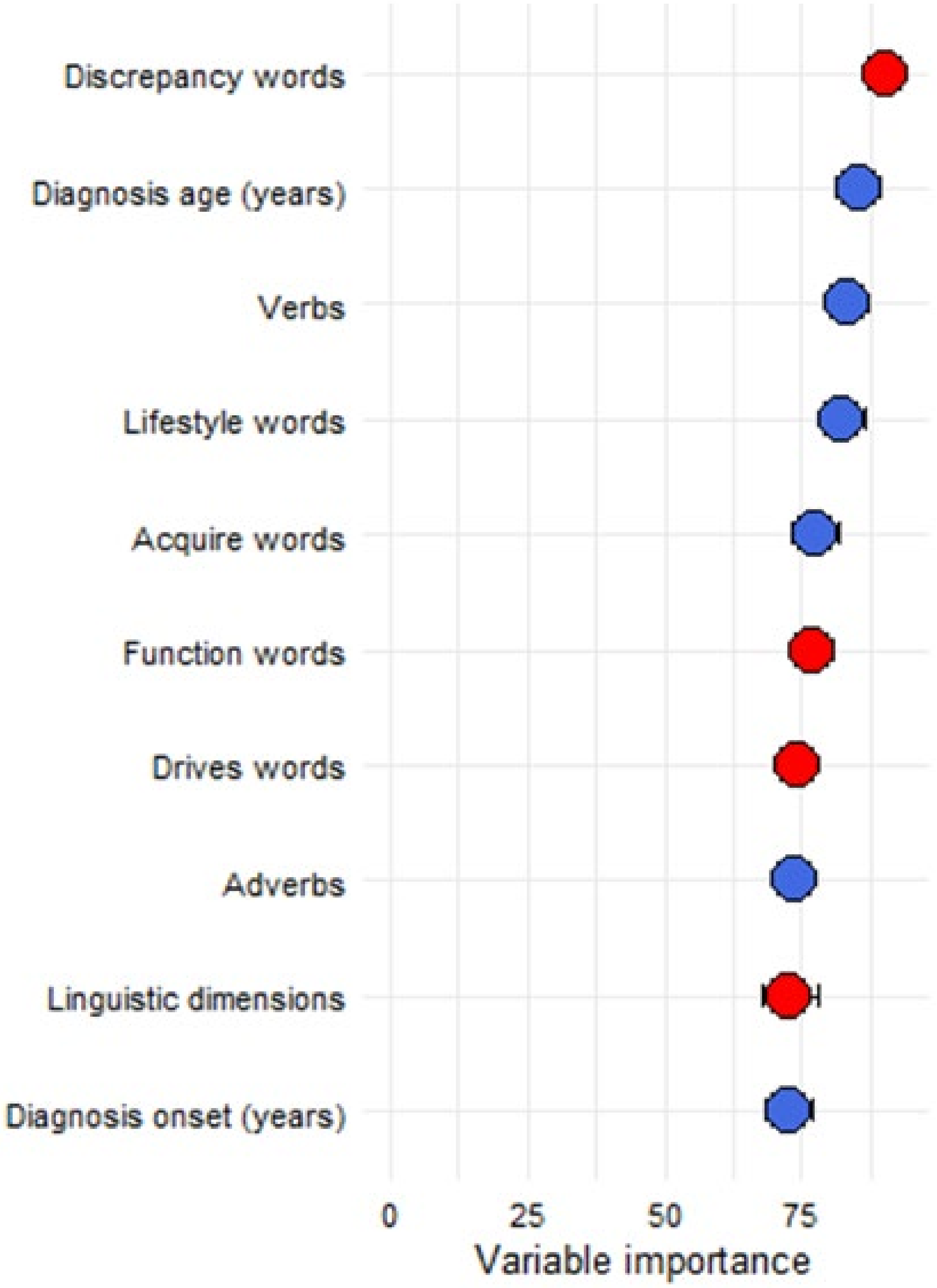

Figure Legend: Higher values indicate greater importance to the modelling. Blue indicates features that have negative associations with symptoms, Red indicates features that have positive associations with symptoms.

Figure S25. Variable stability plot for best performing machine learning models within tasks predicting anxiety symptoms in Task B.

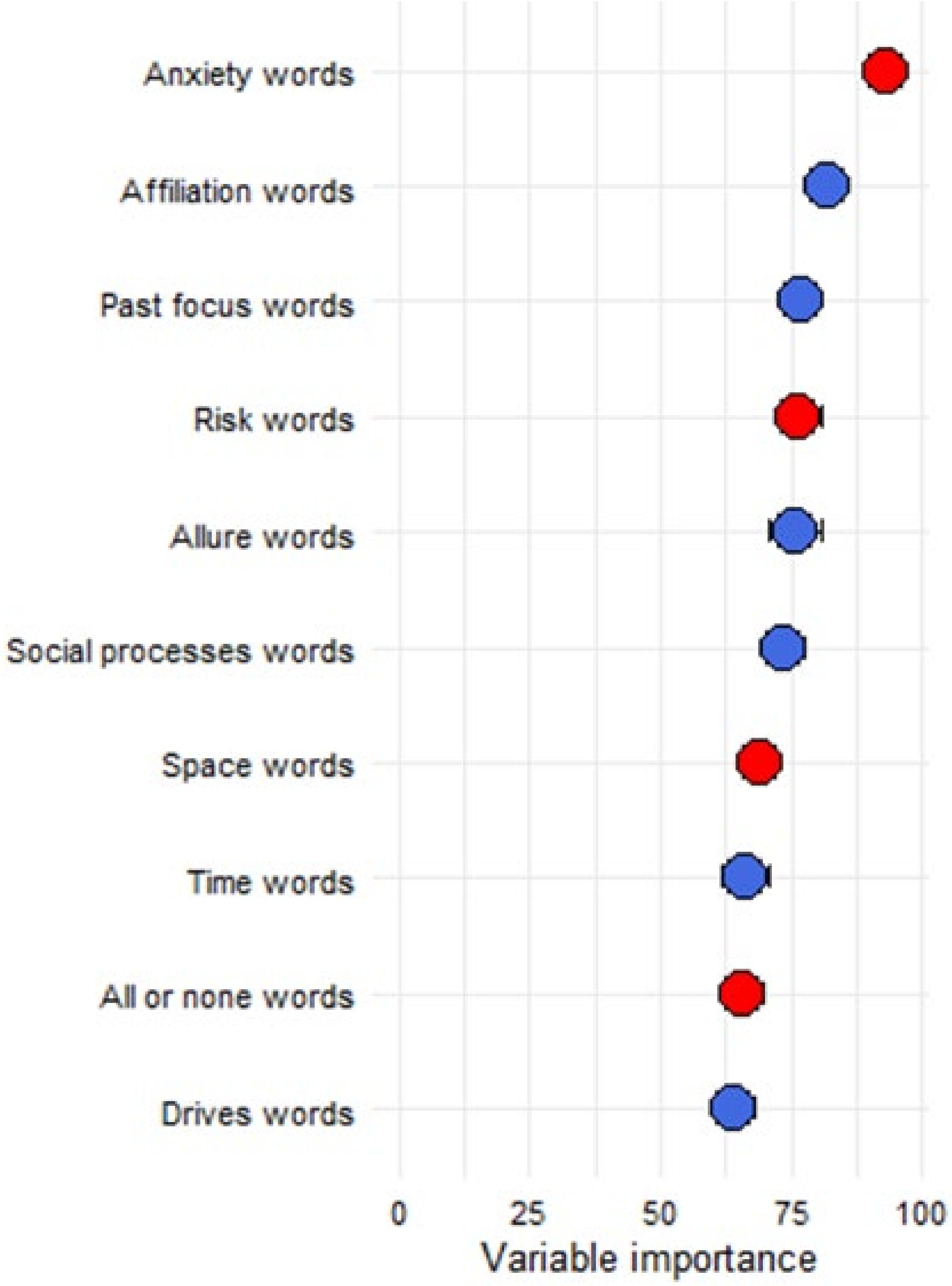

Figure Legend: Higher values indicate greater importance to the modelling. Blue indicates features that have negative associations with symptoms, Red indicates features that have positive associations with symptoms.

Figure S26. Variable stability plot for best performing machine learning models within tasks predicting anxiety symptoms in Task V.

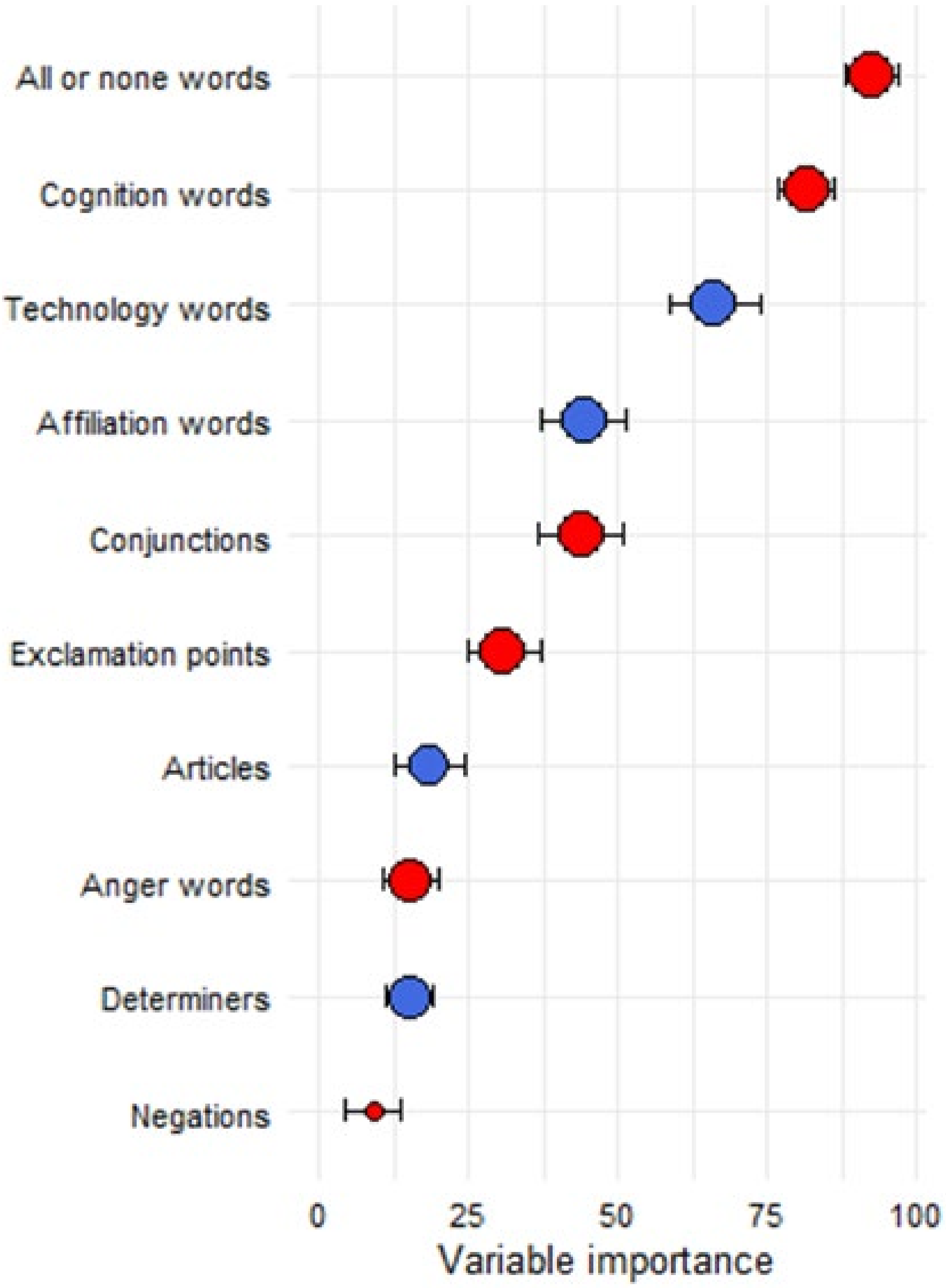

Figure Legend: Higher values indicate greater importance to the modelling. Blue indicates features that have negative associations with symptoms, Red indicates features that have positive associations with symptoms.

Figure S27. Variable stability plot for best performing machine learning models within tasks predicting anxiety symptoms in Task X.

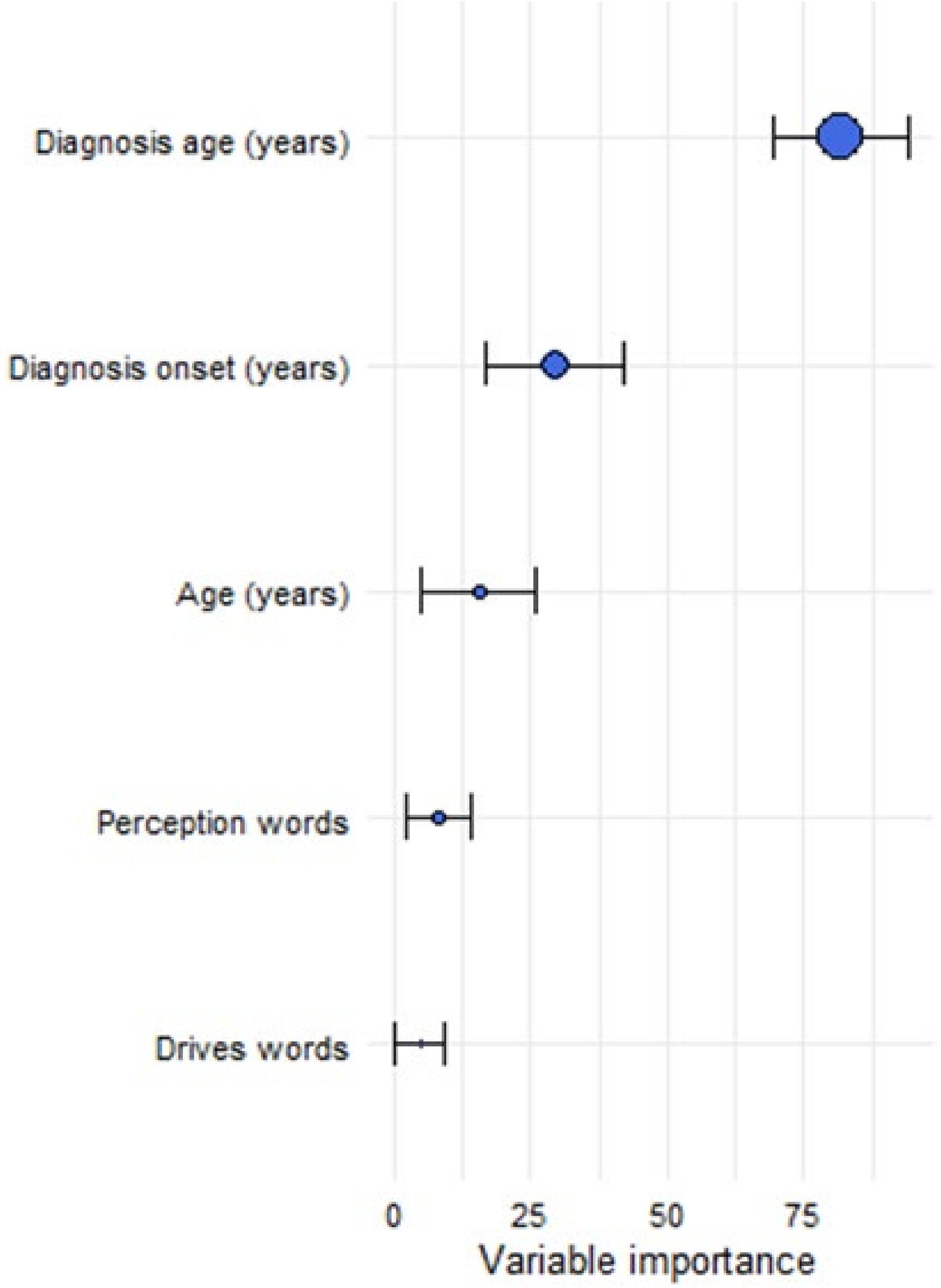

Figure Legend: Higher values indicate greater importance to the modelling. Blue indicates features that have negative associations with symptoms, Red indicates features that have positive associations with symptoms.

Figure S28. Coefficient values from best performing elastic net models for predicting depressive symptoms (PHQ-9) and anxiety symptoms (GAD-7) within Task B.

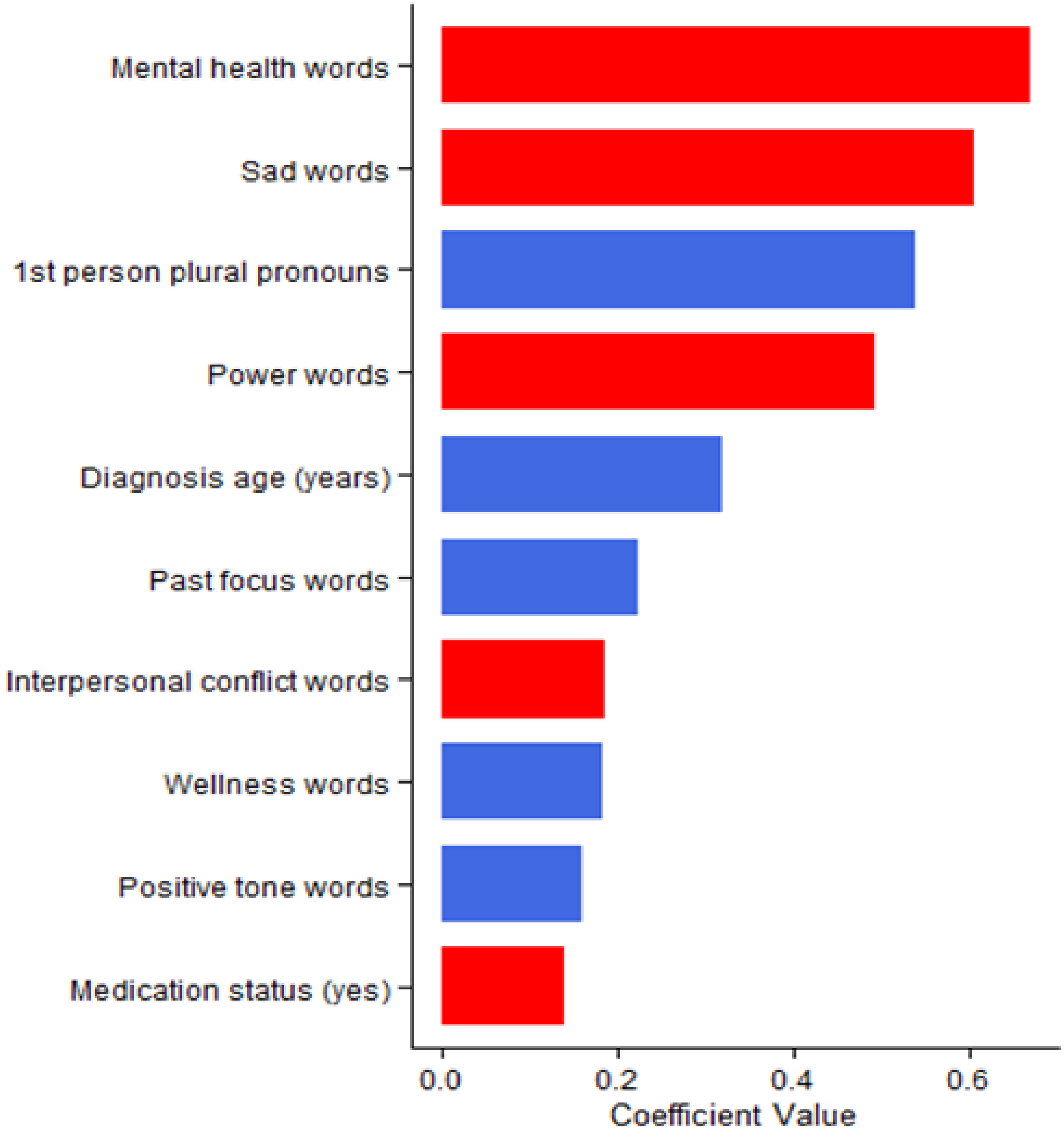

Figure Legend: Higher values indicate greater contribution to the modelling. Blue indicates low importance, Red indicates high importance.

Figure S29. Coefficient values from best performing elastic net models for predicting depressive symptoms (PHQ-9) and anxiety symptoms (GAD-7) within Task V.

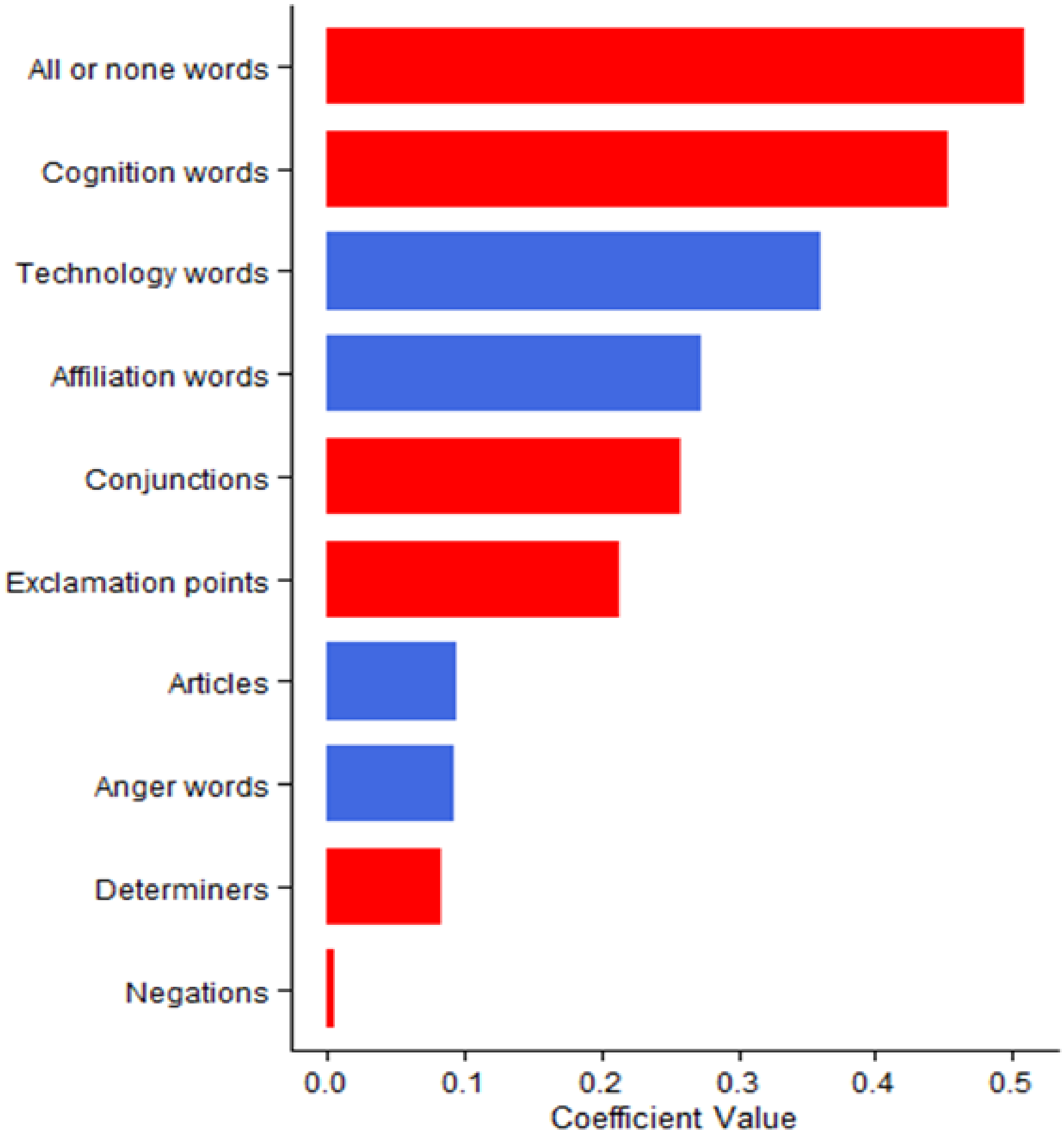

Figure Legend: Higher values indicate greater contribution to the modelling. Blue indicates low importance, Red indicates high importance.

Figure S30. Coefficient values from best performing SVM-L models for predicting anxiety (GAD-7) symptoms within Task B.

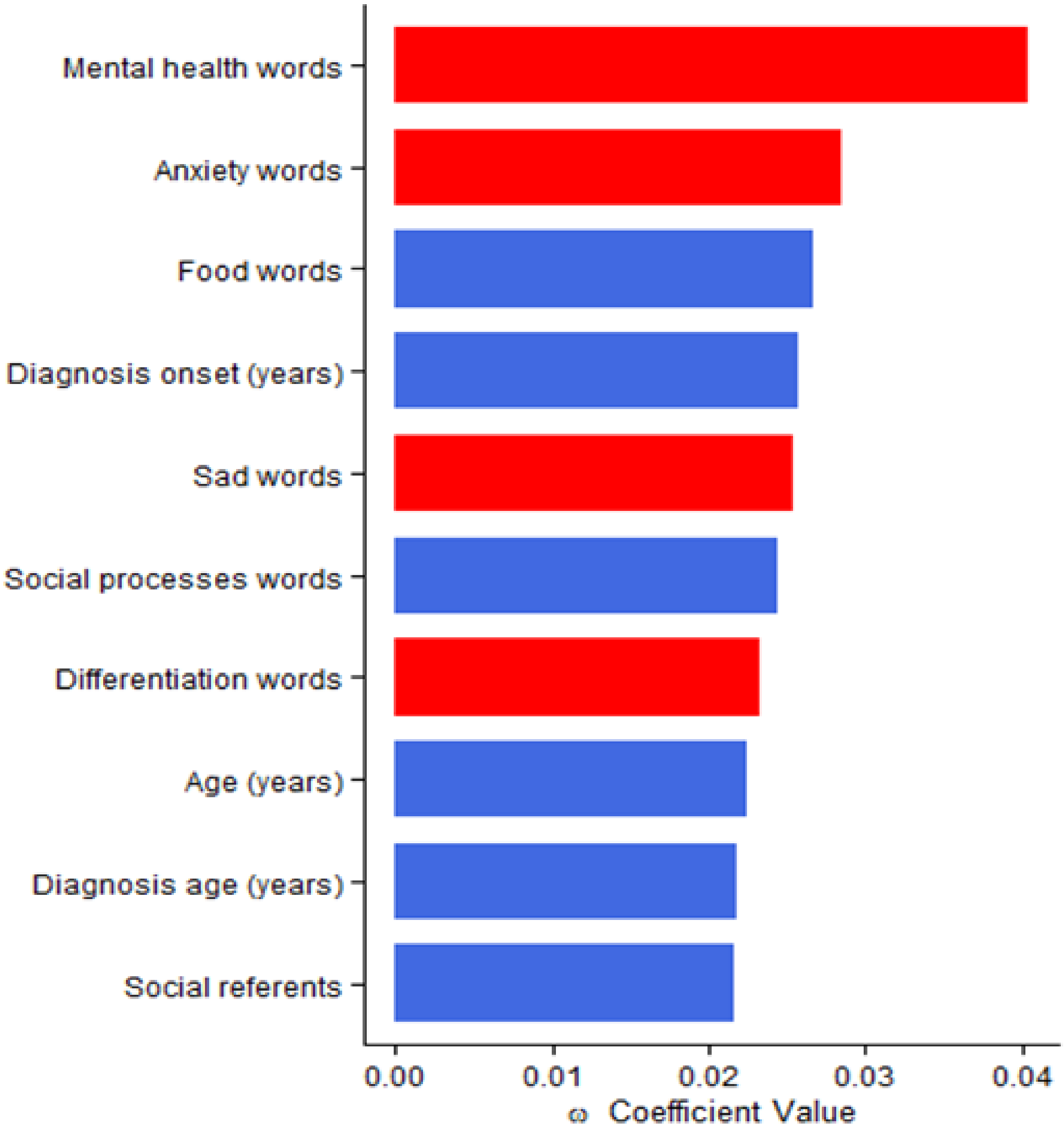

Figure Legend: Higher values indicate greater contribution to the modelling. Blue indicates low importance, Red indicates high importance.

Figure S31. Coefficient values from best performing SVM-L models for predicting anxiety (GAD-7) symptoms within Task X.

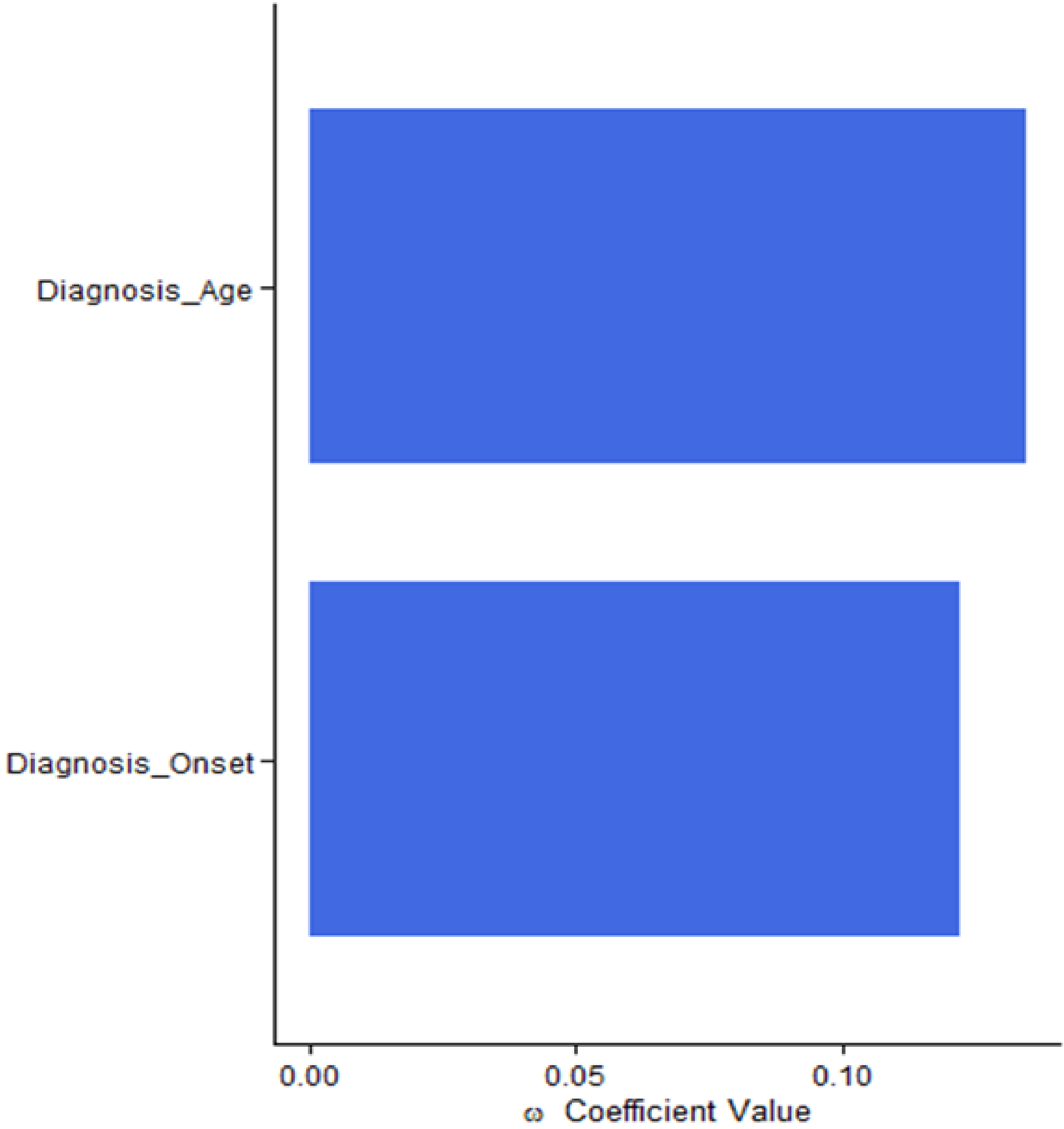

Figure Legend: Higher values indicate greater contribution to the modelling. Blue indicates low importance, Red indicates high importance.

Figure S32. Scatterplot of the significant correlations between first-person plural pronours and depressive symptoms (PHQ-9) when all tasks combined.

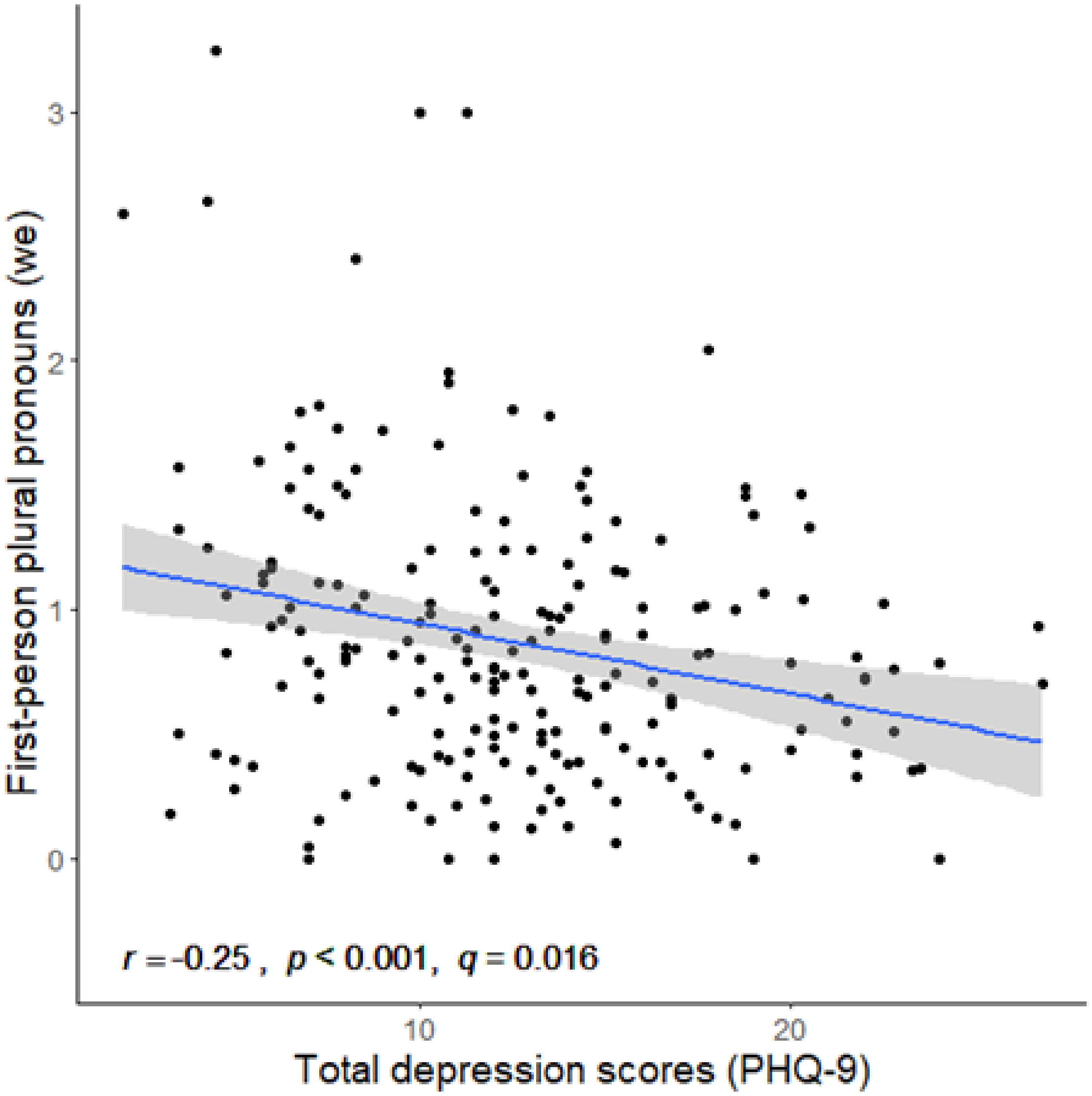

Figure S33. Scatterplot of the significant correlations between affiliation words and depressive symptoms (PHQ-9) when all tasks combined.

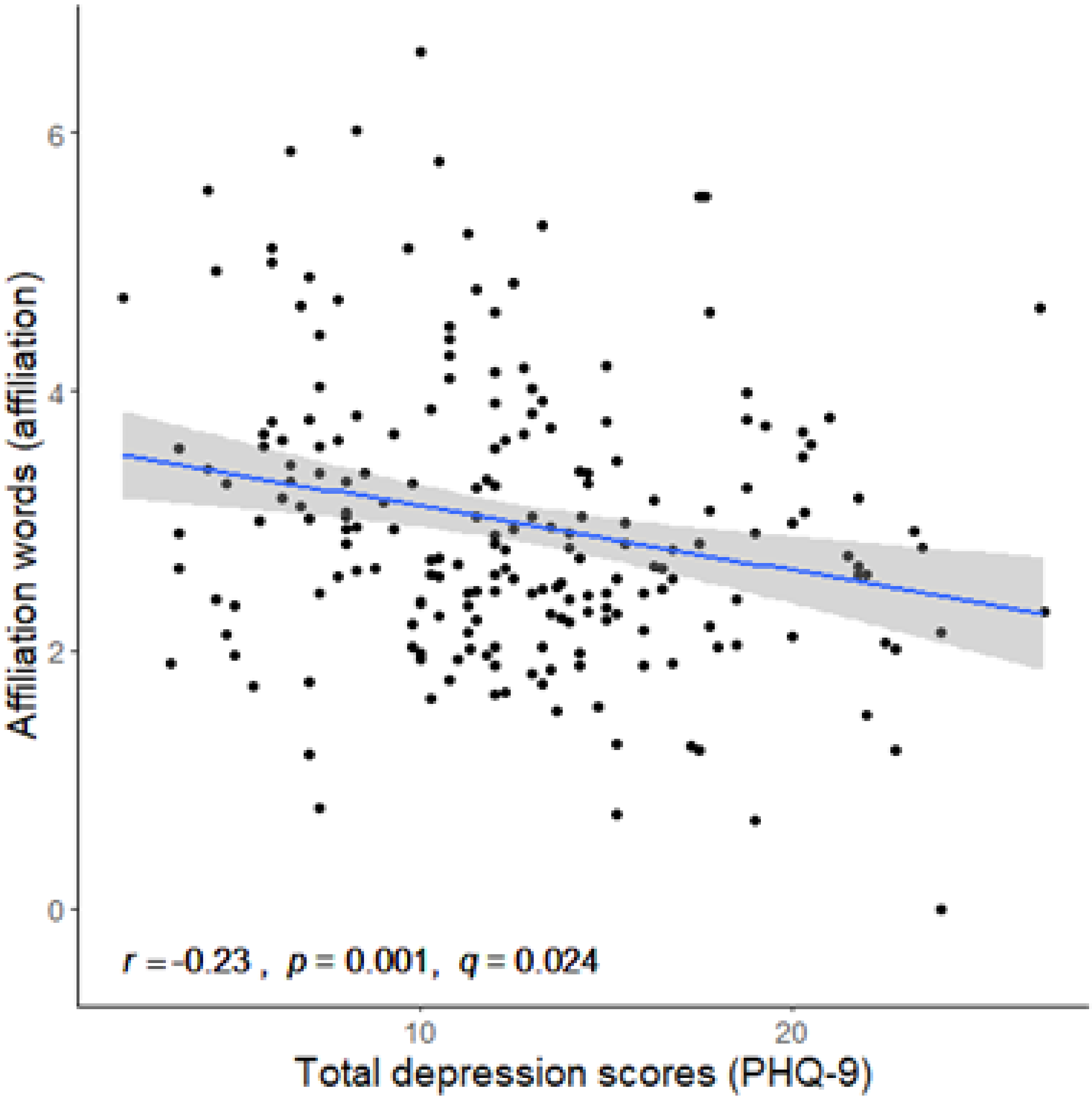

Figure S34. Scatterplot of the significant correlations between cognition words and depressive symptoms (PHQ-9) when all tasks combined.

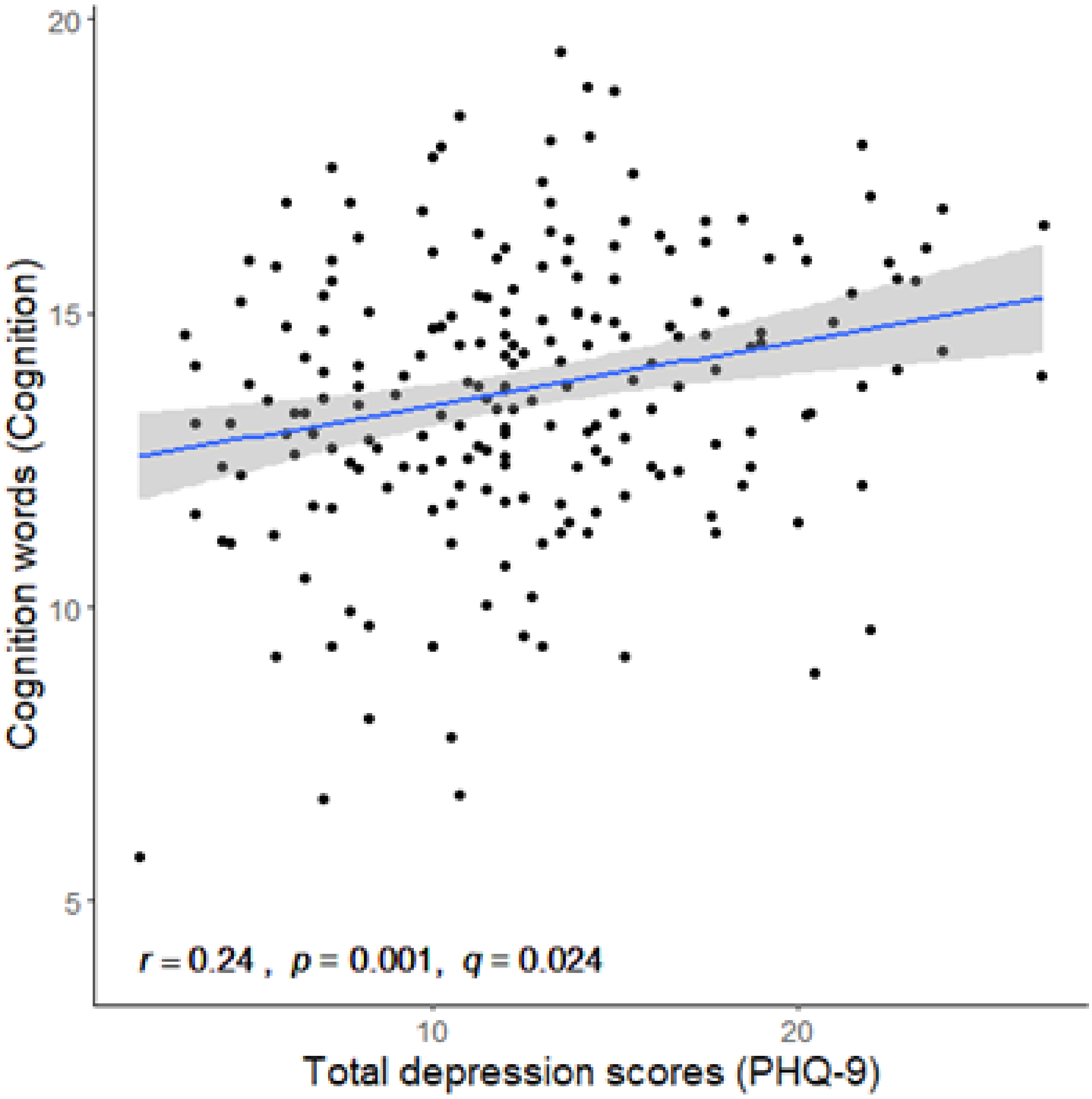

Figure S35. Scatterplot of the significant correlations between cognitive process words and depressive symptoms (PHQ-9) when all tasks combined.

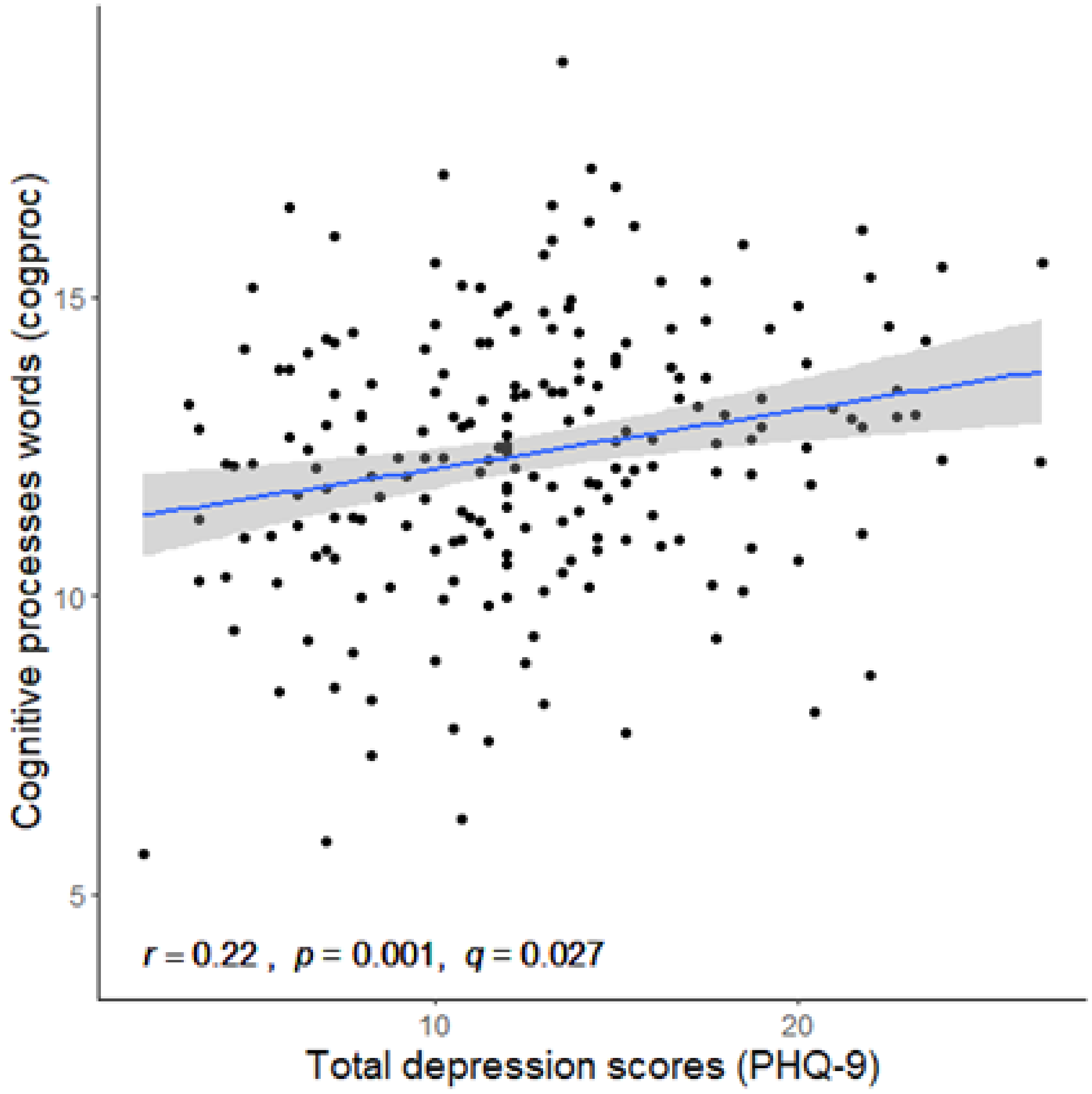

Figure S36. Scatterplot of the significant correlations between postive tone words and depressive symptoms (PHQ-9) when all tasks combined.

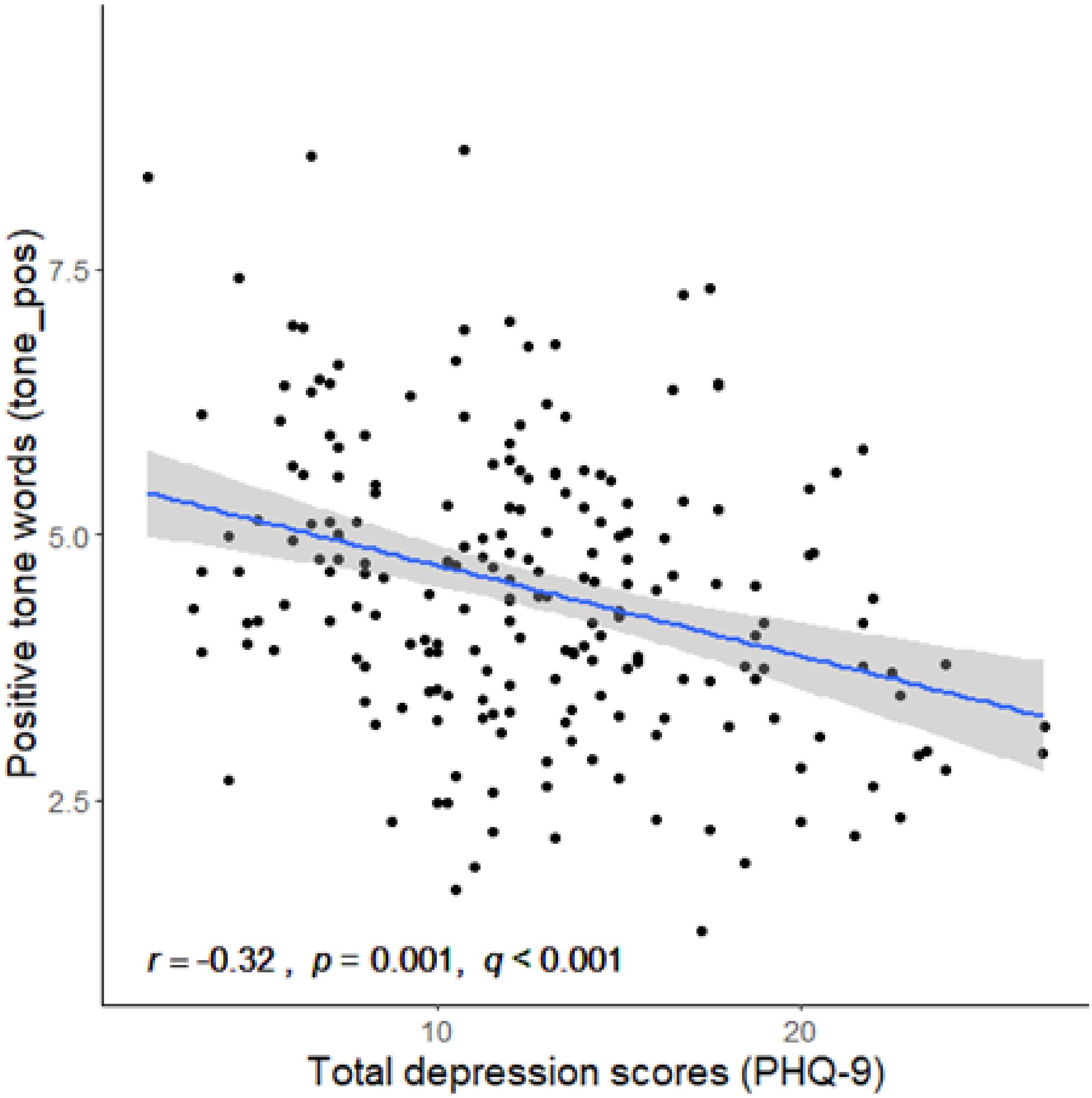

Figure S37. Scatterplot of the significant correlations between postive emotion words and depressive symptoms (PHQ-9) when all tasks combined.

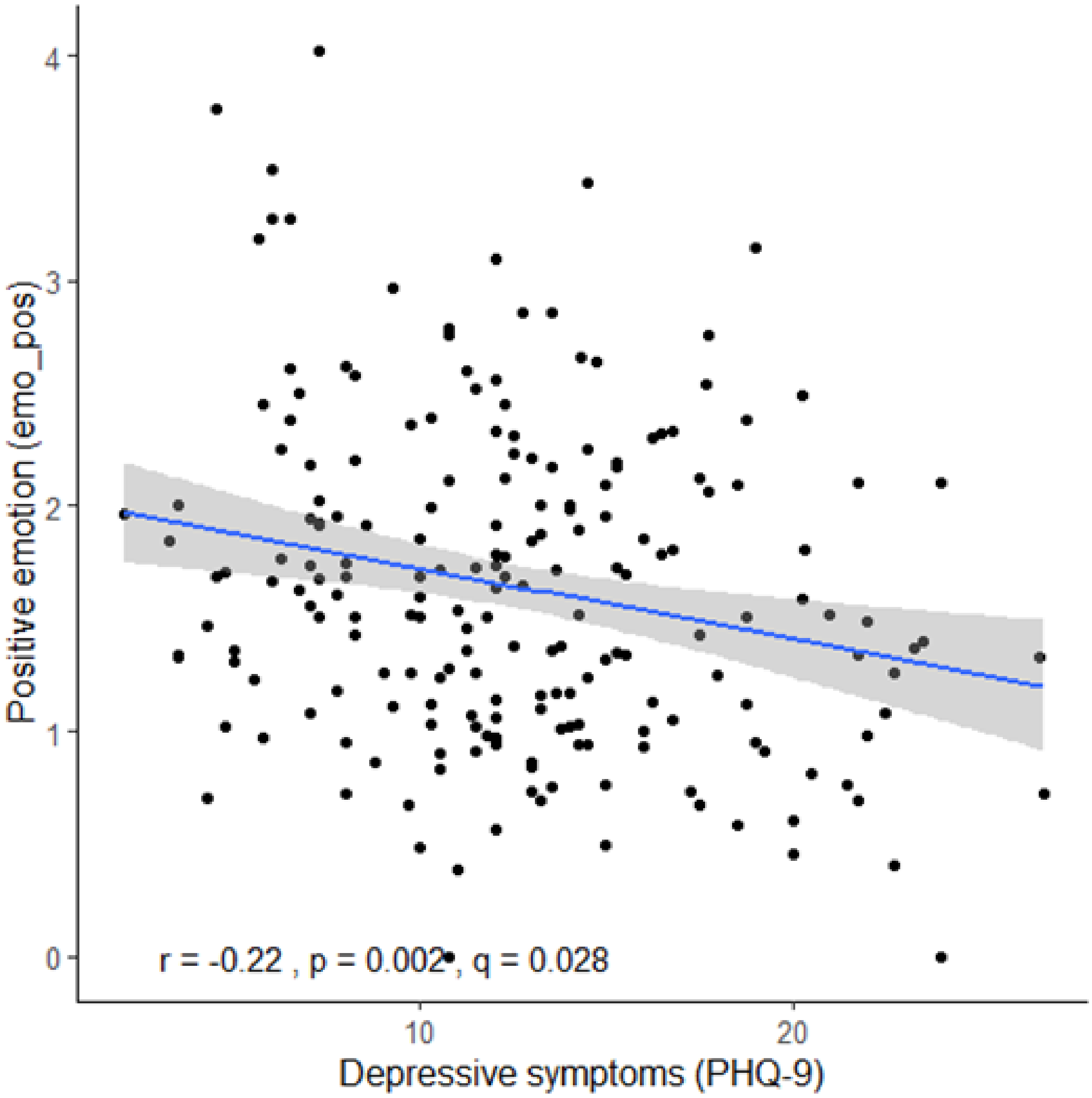

Figure S38. Scatterplot of the significant correlations between cognition words and anxiety symptoms (GAD-7) when all tasks combined.

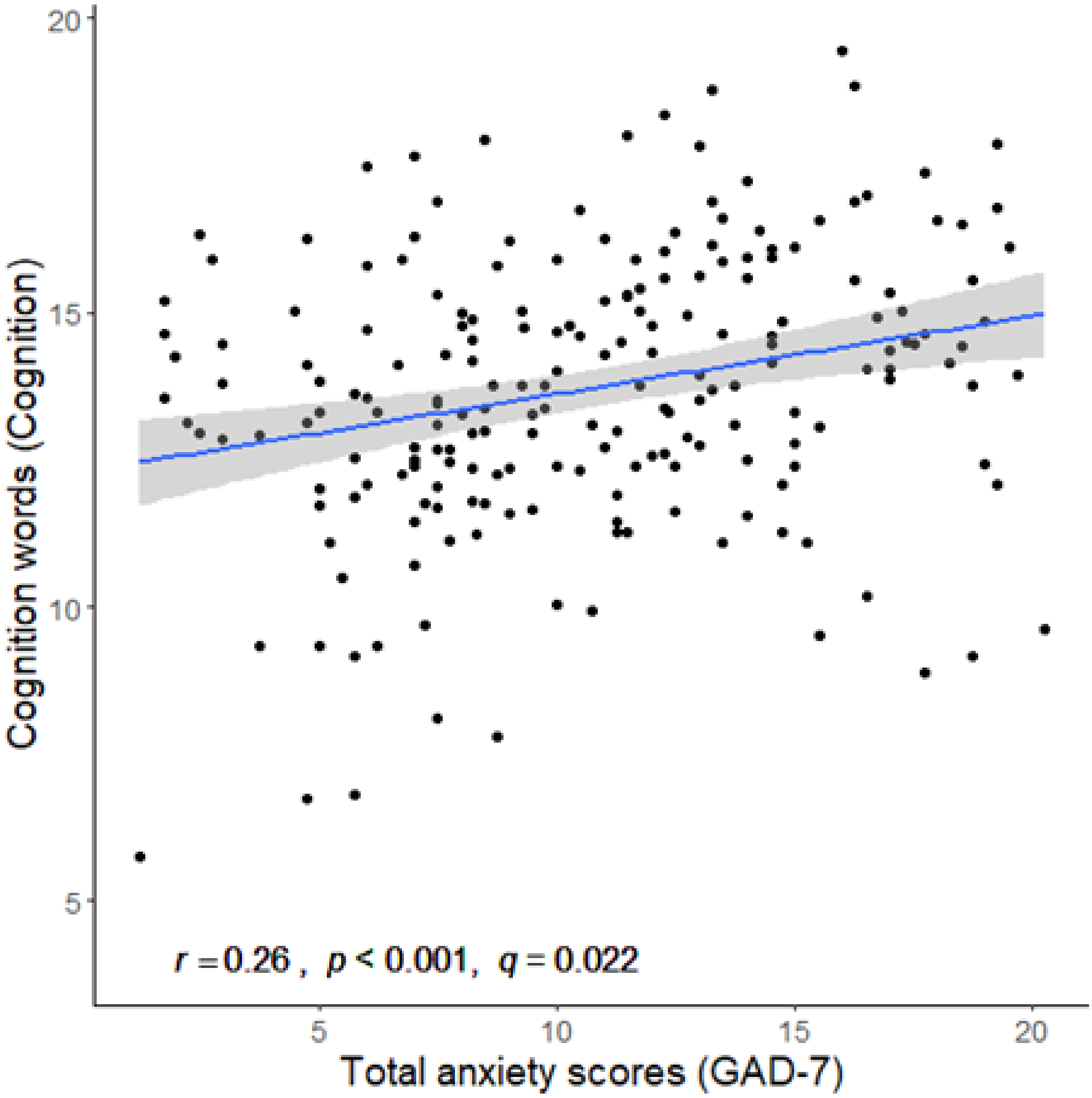

Figure S39. Variable stability plots for best performing machine learning models for depressive symptoms (PHQ-9) when all tasks combined.

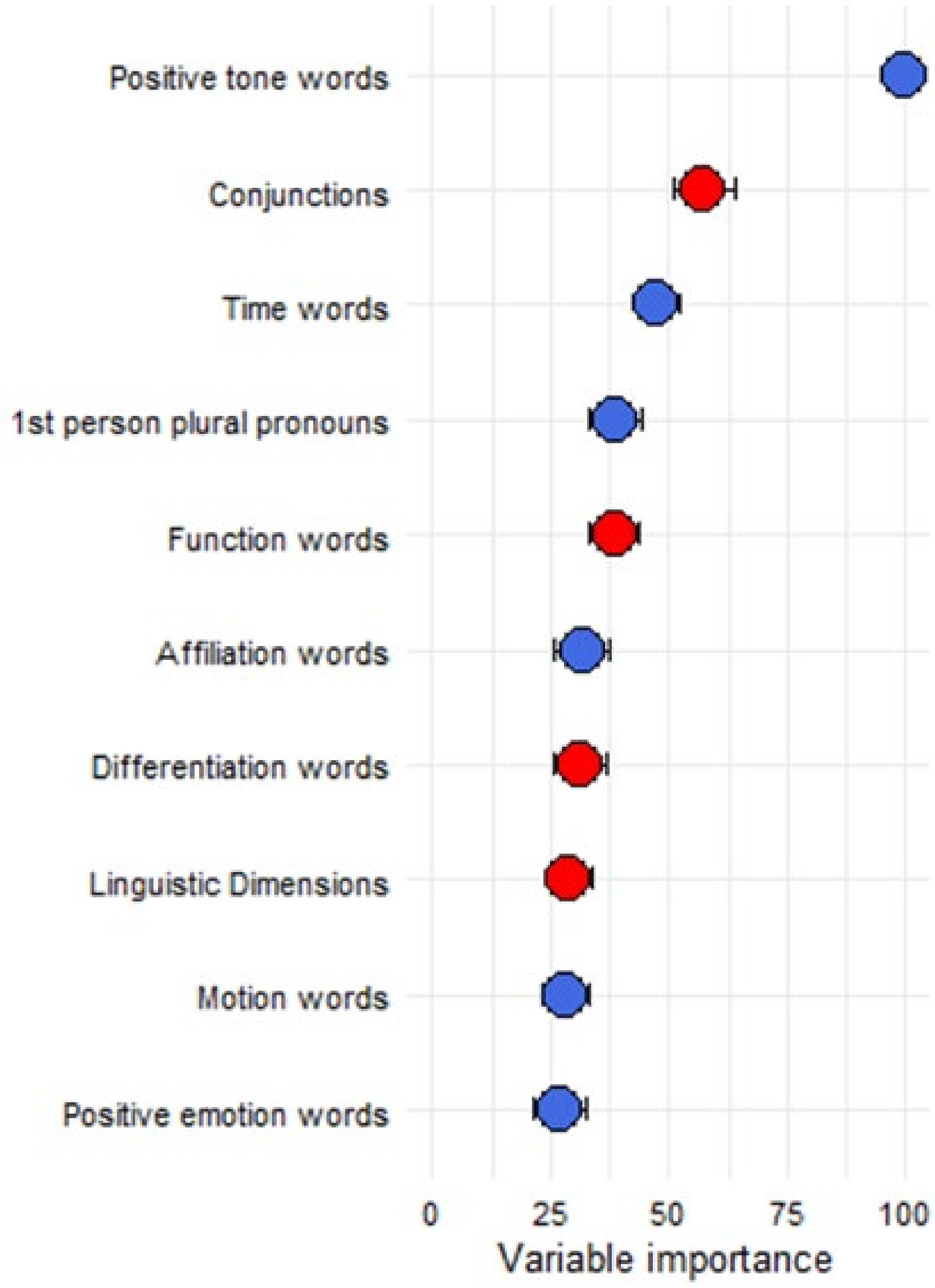

Figure Legend: Higher values indicate greater contribution to the modelling. Blue indicates low importance, Red indicates high importance.

S40. Variable stability plots for best performing machine learning models for depressive symptoms (PHQ-9) when all tasks combined.

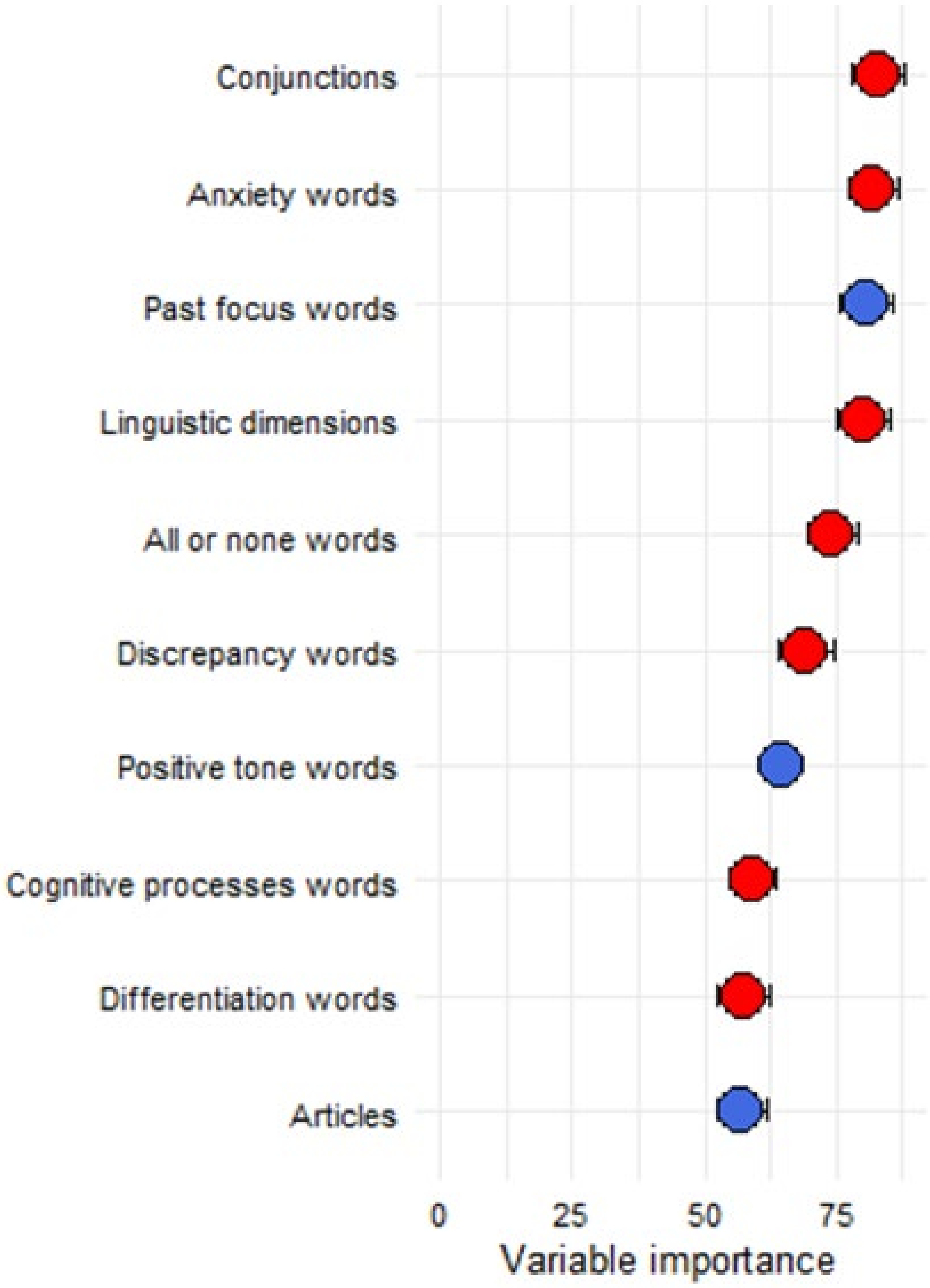

Figure Legend: Higher values indicate greater contribution to the modelling. Blue indicates low importance, Red indicates high importance.

Impact of expressive writing tasks on participants' mood and acceptability ratings

For the expressive writing tasks, participants reported significantly higher levels of fatigue after their first (MD: 0.46, *P*<.001), second (MD: 0.30, *P*=.030) and third completions (MD: 0.28, *P*=.045). Participants were also significantly less content after their first (MD: 0.60, *P* =.000), second (MD: 0.46, *P* =.005) and third completions (MD: 0.41, *P* =.007). Participants were also significantly more agitated after their first (MD: 0.86, *P* =<.000), second (MD: 0.54, *P* <.001) and third completions (MD: 0.56, *P* <.001). Participants also reported significantly lower levels of energy after their first (MD: 0.34, *P* =.002), second (MD: 0.29, *P*=.012) and third completions (MD: 0.23, *P*=.040). Participants' also reported that they felt significantly more unwell after their first (MD: 0.47, *P*<.001), second (MD: 0.32, *P*=.006) and third completion (MD: 0.27, *P*=.023). Participants also reported significantly lower levels of relaxation after their first (MD: 0.80, *P* <.001), second (MD: 0.45, *P* <.001) and third completions (MD: 0.34, *P* <.001).

Figure S41. Participants' acceptability scores across tasks only.

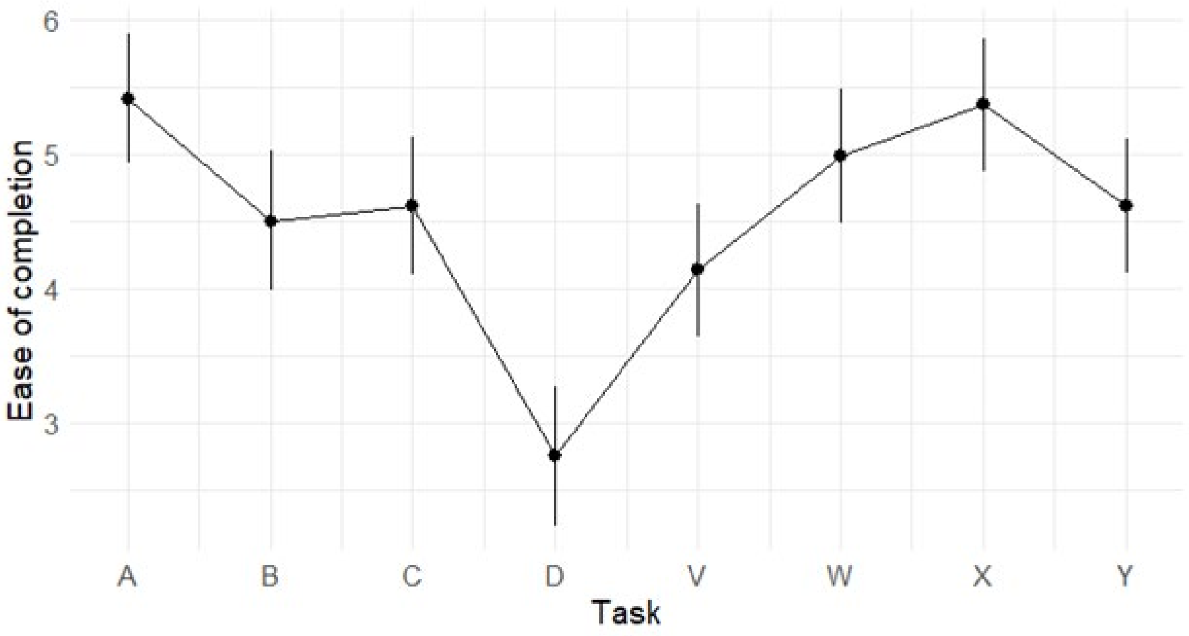

Figure Legend: Differences across sequences are not shown as there was no significant effect for sequence.

Figure S42. Differences in participants' level of interests scores for each of the tasks and across the sequences.

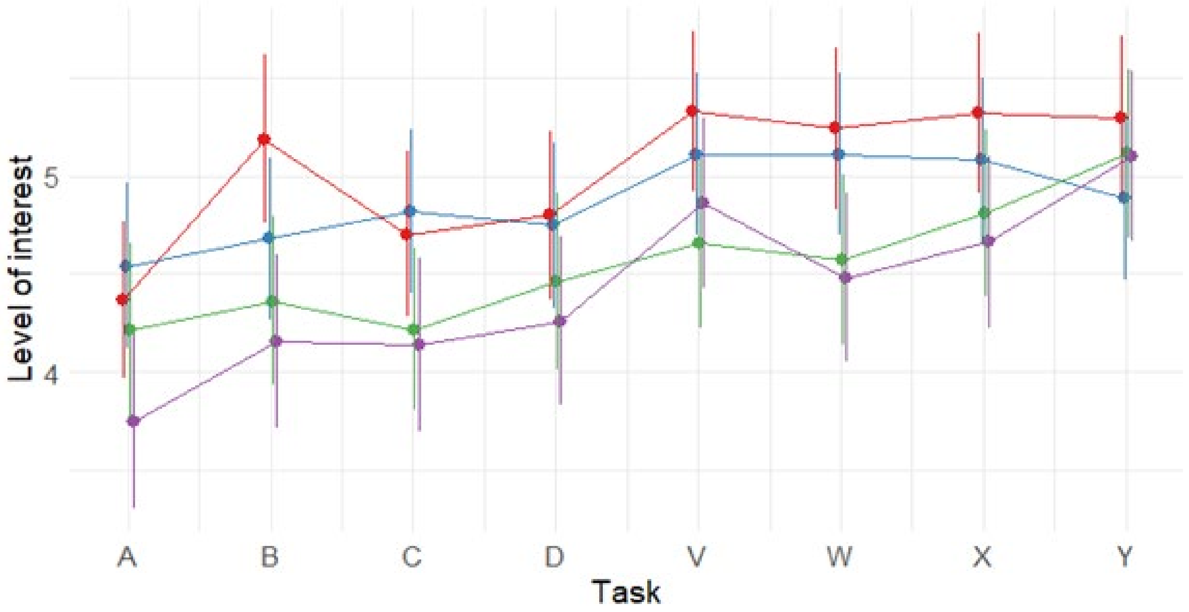

Figure Legend: Red Line indicates Group 1 (Sequence ABCD), Blue line indicates Group 2 (Sequence BCDA), Green line indicates Group 3 (Sequence CDAB), Purple line indicates Group 4 (Sequence DABC).

Figure S43. Differences in participants' willingness to repeat scores for each of the tasks and across the sequences.

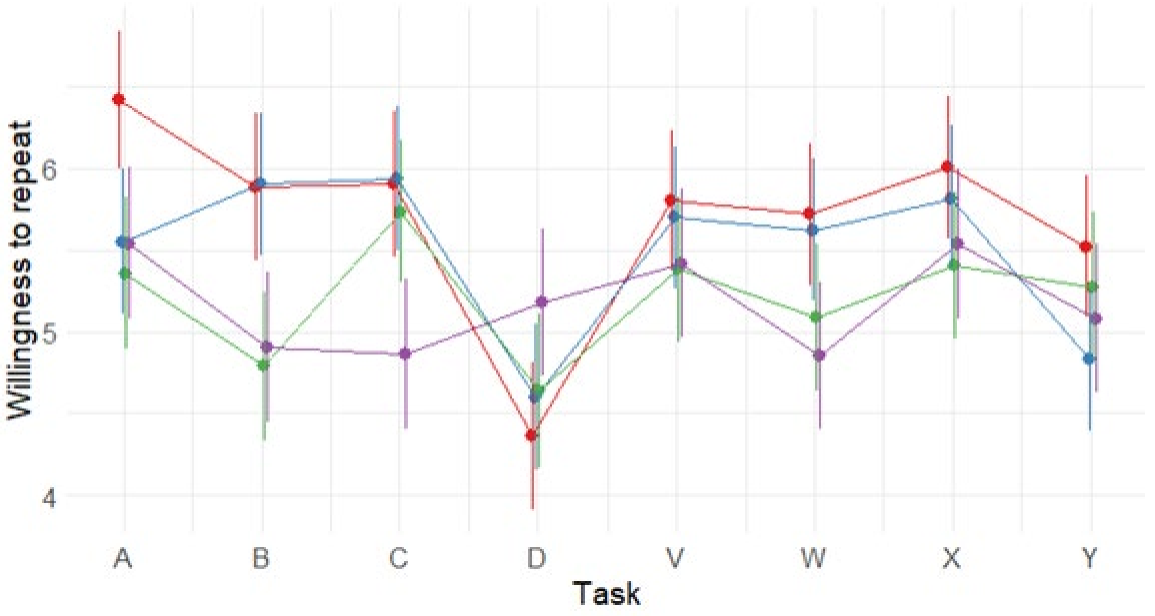

Figure Legend: Red Line indicates Group 1 (Sequence ABCD), Blue line indicates Group 2 (Sequence BCDA), Green line indicates Group 3 (Sequence CDAB), Purple line indicates Group 4 (Sequence DABC).

CONSORT 2010 checklist of information to include when reporting a randomised trial*

Section/Topic	Item No	Checklist item	Reported on page No
**Title and abstract**			
	1a	Identification as a randomised trial in the title	1
	1b	Structured summary of trial design, methods, results, and conclusions (for specific guidance see CONSORT for abstracts)	3
**Introduction**			
Background and objectives	2a	Scientific background and explanation of rationale	4
	2b	Specific objectives or hypotheses	8
**Methods**			
Trial design	3a	Description of trial design (such as parallel, factorial) including allocation ratio	9
	3b	Important changes to methods after trial commencement (such as eligibility criteria), with reasons	NA
Participants	4a	Eligibility criteria for participants	11
	4b	Settings and locations where the data were collected	9,11, 12
Interventions	5	The interventions for each group with sufficient details to allow replication, including how and when they were actually administered	11,12
Outcomes	6a	Completely defined pre-specified primary and secondary outcome measures, including how and when they were assessed	14
	6b	Any changes to trial outcomes after the trial commenced, with reasons	NA
Sample size	7a	How sample size was determined	11
	7b	When applicable, explanation of any interim analyses and stopping guidelines	NA
Randomisation:			
Sequence generation	8a	Method used to generate the random allocation sequence	9
	8b	Type of randomisation; details of any restriction (such as blocking and block size)	9
Allocation concealment mechanism	9	Mechanism used to implement the random allocation sequence (such as sequentially numbered containers), describing any steps taken to conceal the sequence until interventions were assigned	9
Implementation	10	Who generated the random allocation sequence, who enrolled participants, and who assigned participants to interventions	9
Blinding	11a	If done, who was blinded after assignment to interventions (for example, participants, care providers, those	9

		assessing outcomes) and how	
	11b	If relevant, description of the similarity of interventions	NA
Statistical methods	12a	Statistical methods used to compare groups for primary and secondary outcomes	16
	12b	Methods for additional analyses, such as subgroup analyses and adjusted analyses	16
**Results**			
Participant flow (a diagram is strongly recommended)	13a	For each group, the numbers of participants who were randomly assigned, received intended treatment, and were analysed for the primary outcome	21
	13b	For each group, losses and exclusions after randomisation, together with reasons	21
Recruitment	14a	Dates defining the periods of recruitment and follow-up	9
	14b	Why the trial ended or was stopped	NA
Baseline data	15	A table showing baseline demographic and clinical characteristics for each group	22
Numbers analysed	16	For each group, number of participants (denominator) included in each analysis and whether the analysis was by original assigned groups	21,22
Outcomes and estimation	17a	For each primary and secondary outcome, results for each group, and the estimated effect size and its precision (such as 95% confidence interval)	21,22
	17b	For binary outcomes, presentation of both absolute and relative effect sizes is recommended	NA
Ancillary analyses	18	Results of any other analyses performed, including subgroup analyses and adjusted analyses, distinguishing pre-specified from exploratory	22-23
Harms	19	All important harms or unintended effects in each group (for specific guidance see CONSORT for harms)	31-32
**Discussion**			
Limitations	20	Trial limitations, addressing sources of potential bias, imprecision, and, if relevant, multiplicity of analyses	33
Generalisability	21	Generalisability (external validity, applicability) of the trial findings	36-37
Interpretation	22	Interpretation consistent with results, balancing benefits and harms, and considering other relevant evidence	36-37
**Other information**			
Registration	23	Registration number and name of trial registry	Abstract, 9
Protocol	24	Where the full trial protocol can be accessed, if available	9
Funding	25	Sources of funding and other support (such as supply of drugs), role of funders	2

Citation: Schulz KF, Altman DG, Moher D, for the CONSORT Group. CONSORT 2010 Statement: updated guidelines for reporting parallel group randomised trials. BMC Medicine. 2010;8:18.
© 2010 Schulz et al. This is an Open Access article distributed under the terms of the Creative Commons Attribution License (http://creativecommons.org/licenses/by/2.0), which permits unrestricted use, distribution, and reproduction in any medium, provided the original work is properly cited.

*We strongly recommend reading this statement in conjunction with the CONSORT 2010 Explanation and Elaboration for important clarifications on all the items. If relevant, we also recommend reading CONSORT extensions for cluster randomised trials, non-inferiority and equivalence trials, non-pharmacological treatments, herbal interventions, and pragmatic trials. Additional extensions are forthcoming: for those and for up-to-date references relevant to this checklist, see www.consort-statement.org.
